# Widely Targeted Metabolomic and Network Pharmacology Analyses of Active Compounds Enriched from Ethanolic Extract of *Oudemansiella raphanipes*

**DOI:** 10.3390/foods14162820

**Published:** 2025-08-14

**Authors:** Zhi Wu, Jin Zhao, Shuang Zhu, Mengxing Chen, Dan Wu, Yiyou Wu, Junbin Lin, Renyun Miao, Rencai Feng, Xiang Li, Bingcheng Gan, Tao Wang

**Affiliations:** 1 Institute of Urban Agriculture, Chinese Academy of Agricultural Sciences, Chengdu National Agricultural Science & Technology Center, Chengdu 610213, China; wuzhi5416@163.com (Z.W.); zhaojin@caas.cn (J.Z.); linjunbin@caas.cn (J.L.); miaorenyun@caas.cn (R.M.); fengrencai@caas.cn (R.F.); 2 College of Food and Biological Engineering, Chengdu University, Chengdu 610106, China; 18285835857@163.com (S.Z.); chenmengxing12@163.com (M.C.); wudan616s@163.com (D.W.); wuyiyou519@163.com (Y.W.); lixiang@cdu.edu.cn (X.L.)

**Keywords:** *Oudemansiella raphanipes*, widely targeted metabolomics, antioxidant activity, enrichment approaches, network pharmacology

## Abstract

*Oudemansiella raphanipes* ethanolic extract (ORE) was prepared via ultrasonication-assisted ethanolic extraction and enriched through silica gel and macroporous adsorption resin chromatography to afford a non-/weakly polar fraction (ORE-S) and a polar fraction (ORE-N), respectively. This study aimed to (1) quantify major bioactive components (e.g., polyphenols, alkaloids, and terpenes) in ORE-S and ORE-N, (2) assess their antioxidant activities, (3) correlate compositional differences with antioxidant function, and (4) identify key antioxidant compounds along with their potential mechanisms of action. By integrating widely targeted metabolomics with network pharmacology, we not only elucidated how enrichment methods influence the antioxidant properties of ORE but also demonstrated the potential of ORE-N as a valuable source of bioactive compounds and natural antioxidants.

## 1. Introduction

Oxidative stress arises from an imbalance between reactive oxygen species (ROS) production and the body’s antioxidant defense. This imbalance is strongly linked to various diseases, including rheumatoid arthritis, atherosclerosis, chronic obstructive pulmonary disease, Alzheimer’s and cardiovascular diseases, metabolic disorders, and cancer [1,2]. Nowadays, factors such as sedentary lifestyle, consumption of processed food, and exposure to environmental toxins (i.e., heavy metals and other pollutants, ultraviolet and ionizing radiation) have substantially increased the prevalence of oxidative stress. Consequently, there is growing interest in exploring dietary antioxidants as a potential strategy to restore redox homeostasis, thereby preventing or mitigating these conditions.

Edible mushrooms are widely recognized as health-promoting food due to their rich nutrient content and low levels of calories, lipids, and cholesterol [3]. *Oudemansiella raphanipes* (Berk.) Pegler & T.W.K. Young (*O. raphanipes*), a traditional Chinese edible and medicinal mushroom, is renowned for its delicate texture, distinctive flavor, and rich bioactive composition. It is a valuable source of natural antioxidants including polyphenols, polysaccharides, and flavonoids [4]. Recent research has primarily focused on the polysaccharides from *O. raphanipes*, which exhibit diverse biological activities. These include antioxidant [5], anti-tumor [6], and anti-inflammatory effects [7]. Additionally, *O. raphanipes* polysaccharides have been shown to regulate gut microbiota [8,9], alleviate age-related intestinal epithelial barrier dysfunction [10], and provide hepato- [11,12], lung-, and reno-protective effects [13,14]. Despite these advances, studies on small-molecule bioactive compounds from *O. raphanipes* remain scarce. For instance, Guo et al. [15] identified the chemical composition of the petroleum ether extract from *O. raphanipes* and demonstrated its potential anti-cancer activity.

Food-grade ethanol is Generally Recognized As Safe (GRAS) by the U.S. Food and Drug Administration (FDA, 21 CFR §182.90) and approved by the European Directive 2009/32/EC for use in food extracts, flavorings, and other food applications [16,17]. It is widely used for extracting bioactive compounds from plants, edible mushrooms, and algae due to its non-toxic nature, environment-friendliness, and ability to dissolve both polar (e.g., flavonoids, polyphenols, and alkaloids) and moderately non-polar compounds (e.g., terpenes) [18,19]. By adjusting the ethanol/water ratio or combining it with other extraction methods (e.g., maceration, Soxhlet, ultrasound-assisted), ethanol can selectively target specific bioactive constituents while achieving high extraction yields. Marques et al. obtained functional lipid ingredients from algae via ultrasound-assisted ethanolic extraction, with potential applications in functional foods and nutraceuticals [20]. Ethanolic extracts from edible mushrooms have demonstrated significant health benefits [21]. For instance, ethanolic extracts from *Phellinus igniarius* and *Phellinus baumii* have been shown to induce cancer cell apoptosis via mitochondria-mediated pathways [22,23]. Given these advantages, ultrasonic-assisted ethanolic extraction was adopted in this study to prepare the *O. raphanipes* ethanolic extract (ORE).

**Figure 1:**
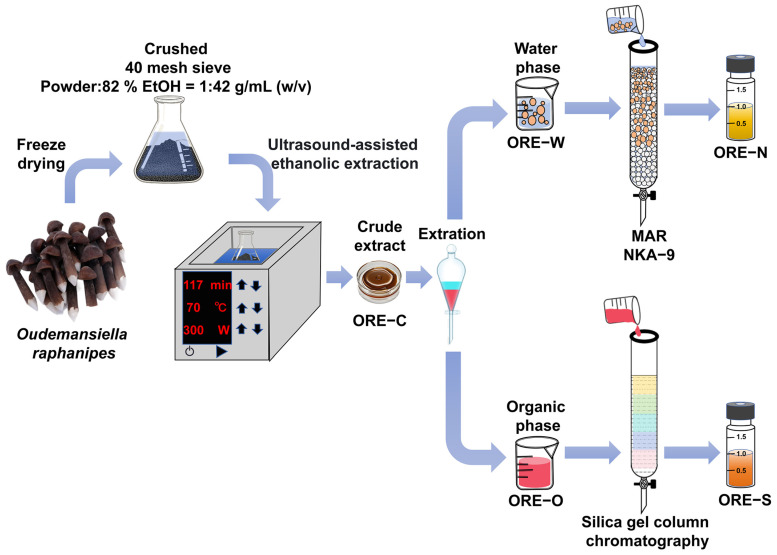
Schematic illustration of extraction and enrichment of bioactive compounds from *O. raphanipes*.

Macroporous adsorption resin (MAR) chromatography has emerged as an effective method for enriching bioactive compounds from mushroom ethanolic extracts, offering significant advantages over traditional techniques, including rapid and selective adsorption, high adsorption capacity, good mechanical strength, mild operation conditions, and easy scalability [24,25]. For instance, Wang et al. [26] enriched phenolic compounds from *Inonotus hispidus* using MAR HPD-600, achieving an 81.72% recovery rate and a 5-fold increase in purity. For the separation of non-polar and weakly polar compounds, silica gel chromatography remains one of the most widely employed methods [27]. In the current study, liquid–liquid extraction with petroleum ether and dichloromethane was conducted to separate non-polar/weakly polar compounds from polar components in ORE (Figure 1). The non-polar/weakly polar fraction was then further enriched through silica gel chromatography to obtain ORE-S, while the polar fraction was processed using MAR chromatography to yield ORE-N.

Given the chemical complexity of ethanolic extracts, widely targeted metabolomics serves as a powerful tool for comprehensive compositional analysis. This advanced approach integrates the strengths of targeted metabolomics (i.e., high specificity and sensitivity) and non-targeted metabolomics (i.e., comprehensive metabolite detection and new metabolite discovery), enabling the high-throughput detection of metabolites with wide coverage and high sensitivity [28]. For example, Wei et al. [29] utilized widely targeted metabolomics coupled with Kyoto Encyclopedia of Genes and Genomes (KEGG) analysis to evaluate how extraction methods alter the chemical profile of *Camellia oleifera* Abel. extract. Similarly, this technology identified fermentation-induced upregulation of polyphenolic compounds in *Elaeagnus angustifolia var. orientalis* (L.) Kuntze juice, particularly in the flavonoid biosynthetic pathway, which correlated with enhanced antioxidant activity [30]. Beyond compositional profiling, network pharmacology can further elucidate the functional implications of these metabolites by linking them to disease targets. This integrative strategy helps decipher the multi-target mechanisms underlying the pharmacological activities of bioactive compounds [31]. Therefore, the implementation of widely targeted metabolomics combined with network pharmacology to analyze ORE metabolites facilitates comprehension of the chemical basis of ORE’s health-promoting functions.

The chemical profile and bioactivity of ORE, particularly how enrichment strategies influence them, remain poorly understood. This study aimed to quantify major components (e.g., polyphenols, alkaloids, and terpenes) in the ORE-S and ORE-N, analyze their antioxidant activities, and correlate their composition with antioxidant function. Using widely targeted metabolomics, we revealed chemical differences driven by enrichment strategies. Multivariate statistical and KEGG pathway analyses identified differential metabolites and their mechanistic pathways, while network pharmacology pinpointed key antioxidant compounds and their potential mechanisms of action. Our findings not only establish an efficient method for extracting and enriching high-activity antioxidant compounds from *O. raphanipes* but also elucidate their chemical composition and mechanistic basis. This study highlights the potential of ORE-N as a valuable source of bioactive compounds and natural antioxidants.

## 2. Materials and Methods

### 2.1. Chemicals and Reagents

*O. raphanipes* was provided by the Institute of Urban Agriculture of Chinese Academy of Agricultural Sciences. It was identified as *Hymenopellis raphanipes* (also known as *O. raphanipes*) through ITS sequencing followed by BLAST comparison using the NCBI website (https://blast.ncbi.nlm.nih.gov/Blast.cgi, accessed on 20 June 2023) [32].

The following chemicals and reagents were sourced from reputable suppliers for various experimental purposes. Rutin (≥98%), ursolic acid (≥98%), vanillin (≥98%), NaCl (99%), meta-phosphoric acid (39%), ethanol, and fluorescein disodium salt (FL) (IND) were procured from Adamas (Shanghai, China). MARs, gallic acid (≥98%), bromothymol blue (≥98%), CH_3_COONa·3H_2_O (99%), D(+)-glucose (≥98%), coomassie brilliant blue G-250 (CBB G-250) (BS), 2,2-diphenyl-1-picrylhydrazyl (DPPH) (≥97%), and 2,2′-azino-bis(3-ethylbenzothiazoline-6-sulfonic acid) (ABTS) (≥98%) were obtained from Solarbio (Beijing, China). NaNO_2_ (99%), Al(NO_3_)_3_·9H_2_O (99%), NaOH (≥98%), Na_2_CO_3_ (≥98%), bromocresol green (≥95%), H_3_PO_4_ (85~95%), L(+)-ascorbic acid (>99%), KH_2_PO_4_ (≥99%), FeSO_4_·7H_2_O (≥99%), K_2_HPO_4_ (≥99%), NaH_2_PO_4_ (≥99%), and 2,2′-azobis(2-methylpropionamidine) dihydrochloride (AAPH) (97%) were purchased from Aladdin (Shanghai, China). Yuanye Bio-Technology Co., Ltd. (Shanghai, China) provided Folin–Clocalteu reagent (BR, 1 mol/L), berberine hydrochloride (≥98%), and bovine serum albumin (BSA) (98%). Jinshan Chemical Reagent Co., Ltd. (Chengdu, China) was the source of glacial acetic acid (GAA) (≥99.5%), dichloromethane (≥99.5%), petroleum ether (≥99%), ethyl acetate (≥99.5%), H_2_SO_4_ (95~98%), and HCl (36~38%). Chromatographic-grade acetonitrile (≥99.9%) and methanol (≥99.9%) were acquired from Merck (Darmstadt, Germany). K_2_S_2_O_8_ (99.5%) was purchased from Rhawn (Shanghai, China), while phenol (99%) was sourced from Macklin (Shanghai, China). Phygene (Fuzhou, China) supplied acetic acid buffer (300 mM, pH 3.6), TPTZ solution (10 mM), and FeCl_3_·6H_2_O solution (20 mM). Trolox (99%) was obtained from Sparkjade (Jinan, China). The water used in the experiments was ultra-pure (UP) water, prepared by an UP water machine (WP-UP-LH-20S, Water Purifier, Singapore).

### 2.2. Preparation of ORE-N and ORE-S

*O. raphanipes* fruiting bodies were dried using a vacuum freeze dryer (SCIENTZ-50F/A, Ningbo, China), crushed, and passed through a 40-mesh sieve. The obtained powder was mixed with 82% EtOH at a ratio of 1:42 g/mL (*w*/*v*), and then extracted using an ultrasonicator (SCIENTZ-SB25-12DTD, 300 W, 40 KHz) at 70 °C for 117 min. This extraction condition, which was optimized using response surface methodology (RSM) in our prior study, achieved a maximum yield of total flavonoids of 4.89 mg/g of dry powder in *O. raphanipes* [33]. Thereafter, the supernatant was filtered to obtain crude ORE (ORE-C). After ethanol removal by rotary evaporation, ORE-C was extracted with petroleum ether (3×) and dichloromethane (3×). The organic phases were combined, and the solvents were removed by high vacuum to obtain ORE-O. The water phase was freeze-dried to produce ORE-W. The ORE-O was passed through silica gel column chromatography with sequential elution with solvent A (petroleum ether/ethyl acetate, 5:1, *v*/*v*) and solvent B (dichloromethane: methanol, 9:1, *v*/*v*). The eluents containing flavonoids were identified using thin-layer chromatography (TLC) [34]. These eluents were combined, and the solvents were removed by high vacuum to obtain ORE-S. The active components in ORE-W were enriched by MAR NKA-9 to produce ORE-N. The resin selection and optimization of the enrichment process are described in Section 2.3.

### 2.3. Enrichment of Bioactive Components from ORE Using MAR Chromatography

#### 2.3.1. MAR Activation

The MARs were soaked in 95% EtOH overnight. After the removal of EtOH, the MARs were incubated with 5% HCl for 6 h and then rinsed to neutral with UP water. The same process was repeated after adding 5% NaOH. The activated MARs were reserved for later use. The properties of the MARs are presented in Table 1.

#### 2.3.2. Static Experiments

The optimal MAR for bioactive compound enrichment from ORE was selected according to the adsorption/desorption capacities of flavonoids on five MARs [35,36]. In brief, 0.3 g of each resin was added into ORE-W solution (0.43 mg/mL, 1 mL) and oscillated at 120 r/min and 25 °C for 24 h. The supernatant flavonoid concentration was determined according to Section 2.4, and the adsorption ratio was quantified by Equation (1). Subsequently, the MARs were rinsed extensively with UP water and incubated with the desorption solution (60% EtOH, 4 mL) under oscillation at 120 r/min and 25 °C for 24 h. The desorption and recovery ratios were determined based on the following formula:

(1) $$Adsorption\;capacity\;(mg/g)=\frac{\left(C_{0}-C_{1}\right)\times V_{1}}{M}$$

(2) $$Adsorption\;ratio\;(\%)=\frac{\left(C_{0}-C_{1}\right)}{C_{0}}\times 100$$

(3) $$Desorption\;capacity\;(mg/g)=\frac{C_{2}\times V_{2}}{M}$$

(4) $$Desorption\;ratio\;(\%)=\frac{C_{2}\times V_{2}}{\left(C_{0}-C_{1}\right)\times V_{1}}\times 100$$

(5) $$Recovery\;ratio\;(\%)=\frac{\left(C_{2}\times V_{2}\right)}{\left(C_{0}\times V_{1}\right)}\times 100$$

*C*_0_, *C*_1_, and *C*_2_ (mg/mL) represent the flavonoid concentrations in the initial, equilibrium, and desorption solutions, respectively, *V*_1_ and *V*_2_ (mL) represent the volumes of the initial sample and the desorption solutions, respectively, and *M* (g) represents the dry weight of the activated MARs.

The influence of ORE-W concentration on the adsorption effect of NKA-9 was carried out. In total, 0.3 g of the activated NKA-9 (dry weight) was incubated with 1 mL of ORE-W solution at different concentrations of flavonoids (0.2, 0.4, 0.6, 0.8, and 1.0 mg/mL) under oscillation (25 °C, 120 r/min) for 24 h. The flavonoid concentration of each equilibrium solution was determined to calculate the adsorption capacity using Equation (1).

The impact of adsorption time on the adsorption behavior of NKA-9 was evaluated according to a previous report [37]. In total, 1.5 g of the activated NKA-9 resin (dry weight) was incubated with the ORE-W solution (0.8 mg/mL, 3 mL) under oscillation at 25 °C and 120 r/min. At time points of 0.5, 1, 2, 5, 8, 10, and 24 h, the flavonoid concentration of the equilibrium solution was detected to calculate the adsorption rate and draw the adsorption kinetic curve.

The appropriate EtOH concentration was selected according to the method of Park et al. [38] with some modifications. In total, 0.3 g of NKA-9 resin was incubated with ORE-W solution (0.8 mg/mL, 1 mL) under oscillation at 25 °C and 120 r/min for 5 h. Thereafter, the resin was isolated and thoroughly washed with UP water to remove non-adsorbed impurities. The resin was dried and mixed with 2 mL of various EtOH concentrations (20, 30, 40,50, 60, 70, 80%). The desorption was conducted in a shaker (25 °C, 5 h, 120 r/min), and the flavonoid content in the desorption solution was quantified to calculate the desorption rate.

#### 2.3.3. Dynamic Experiments

Dynamic experiments were conducted using a column (200 mm × 15 mm i.d.) pre-packed with a certain amount of activated NKA-9. The bed volume (BV) of the resin was calculated to be 15 mL. The ORE-W solution at 0.8 mg/mL of flavonoids was loaded on the column at various flow rates (1, 2, 3, and 4 mL/min). Then, 5 mL aliquots of effluents were collected to determine the flavonoid concentration until the cumulative effluent volume reached 60 mL. The breakthrough curves were simulated by plotting the flavonoid concentration in the effluent versus the effluent volume [35]. The loading speed and amount could be determined based on the breakthrough curves.

After dynamic adsorption under optimal conditions was completed, the column was rinsed with water until the effluent was colorless. The column was eluted with 70% EtOH solution at a flow rate of 2 mL/min. Effluent aliquots (1 BV) were taken to determine the flavonoid concentration, and the elution curve was obtained by plotting the flavonoid concentration with the effluent volume [35]. The volume of 70% EtOH solution needed for flavonoid desorption could be assessed from the elution curve.

### 2.4. Determination of Major Bioactive Components in ORE Series Products

The content of flavonoids, polyphenols, alkaloids, terpenoids, total sugars, VC, and proteins in OREs (ORE-W, ORE-N, ORE-C, ORE-O, and ORE-S) was determined according to the following methods.

Total flavonoid content (TFC) was quantified based on a previously reported method [39]. In total, 200 μL of the sample solution was sequentially mixed with 50 μL of 5% (*w*/*v*) NaNO_2_ and 50 μL of 10% (*w*/*v*) Al(NO_3_)_3_ solution and allowed to stand for 5 min. Then, 200 μL of 4% (*w*/*v*) NaOH solution was added, mixed thoroughly, and incubated for 15 min. The absorbance at 510 nm was measured by a microplate reader (TECAN-Spark, Männedorf, Switzerland) (*n* = 3), and rutin (0~100 μg/mL) was used to plot the standard curve. The TFC was expressed as rutin equivalents (mg RE/g) based on the standard curve [40].

Total phenolic content (TPC) was determined using the Folin–Clocalteu method [41]. The sample solution (0.1 mL) was mixed sequentially with 0.5 mL of the Folin–Clocalteu reagent and 0.4 mL of Na_2_CO_3_ (7.5%) and allowed to stand for 1 h. The absorbance at 765 nm was measured by a microplate reader (*n* = 3), and the standard curve was plotted using gallic acid (0~70 μg/mL). The TPC was expressed in gallic acid equivalents (mg GAE/g) based on the standard curve.

Total terpenoid content (TTC) was determined by referring to the method of Xie et al. [42]. In total, 0.4 mL of the sample solution was placed in a 10 mL test tube to dry by evaporation. Then, 0.1 mL of freshly prepared 5% (*w*/*w*) vanillin–glacial acetic acid solution and 0.8 mL of concentrated sulfuric acid were added and allowed to react at 60 °C for 15 min. After cooling in an ice bath for 5 min, 5 mL of GAA was added, mixed thoroughly, and incubated for 15 min. The absorbance at 550 nm was measured (*n* = 3), and the standard curve was plotted using ursolic acid (0~100 μg). The TTC was expressed in ursolic acid equivalents (mg UAE/g) based on the standard curve.

Total alkaloid content (TAC) was determined using the acid dye colorimetry method with some modifications [43]. In total, 3.5 mL of acetate-sodium acetate buffer (0.2 mol/L, pH 5.4) and 3 mL of bromothymol blue solution (0.5 mg/mL) were added in 0.5 mL of the sample solution. The mixture was extracted with 7.5 mL of dichloromethane. The dichloromethane layer was taken to measure the absorbance at 410 nm using a UV-Visible spectrophotometer (GENESYS 50, Thermo Scientific, Waltham, MA, USA) (*n* = 3). The standard curve was plotted using berberine hydrochloride (0~800 μg/mL) and the TAC was expressed in berberine hydrochloride equivalents (mg BHE/g) based on the standard curve.

Total sugar content was quantified using the phenol–sulfuric acid method [44]. In total, 0.1 mL of the sample solution was mixed evenly with 0.1 mL of 5% phenol (*w*/*v*) and 0.5 mL of sulfuric acid. After 20 min, the absorbance at 490 nm was measured by a microplate reader (*n* = 3). The standard curve was plotted using D(+)-glucose (0~100 μg/mL), and the total sugar content was calculated based on the standard curve.

Protein content was quantified using the Bradford method [45]. The Bradford reagent was prepared using CBB G-250. In total, 0.1 mL of the sample solution was thoroughly mixed with 0.5 mL of the Bradford reagent to react for 10 min. The absorbance at 595 nm was measured by a microplate reader (*n* = 3). The standard curve was plotted using BSA (0~100 μg/mL), and the protein content was calculated based on the standard curve.

The content of VC was determined by a high-performance liquid chromatograph (LC-2030 Plus, SHIMADZU, Kyoto, Japan) using SHIMADZU Shim-pack GIST C18 column (5 μm, 4.6 × 150 mm) at 25 °C. Isocratic elution was carried out using the mobile phase starting from 90% solvent A (0.05 mol/L KH_2_PO_4_, pH 3.0) and 10% solvent B (methanol) with a flow rate of 0.7 mL/min. The absorbance was monitored at 245 nm (*n* = 3), and the standard curve was obtained by plotting the concentration of L(+)-ascorbic acid (0~50 μg/mL) with the peak area.

### 2.5. Antioxidant Activities of ORE Series Products

#### 2.5.1. ABTS Radical Scavenging ABILITY

The ABTS assay was performed according to the reported method with slight modifications [46]. The ABTS solution (7.4 mM) and the K_2_S_2_O_8_ solution (2.6 mM) were mixed at a ratio of 1:1 (*v*/*v*), followed by incubation in the dark for 12–16 h to obtain the ABTS^+^· radicals. This solution was diluted with absolute ethanol and its absorbance at 734 nm was adjusted to 0.4 ± 0.02. Thereafter, 150 μL of ABTS^+^· solution was added to 150 μL of the sample solution. After incubation at RT for 6 min, the absorbance at 734 nm was measured by a microplate reader. The ABTS radical scavenging ability was calculated according to Equation (6) using VC as the positive control:

(6) $$ABTS\;radical\;scavenging\;activity\;(\%)=\left(1-\frac{A_{1}-A_{2}}{A_{0}}\right)\times 100$$

*A*_0_, *A*_1_, and *A*_2_ represent the absorbances at 734 nm of the ABTS^+^·solution, sample solution, and 70% EtOH solution, respectively.

#### 2.5.2. DPPH Radical Scavenging Ability

The DPPH assay was performed according to the reported method with slight modifications [47]. The absorbance at 517 nm of the DPPH ethanol solution was adjusted to 0.165 ± 0.02. Then, 150 μL of the DPPH solution was added to 150 μL of the sample solution. After incubation at RT for 30 min, the absorbance at 517 nm was measured by a microplate reader. The DPPH radical scavenging activity was calculated according to Equation (7) using VC as the positive control:

(7) $$DPPH\;radical\;scavenging\;activity\;(\%)=\left(1-\frac{A_{1}-A_{2}}{A_{0}}\right)\times 100$$

*A*_0_, *A*_1_, and *A*_2_ represent the absorbances at 517 nm of the DPPH solution, sample solution, and 70% EtOH solution, respectively.

#### 2.5.3. Ferric Ion Reducing Antioxidant Power (FRAP)

FRAP analysis was carried out using the reported method with slight modifications [48]. The FRAP solution was prepared by mixing acetic acid buffer (300 mM, pH 3.6), TPTZ solution (10 mM), and FeCl_3_·6H_2_O (20 mM) at a ratio of 10:1:1 (*v*/*v*/*v*). Then, 40 μL of the sample or FeSO_4_ solution was added to 200 μL of the FRAP solution. After incubation at 37 °C for 10 min, the absorbance at 593 nm was measured by a microplate reader. FeSO_4_ was utilized as the standard, and the FRAP values were determined based on the standard curve (y = 2.210x − 0.007783 (0–1 mM, R^2^ = 0.9985)). The results were expressed in milimoles of ferrous sulfate equivalents.

#### 2.5.4. Oxygen Radical Absorbance Capacity (ORAC)

ORAC assay was conducted based on the reported methods with slight modifications [49]. Different concentrations of Trolox solutions (3.125–100 μM) were prepared using PBS buffer (75 mM, pH 7.4). Then, 20 μL of Trolox or the sample solution was added in the FL solution (160 μL, 200 nM) under shaking for 2 min. After incubation at 37 °C for 30 min, the AAPH solution (20 μL, 153 mM) was rapidly added. The mixture was excited at 485 nm, and the emission was collected at 538 nm at 37 °C every 2 min until the fluorescence decayed to the baseline. The Trolox standard curve was established with the relative fluorescence intensity as the ordinate and the Trolox concentration as the abscissa. The ORAC values were calculated based on the standard curve (y = 0.5808x + 3.622 (R^2^ = 0.9908)) and expressed in micromoles of Trolox equivalents.

### 2.6. Widely Targeted Metabolomic Analysis

#### 2.6.1. Sample Preparation

ORE-S (30 mg/mL) and ORE-N (30 mg/mL) were redissolved in ethyl acetate/methanol (1:2, *v*/*v*) and 70% methanol solutions containing the internal standards, respectively. The mixtures were vortexed at room temperature (RT) for 15 min and kept at −20 °C for 30 min. They were centrifuged at 12,000 r/min for 3 min at 4 °C, and the supernatants were filtered through 0.22 μm PTFE syringe filters prior to UPLC-MS/MS analysis. Quality control (QC) samples were obtained by mixing aliquots of each sample to monitor systematic stability and reproducibility. It should be noted that chemical profiling of ORE-N and ORE-S was performed, but no in vivo or in vitro biological validation was conducted.

#### 2.6.2. Chromatography and Mass Spectrometry Conditions

Metabolomic analysis was conducted using an ExionL™ AD UPLC system coupled with tandem mass spectrometry (UPLC-MS/MS). In total, 2 μL of the sample was separated by an Agilent SB-C18 column (1.8 μm, 2.1 mm × 100 mm) with the mobile phase consisting of 0.1% formic acid in water (A) and 0.1% formic acid in acetonitrile (B) at a flow rate of 0.35 mL/min. The gradient was linearly increased from 5% to 95% B over 9 min, held at 95% for 1 min, stepped back down to 5% in 1 min, and finally balanced at 5% for 3 min.

For the MS analysis, electrospray ionization was conducted at 500 °C, and the ion spray voltage was 5500 V and −4500 V for positive and negative ion modes, respectively. The ion source gas I, gas II, and curtain gas were set at 50, 60, and 25 psi, respectively, and the collision-induced ionization parameter was set to high. Triple quadrupole (QQQ) scanning used the MRM mode with medium collision gas (nitrogen).

#### 2.6.3. Qualitative and Quantitative Analysis of Metabolites

The qualitative and quantitative analysis of metabolites was carried out using the method of Tang et al. [50]. Based on the MetWare database (MWDB, Metware Biotechnology Co., Ltd., Wuhan, China), metabolites were identified by secondary spectrum information that excluded the isotopic and duplicate (i.e., Na^+^, K^+^, and NH_4_^+^ adducts, fragment ions of other larger molecular weight compounds) signals. Metabolite quantification was accomplished by the multiple reaction monitoring (MRM) analysis of QQQ mass spectrometry. The mass spectral peaks of all metabolites were integrated, while the mass spectral peaks of the same metabolite in different samples were corrected [51].

### 2.7. Network Pharmacology Analysis

To elucidate the molecular mechanisms of ORE-N’s antioxidant activity, a network pharmacology approach was adopted. Forty-nine compounds (the top twenty flavonoids, phenolic acids, and alkaloids by relative abundance in ORE-N) were selected based on metabolomics data. The SMILES structures of these compounds were retrieved from PubChem (https://pubchem.ncbi.nlm.nih.gov, accessed on 15 March 2025) and imported into Swiss Target Prediction (http://swisstargetprediction.ch, accessed on 15 March 2025) to predict human targets (probability > 0). Antioxidant-related genes were obtained from GeneCards (https://www.genecards.org/, accessed on 15 March 2025) using the keyword “anti-oxidant”. Overlapping targets between the ORE-N compounds and antioxidant genes were identified using Venny 2.1.0 (https://bioinfogp.cnb.csic.es/tools/venny/, accessed on 15 March 2025). Protein interactions were analyzed via STRING (https://cn.string-db.org/, accessed on 15 March 2025) with a confidence score of 0.4; the results were visualized using Cytoscape 3.10.3. Core targets were filtered based on degree centrality (≥median). Gene Ontology (GO) and KEGG pathway analyses were performed using DAVID (https://davidbioinformatics.nih.gov/, accessed on 15 March 2025) with a significance level of *p* < 0.05.

### 2.8. Data Processing and Multivariate Statistical Analysis

All experiments were repeated three times, and the results were expressed as mean ± standard deviation (SD). All data were analyzed using IBM SPSS Statistics 26 software. One-way analysis of variance (ANOVA) and Duncan’s post hoc test were used to determine the significance of differences between samples. A *p*-value of <0.05 was accepted as statistically significant. Principal component analysis (PCA) and orthogonal partial least squares discriminant analysis (OPLS-DA) were performed using R 4.2.1 software. Pearson correlation, volcano plots, and heatmaps were plotted using R 4.2.1 software. The half maximum inhibitory concentration (IC_50_) and the area under the curve were obtained using GraphPad Prism 9.5 software (San Diego, CA, USA). Metabolites were annotated using the KEGG database [52], and only metabolic pathways containing differential metabolites were presented.

## 3. Results and Discussion

### 3.1. Enrichment of Bioactive Components from ORE

**Figure 2:**
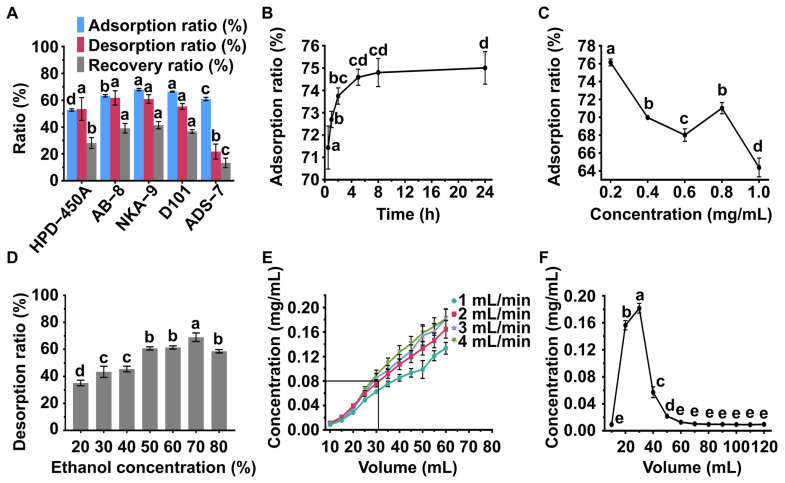
(**A**) Adsorption capacity, desorption ratio, and recovery of flavonoids with different MARs. (**B**) Adsorption kinetic curve of flavonoids on MAR NKA-9. (**C**) Effect of initial concentration on adsorption capacity of MAR NKA-9. (**D**) Effect of ethanol concentration on desorption ratio of MAR NKA-9. (**E**) Dynamic breakthrough curves. (**F**) Dynamic desorption curve. Different small letters indicate significant differences (*p* < 0.05, *n* = 3).

ORE-C was prepared via ultrasound-assisted ethanolic extraction, followed by two distinct enrichment methods to afford ORE-S and ORE-N (Figure 1). The MAR enrichment conditions were optimized based on flavonoid content. Among five tested resins (Table 1 and Figure 2A), D101 and NKA-9 exhibited superior adsorption/desorption capacities and recover rates (*p* < 0.05), with NKA-9 showing slightly better performance, though the difference was not statistically significant. Adsorption mechanisms primarily involved hydrogen bonding and van der Waals interactions [24]. D101 favors non-polar compounds, whereas NKA-9 preferentially adsorbs polar substances [53]. Given that most polyphenols with high antioxidant activity are polar, NKA-9 was chosen for subsequent experiments. This choice was based on the predominant enrichment of target flavonoids in the hydrophilic fraction (ORE-W) following petroleum ether/dichloromethane partitioning (ORE-O). Flavonoid adsorption kinetics on NKA-9 revealed rapid adsorption within 2 h, reaching near-equilibrium by 5 h, which was thus set as in the subsequent studies. The loading concentration significantly influenced flavonoid adsorption by modulating the degree of contact between the flavonoids and resin [54]. As shown in Figure 2C, the highest adsorption rate occurred at 0.2 mg/mL, but a loading concentration of 0.8 mg/mL was selected as the optimum balance between adsorption efficiency and time cost. Desorption was driven by competitive intermolecular interactions between adsorbed flavonoids, the resin, and solvent molecules [55]. The impact of EtOH concentration on flavonoid desorption from NKA-9 is depicted in Figure 2D. The desorption rate increased from 20 to 70% EtOH but declined at higher concentrations. The maximum desorption capacity (68.94%) was achieved with 70% EtOH, which was thus chosen as the optimal desorption solvent. Dynamic adsorption conditions were further optimized by loading flavonoid solution (0.8 mg/mL) at varying flow rates. Breakthrough curves were generated, with the breakthrough point defined as the effluent flavonoid concentration reaching 10% of the inlet loading [56]. Figure 2E showed that higher flow rates led to earlier breakthrough points, likely due to reduced flavonoid–resin interaction time. A flow rate of 2.0 mL/min and a loading volume of 30 mL were selected for optimal adsorption efficiency. Finally, flavonoids were eluted with 70% EtOH at 2.0 mL/min. Figure 2F indicates that flavonoids were almost entirely eluted by 60 mL of eluent, so this eluent volume was selected to minimize EtOH waste. In summary, the optimized conditions for obtaining ORE-N were as follows: loading 30 mL of ORE-W (0.8 mg/mL of flavonoids) onto NKA-9 at 2 mL/min for 5 h of adsorption, followed by elution with 60 mL of 70% EtOH at 2 mL/min.

### 3.2. Comparative Analysis of Major Bioactive Components in ORE Series Products Using Different Enrichment Methods

**Figure 3:**
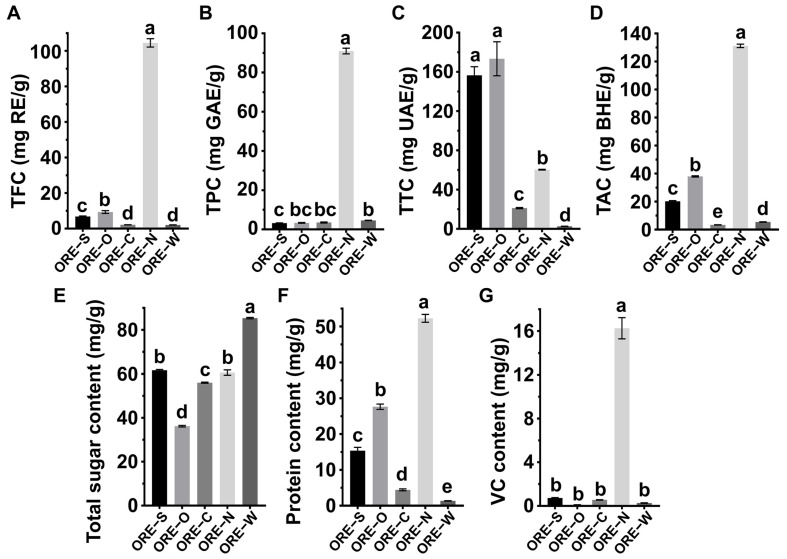
Content of major bioactive components in OREs under different processing methods: flavonoids (**A**), polyphenols (**B**), terpenoids (**C**), alkaloids (**D**), total sugars (**E**), proteins (**F**), and VC (**G**). Different small letters indicate significant differences (*p* < 0.05, *n* = 3).

The contents of major bioactive components (i.e., flavonoids, polyphenols, terpenoids, alkaloids, total sugars, proteins, and VC) in ORE was determined by standard methods (*n* = 3) (Figure 3). All data were expressed as mg/g dry weight. Compared with other groups, ORE-N possessed significantly higher levels of flavonoids, polyphenols, alkaloids, proteins, and VC (*p* < 0.05). Polyphenols including flavonoids are increasingly recognized for their health-promoting properties [57]. Notably, ORE-N contained 104.52 mg RE/g of TFC and 90.96 mg GAE/g of TPC, representing 49- and 27-fold increases, respectively, over ORE-C. Alkaloids, nitrogen-containing bioactive compounds, contribute to multiple biological activities and chronic disease prevention [58]. ORE-N showed 38-fold higher TAC (131.16 mg BE/g) than ORE-C, further confirming NKA-9 resin’s efficacy in enriching these compounds, which was consistent with previous findings [56]. Additionally, ORE-N contained 52.28 mg/g of proteins and 16.26 mg/g of VC, 12- and 30-fold higher than ORE-C, respectively. These proteins probably belonged to alcohol-soluble proteins, since the extraction proceeded in 82% EtOH. In contrast, ORE-O and ORE-S displayed significantly higher TTC (173.35 and 156.49 BHE/g, respectively) than other groups, suggesting that the terpenoids in ORE were non-polar or weak polar—corroborating previous findings on their prevalence in the essential oils of edible species [59]. Terpenoids are known for their anti-hyperglycemic, anxiolytic, anti-inflammatory, and antioxidant effects [59]. All OREs had low sugar levels, indicating effective removal of macromolecular polysaccharides during processing. In summary, enrichment methods significantly influenced ORE’s nutrient profile, likely explaining variations in their functional properties.

### 3.3. Analysis of Antioxidant Activity of ORE Series Products

**Figure 4:**
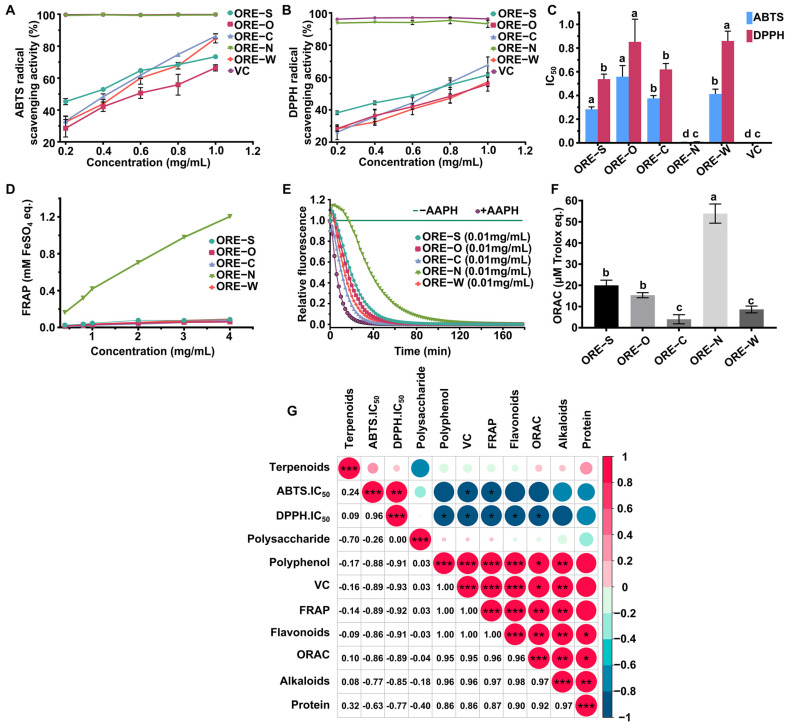
(**A**–**F**) In vitro analysis of antioxidant activities of OREs with different processing methods. Different small letters indicate significant differences (*p* < 0.05, *n* = 3). (**G**) Correlation analysis between nutritional components and antioxidant activities. (* *p* < 0.05; ** *p* < 0.01; *** *p* < 0.001).

Since antioxidants often act through different mechanisms—including electron transfer (ET) and hydrogen atom transfer (HAT)—relying on a single assay may provide an incomplete assessment of their overall activity. To comprehensively evaluate the in vitro antioxidant capacity of OREs, we employed complementary assays targeting these distinct mechanisms: ET-based (ABTS, FRAP) and HAT-based (DPPH, ORAC) [60]. All OREs exhibited concentration-dependent scavenging activities against ABTS and DPPH radicals (Figure 4A,B), with ORE-N demonstrating significantly higher scavenging rates than other OREs (*p* < 0.05). The IC_50_ values of ORE-N for ABTS (4.81 μg/mL) and DPPH (9.34 μg/mL) were 78- and 66-fold lower than those of ORE-C, respectively. Notably, these values approached the potency of VC (3.00 and 1.28 μg/mL) (Figure 4C), underscoring ORE-N’s strong antioxidant potential. The FRAP assay evaluates antioxidant activity based on the reduction of Fe^3+^ to Fe^2+^. In biological systems, Fe^3+^ participates in key oxidative pathways, particularly the Fenton reaction, producing highly reactive hydroxyl radicals that cause cellular oxidative damage [61]. As shown in Figure 4D, ORE-N displayed dose-dependent FRAP activity, whereas other OREs had negligible Fe^3+^ reduction at all the concentrations. At 4 mg/mL, ORE-N reached a FRAP value of 1.2 mM FeSO_4_, consistent with previous findings [62]. In the ORAC assay, OREs exhibited exceptional peroxyl radical scavenging capacity (5.4 mmol TE g^−1^), surpassing ORE-S and ORE-C by 2.7- and 13.5-fold, respectively (Figure 4E,F). This value also exceeded those of most foods listed in the USDA database [63], highlighting its superior antioxidant efficacy. Collectively, these results demonstrated that ORE-N possessed remarkable antioxidant activity, likely attributable to the high concentrations of flavonoids, polyphenols, alkaloids, and VC in ORE-N.

### 3.4. The Relationship Between Bioactive Components and Antioxidant Properties of ORE-N and ORE-S

We assumed that the marked differences in antioxidant capacities of ORE-N and ORE-S were linked to their divergent chemical compositions. To test this, Pearson correlation analysis was conducted to assess the relationships between antioxidant activity and chemical composition. As shown in Figure 4G, polyphenols, flavonoids, alkaloids, and VC exhibited strong negative correlations with ABTS and DPPH IC_50_ values (R^2^ > 0.7). Meanwhile, their contents were strongly and positively associated with ORAC and FRAP antioxidant activities (R^2^ ≥ 0.95). These findings align with prior studies demonstrating that polyphenols, flavonoids, alkaloids, and VC are key contributors to antioxidant activity, often acting synergistically to combat oxidative stress [64,65,66,67]. In contrast, polysaccharides and terpenoids exhibited poor correlations with all antioxidant indicators, consistent with earlier reports [68]. In addition, protein levels highly positively correlated with polyphenols, flavonoids, alkaloids, and VC content, suggesting potential synergistic interactions that amplify antioxidant activities. Previous studies also reported that the combination of antioxidants and proteins played a synergistic role in the overall antioxidant activities [69,70]. Given that ORE-N contained significantly higher content of polyphenols, flavonoids, VC, and alkaloids than other OREs, this might be the main reason for its superior antioxidant activity. Thus, ORE-N’s high nutritional value and potent antioxidant properties make it a promising candidate for functional foods, nutraceuticals, and pharmaceutical applications.

### 3.5. Metabolites in ORE Series Products Identified by UPLC–MS/MS

**Figure 5:**
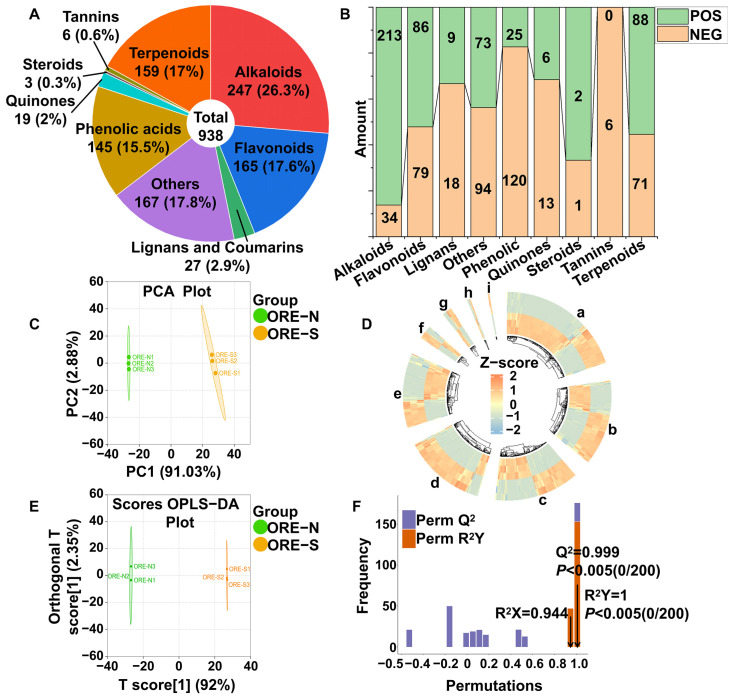
(**A**) Classification of total 938 compounds identified in ORE-N and ORE-S. (**B**) Compound composition in positive and negative ion modes. (**C**) PCA score plot. (**D**) Circular hierarchical cluster heatmap of all identified compounds (a–i represent alkaloids, others, flavonoids, terpenoids, phenolic acids, lignans and coumarins, quinones, tannins, and steroids, respectively). (**E**) OPLS-DA score plot and (**F**) 200 X permutation tests.

The chemical compositions of ORE-N and ORE-S enriched from ORE-C through two different methods were systematically examined by widely targeted metabolomics based on UPLC-MS/MS. QC samples demonstrated excellent reproducibility, as evidenced by highly overlapping total ion chromatograms (TICs) in retention time and peak intensity (Figure S1). As shown in Figure 5A,B, a total of 938 compounds was identified, including 247 alkaloids (i.e., 119 alkaloids, 43 plumerane, 16 pyridine alkaloids, 16 pyrrole alkaloids, 15 phenolamine, 14 quinoline alkaloids, 13 piperidine alkaloids, 4 isoquinoline alkaloids, 2 aporphine alkaloids, 2 quinorisidine alkaloids, 2 tropan alkaloids, and 1 benzylphenylethylamine alkaoids), 165 flavonoids (i.e., 61 flavones, 35 flavonols, 7 isoflavones, 3 flavanonols, 18 chalcones, 6 flavanols, 20 flavanones, 1 dihydroisoflavones, and 14 other flavonoids), 159 terpenoids (i.e., 54 sesquiterpenoids, 54 triterpene, 25 ditepenoids, 19 monoterpenoids, 6 terpene, and 1 triterpene saponin), 19 quinones (i.e., 10 quinones, 8 anthraquinone, and 1 naphthol), 145 phenolic acids, 10 lignans, 17 coumarins, 3 steroids, 6 tannins, and 167 other compounds (e.g., aldehyde, alcohol, ketone, etc.). The comprehensive metabolite profiling (Table S1) revealed numerous bioactive compounds with diverse health benefits, including antioxidant, anti-inflammatory, antibacterial, and anti-cancer effects. Among these, the flavonoid 2′-hydroxydaidzein exhibited potent anti-inflammatory and antioxidant activities through inhibiting β-glucuronidase and lysozyme release from rat neutrophils [71]. Similarly, the flavonoid tangeretin possessed anti-inflammatory, antioxidant, and anti-tumor effects [72]. The quinoline alkaloid echinopsine, isolated from *Echinops sphaerocephalus* L. of the Compositae family, showed a wide range of biological activities, including antiviral, insecticidal, and bactericidal activities [73]. Phenolic acids such as cinnamic acid demonstrated lipid-lowering, anti-obesity, anti-hyperglycemic, cardioprotective, and vasodilatory activities in vivo and in vitro [74]. Among the anthraquinones, chrysophanol-9-anthrone stood out for its unique anti-tumor mechanism involving both anti-angiogenesis and immune system stimulation [75]. The sesquiterpene britannilactone, derived from *Inula britannica*, presented multiple biological activities encompassing anti-inflammatory, anti-tumor, and anti-pigmentation [76].

### 3.6. Overview of Metabolomic Differences Between ORE-N and ORE-S

After unit variance normalization of the metabolomic data, we performed unsupervised PCA to preliminarily comprehend the compositional differences between ORE-N and ORE-S. As shown in Figure 5C, samples within each group aggregated closely, suggesting high data reliability and reproducibility. Notably, PCA revealed clear separation between ORE-N and ORE-S, with the two principal components (PC1 and PC2) accounting for 91.03% and 2.88% of the total variance, respectively. The substantial compositional differences between ORE-N and ORE-S highlighted the critical impact of enrichment methodology on the final product’s chemical profile.

Hierarchical cluster analysis (HCA) was also conducted on all metabolites, visualizing the results as a circular heatmap with dendrogram using the R package ComplexHeatmap (4.4.1). The heatmap displayed unit variance-scaled metabolite intensities, revealing distinct clustering patterns between ORE-N and ORE-S that corroborated the PCA results (Figure 5D). High intra-group consistency (evidenced by uniform color blocks) confirmed excellent data reliability and repeatability. These differential clustering patterns suggested that ORE-N and ORE-S metabolites might participate in distinct biological processes or exhibit divergent responses to specific conditions, providing a robust basis for subsequent differential metabolite screening and KEGG analysis. Complementary to these analyses, we calculated Pearson’s correlation coefficients (PCCs) between samples using the cor function in R (Figure S2). The correlation heatmap revealed strong intra-group correlations (high PCC values), confirming biological reproducibility, while significantly lower inter-group correlations underscored the fundamental compositional divergence between ORE-N and ORE-S.

Supervised OPLS-DA was further utilized to eliminate irrelevant variables and screen for major differential compounds between ORE-N and ORE-S [77]. OPLS-DA was performed on the raw data after log_2_ transformation on the data and mean centering. The OPLS-DA score plot demonstrated clear separation between ORE-N and ORE-S (Figure 5E), corroborating the PCA results and confirming significant compositional differences. R^2^X and R^2^Y represented the explanatory power of OPLS-DA to the proportion of variance in the X and Y matrices, respectively, while Q^2^ suggested its predictive capacity (Q^2^ > 0.9, excellent) [78]. As shown in Figure 5F, the R^2^X, R^2^Y, and Q^2^ values were determined to be 0.944, 1, and 0.999, respectively, illustrating that the model was stable and reliable. In addition, both *p*-values of R^2^Y and Q^2^ were less than 0.05 in the 200 permutation tests, confirming that the model did not overfit and could be used for the subsequent screening of differential compounds based on the variable importance in projection (VIP) values [29].

### 3.7. Selection of Differential Metabolites Between ORE-N and ORE-S and Their Enrichment in KEGG Pathways

**Figure 6:**
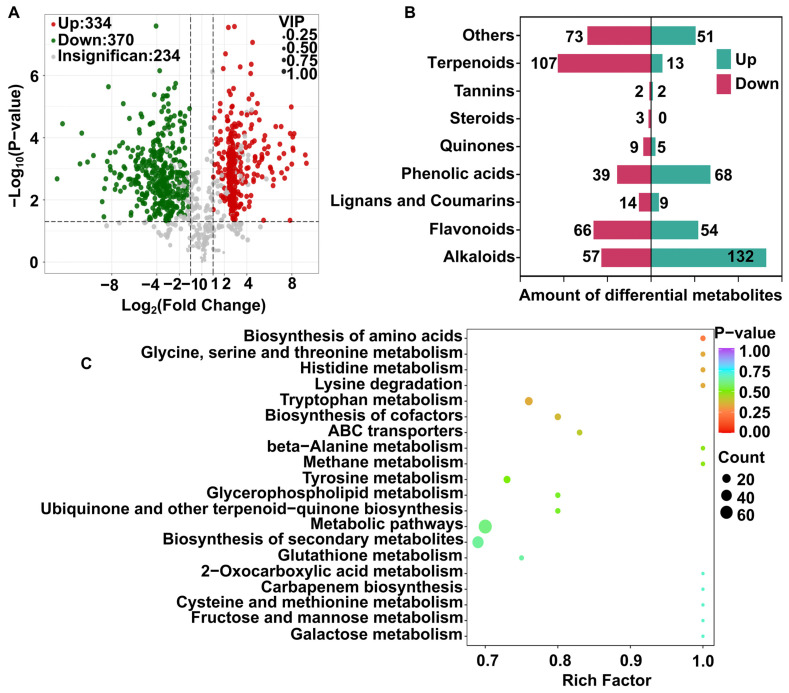
(**A**) A volcano plot of the differentially abundant metabolites in OREs with different enrichment methods (ORE-N vs. ORE-S). (**B**) Classification of the differentially abundant metabolites in OREs with different enrichment methods (ORE-N vs. ORE-S). (**C**) KEGG annotations and enrichment results of the differential metabolites.

Differential compounds between ORE-N and ORE-S were screened out based on the OPLS-DA results using a threshold of VIP > 1, |Log_2_FC| ≥ 1.0, and −log_10_(*p*-value) > 1.30 (Table S2). Substantial difference in compound composition between ORE-N and ORE-S was visualized by a volcano plot (Figure 6A). In total, 704 differential compounds were annotated, with 334 upregulated and 370 downregulated in ORE-N compared to ORE-S. Among these compounds, there were 189 alkaloids (132 upregulated, 57 downregulated), 120 flavonoids (54 upregulated, 66 downregulated), 107 phenolic acids (68 upregulated, 39 downregulated), 120 terpenoids (13 upregulated, 107 downregulated), 23 lignans and coumarins (9 upregulated, 14 downregulated), 14 quinones (5 upregulated, 9 downregulated), 4 tannins (2 upregulated, 2 downregulated), and 124 other metabolites (51 upregulated, 73 downregulated) (Figure 6B). The predominant differential metabolites were alkaloids, flavonoids, phenolic acids, and terpenoids, which displayed a broad spectrum of biological activities and contributed benefits to human health [79].

**Figure 7:**
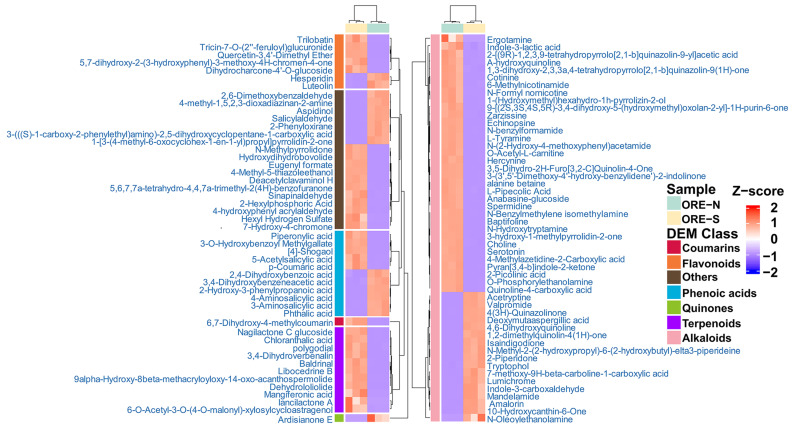
HCA heatmap of TOP100 differential metabolites in OREs with different enrichment methods (ORE-N vs. ORE-S).

The identities and relative abundances of the top 100 different compounds (ranked by |Log_2_FC|) were visualized in a heatmap (Figure 7). Alkaloids represented the most abundant class among these metabolites. For instance, the upregulated alkaloid 5-hydroxytryptamine is an important neurotransmitter involved in regulating mood, sleep, appetite, learning, memory, body temperature, and pain response [80,81]. Another notable alkaloid, echinopsine, possessed bactericidal, anti-tumor, and anti-fungal effects [73]. The bioamine spermidine showed broad pharmacological effects, including anti-inflammatory, anti-tumor, anti-aging, and cardiovascular protection [82,83]. Recent studies indicated that spermidine delayed cellular senescence by suppressing oxidative stress, reducing ROS levels, and activating SIRT3-dependent pathways [84]. The downregulated alkaloid lumichrome, a riboflavin photodegradation product, was shown to inhibit lung cancer cell growth through p53-dependent apoptosis [85]. The heatmap also highlighted significant changes in phenolic acids, such as 2-hydroxy-3-phenylpropanoic acid, a potent antimicrobial agent that disrupts bacterial and fungal ROS metabolism [86]. 4-Aminosalicylic acid, an antibiotic used in tuberculosis treatment [87], exhibited antioxidant effects and alleviated colon damage by enhancing superoxide dismutase (SOD) activity and reducing malondialdehyde (MDA) levels in colitis models [88]. 4-Shogaol, an active constituent of ginger, effectively suppressed breast cancer metastasis by inhibiting nuclear factor κB (NF-κB) activation [89]. Among the differential flavonoids, luteolin emerged as a multifunctional bioactive compound, enhancing antioxidant activity via Nrf2/MAPK signaling while also exerting anti-inflammatory, anti-cancer, anti-diabetic, anti-cardiovascular, and neuroprotective effects [90,91]. Hesperidin, a flavanone glycoside abundant in citrus fruits, mitigated oxidative stress and inflammation [92], with additional benefits in diabetes management [93]. Mechanistic studies revealed that hesperidin downregulated the expression of peroxisome proliferator-activated receptor and Bcl-2 in a mouse model, thereby attenuating inflammation and antioxidant stress following acute myocardial infarction [94]. Similarly, trilobatin, a member of the dihydrochalcone family, exhibited robust antioxidant, anti-inflammatory, and anti-diabetic properties [95]. Its protective effects against LPS-induced acute lung injury were mediated through the activation of the AMPK/GSK3β-Nrf2 pathway and the suppression of NF-κB signaling [96]. Among the differential compounds, only one coumarin was identified: 6,7-Dihydroxy-4-methylcoumarin. It displayed potent anti-inflammatory effects in RAW 264.7 macrophages by downregulating inflammatory mediators (e.g., nitric oxide and prostaglandin E2) and suppressing pro-inflammatory cytokine release (e.g., IL-1β and IL-6) [97].

To elucidate the physiological roles of the differential metabolites, pathway enrichment analysis was performed using the KEGG database. As illustrated in the bubble chart (Figure 6C), the differential metabolites primarily engaged in 20 pathways ranked by ascending *p*-value. The top five pathways included amino acid biosynthesis, glycine, serine and threonine metabolism, histidine metabolism, lysine degradation, and tryptophan metabolism, indicating a strong predominance of amino acid-related metabolic and degradative pathways. Notably, the tryptophan metabolism pathway exhibited the most pronounced enrichment. Within this pathway, key metabolites such as serotonin (5-hydroxytryptamine) contribute to antioxidant defense through direct free radical scavenging and modulation of antioxidant enzyme activities (e.g., SOD and CAT) via the Nrf2/ARE signaling axis [98]. These findings indicated that amino acid metabolism in *O. raphanipes* contributed substantially to the biosynthesis of antioxidant-active components.

### 3.8. Network Pharmacology-Based Elucidation of Antioxidant Mechanisms

**Figure 8:**
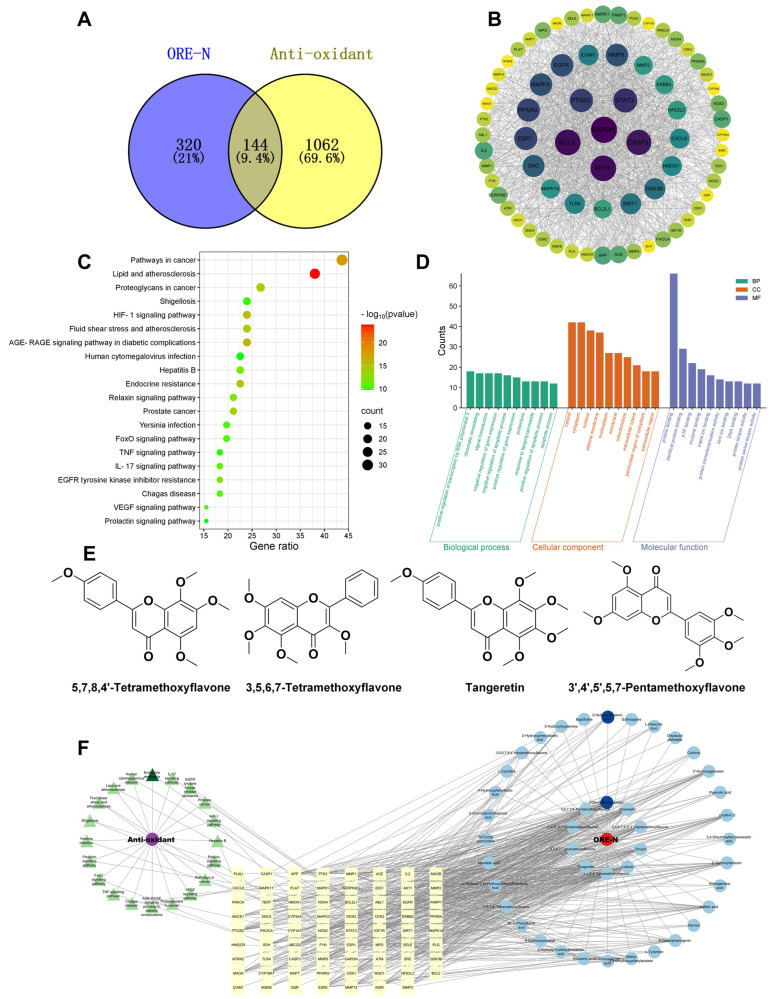
(**A**) Venn diagram of overlapping targets between ORE-N and oxidative damage. (**B**) PPI network of key targets. (**C**) KEGG pathway enrichment analysis of top 20 pathways. (**D**) GO enrichment analysis of top 10 GO functional terms. (**E**) Structure of potential key active ingredients. (**F**) Component–target–pathway–disease network. **Purple circle**: Anti-oxidant; **green triangles rotated clockwise from the top**: Endocrine resistance, IL-17 signaling pathway, EGFR tyrosine kinase inhibitor resistance, Prostate cancer, HIF-1 signaling pathway, Hepatitis B, Relaxin signaling pathway, Pathways in cancer, VEGF signaling pathway, Proteoglycans in cancer, AGE-RAGE signaling pathway in diabetic complications, Chagas disease, TNF signaling pathway, FoxO signaling pathway, Prolactin signaling pathway, Yersinia infection, Shigellosis, Fluid shear stress and atherosclerosis, Lipid and atherosclerosis, Human cytomegalovirus infection; **red circle**: ORE-N; **inner blue circles rotated clockwise from the top**: 5-Demethoxynobiletin, Sinensetin, 3,5,6,7,8,3′,4′-Heptamethoxyflavone, Chrysin, Luteolin, 5,7,8,4′-Tetramethoxyflavone, Tangeretin, 3,5,6,7-Tetramethoxyflavone, 3′,4′,5′,5,7-Pentamethoxyflavone, 3,5,7,3′4′-Pentamethoxyflavone; **outer blue circles rotated clockwise from the top**: 2-Hydroxycinnamic acid, Echinopsine, L-Pipecolic Acid, Diisobutyl phthalate, Cotinine, 2′-Hydroxygenistein, Pipecolic acid, Urolithin D, 3,4-Dihydroxybenzeneacetic acid, 2′-Hydroxydaidzein, Homogentisic acid, Caffeic acid, Harmol, 6-Geranylnaringenin, L-Tyramine, Methyl 2,4-dihydroxyphenylacetate, 4-Aminosalicylic acid, Cinnamic acid, 2-Hydroxy-3-phenylpropanoic acid, 8-hydroxyquinoline, DL-3-Phenyllactic acid, 5,6,7,4′-Tetramethoxyflavone, 2-Hydroxy-3-(4-Hydroxyphenyl)Propanoic Acid, Mandelic acid, N-Formyl nornicotine, 2-(Formylamino)benzoic acid, 4-Hydroxyphenyllactic Acid, L-Carnitine, 3,5,6,7,8,4′-Hexamethoxyflavone, 2-Hydroxyphenylacetic acid, 5-Hydroxytryptamine, Baptifoline; **yellow rectangles in the first low**: PLAU, CASP1, APP, PTK2, MMP1, ACE, IL2, MAOB; **yellow rectangles in the second low**: CXCL8, MAPK11, PLAT, MAPK1, SERPINE1, ID01, AKT1, MMP2; **yellow rectangles in the third low**: PRKCA, TERT, HMOX, NOX4, BCL2L1, ABL1, EGFR, PARP1; **yellow rec-tangles in the fourth low**: BACE, SNCA, YP3A, MAPK3, NOS3, CDK2, ERBB2, PPARA; **yellow rectangles in the fifth low**: PTGS2, PIK3CA, CYP1A1, NOS2, STAT3, IGF1R, SIRT1, MAPK14; **yellow rectangles in the sixth low**: HMGCR, XDH, ABCG2, FYN, ESR1, MPO, SELE, PLG; **yellow rectangles in the seven low**: NTRK2, TLR4, CASP3, MMP9, GAPDH, ATM, SRC, GSK3B; **yellow rectangles in the eight low**: MAOA, CYP19AT, MAPT, PPARG, CDK1, NQO1, NFE2L2, BCL2; **yellow rectangles in the ninth low**: ICAM1, IKBKB, GSR, ESR2, MMP13, INSR, MMP3.

To systematically explore the molecular mechanisms underlying the antioxidant activity of ORE-N, a network pharmacology approach was employed. Forty-nine compounds from ORE-N (i.e., the top twenty flavonoids, phenolic acids, and alkaloids by relative abundance and with defined CAS numbers) were selected for analysis. Potential targets were predicted using Swiss Target Prediction (probability > 0) and cross-referenced with antioxidant-related genes from GeneCards (keyword: “anti-oxidant”). Venn analysis identified 144 overlapping targets (Figure 8A), which were further analyzed via STRING (confidence score: 0.4) and Cytoscape to construct a protein–protein interaction (PPI) network. Topological analysis revealed 71 core targets (degree ≥ median), with GAPDH, AKT1, STAT3, and CASP3 identified as central hubs (Figure 8B). Subsequently, the components corresponding to these key targets were screened, and the repetitive items were eliminated. Finally, 43 potential active components were identified.

KEGG pathway enrichment analysis identified the AGE-RAGE, HIF-1, IL-17, and FoxO signaling pathways as key mechanisms underlying the antioxidant activity of ORE-N (Figure 8C). For instance, in the AGE-RAGE pathway, ORE-N might suppress ROS generation by inhibiting RAGE/NF-κB activation, similar to the glycation inhibition mediated by polyphenols [99]. In the FoxO pathway, the activation of SOD2 and GPX4 expression suggested an enhancement of endogenous antioxidant defense [100].

GO analysis further linked the identified targets to various biological processes (e.g., positive regulation of transcription), molecular functions (e.g., protein binding), and cellular components (e.g., mitochondrion) (Figure 8D).

The 43 potential active components, 71 core targets, and top 20 pathways were imported into Cytoscape software, and the component–target–pathway–disease visualization network diagram of ORE-N was constructed according to the connectivity between compounds, targets, and signaling pathways. As shown in Figure 8F, these compounds synergistically regulated oxidative stress via multi-target interactions with key pathways, including AGE-RAGE (inhibition of RAGE/NF-κB axis), FoxO (activation of SOD2/GPX4), and IL-17 (suppression of inflammatory-ROS crosstalk). Four polymethoxyflavonoids were identified as core contributors through component–target–pathway–disease network topology (degree ≥ 30), such as tangeretin and 5,7,8,4′-tetramethoxyflavone (Figure 8E). Notably, tangeretin mitigated oxidative damage by modulating the Nrf2 pathway, as evidenced in hepatic models [101]. Polymethoxyflavonoids collectively enhanced cellular antioxidant defenses through coordinated multi-pathway interactions [102]. These findings provided mechanistic support for ORE-N’s observed antioxidant capacity and suggested its therapeutic potential for oxidative stress-related pathologies such as atherosclerosis.

## 4. Conclusions

Bioactive components from *O. raphanipes* were extracted via ultrasonication-assisted ethanolic extraction and fractionated into non-polar/weakly polar (ORE-S) and polar (ORE-N) fractions using silica gel and MAR chromatography. ORE-N showed remarkable enrichment of alkaloids (38×), flavonoids (49×), polyphenols (27×), and VC (30×) versus ORE-C, while ORE-S was predominantly enriched in terpenoids (7×). ORE-N exhibited superior antioxidant activity, strongly correlated with these components. Widely targeted metabolomics revealed ORE-N and ORE-S compositions, while network pharmacology identified 144 antioxidant targets, with 4 polymethoxyflavonoids as core components, acting via AGE-RAGE (NF-κB/ROS suppresion), FoxO (SOD2/GPX4 activation), and IL-17 signaling (modulating inflammatory-ROS crosstalk). GO analysis further linked targets to mitochondrial function and transcriptional regulation. This integrated approach validated ORE-N’s multi-pathway antioxidant mechanism and highlighted enrichment-driven bioactivity shifts, supporting its potential as a natural antioxidant. Future studies should assess the efficacy in cellular/murine models and optimize greener extraction to minimize ethanol residues.

## Figures and Tables

**Table 1:** Characteristics of the MARs.

Resins	D101	AB-8	HPD-450A	NKA-9	ADS-7
Particle size (mm)	0.3–1.25	0.3–1.25	0.3–1.25	0.3–1.25	0.3–1.25
Surface area (m^2^/g)	None	480–520	500–550	500–550	≥100
Average pore diameter (nm)	None	130–140	90–100	100–120	25–30
Polarity	Non-polar	Weak polar	Middle polar	Polar	Strongly polar

## Data Availability

The original contributions presented in this study are included in the article/Supplementary Materials. Further inquiries can be directed to the corresponding author.

## Supplementary Materials

The following supporting information can be downloaded at: https://www.mdpi.com/article/10.3390/foods14162820/s1, Figure S1: The overlay of the total ion current (TIC) chromatograms for the quality control (QC) sample essence spectrometry. (A) Positive ion mode; (B) Negative ion mode.; Figure S2: Pearson’s correlation coefficients among the different sample groups; Table S1: All the identified compounds and their information; Table S2: Differential metabolites and their information.
