# Supplementary material for: Widely Targeted Metabolomic and Network Pharmacology Analyses of Active Compounds Enriched from Ethanolic Extract of Oudemansiella raphanipes
Source: Foods. 2025 Aug 14;14(16):2820. doi: 10.3390/foods14162820 (PMC12385618; doi:10.3390/foods14162820)
Supplement: Supplementary file 1 [file foods-14-02820-s001.zip › foods-3707236-supplementary.pdf]

Supporting Information

Widely targeted metabolomic and network pharmacology of active compounds enriched from ethanolic extract of *Oudemansiella raphanipes*

Zhi Wu [a, 1], Jin Zhao [a], Mengxing Chen [b], Dan Wu [b], Yiyou Wu [b], Shuang Zhu [b], Junbin Lin [a], Renyun Miao [a], Rencai Feng [a], Bingcheng Gan [a, b] *, Tao Wang [a, 1] *

[a] Institute of Urban Agriculture, Chinese Academy of Agricultural Sciences, Chengdu National Agricultural Science & Technology Center, Chengdu 610213, China

[b] College of Food and Biological Engineering, Chengdu University, Chengdu 610106, China

* Corresponding author.

E-mail: wangtao03@caas.cn (T. Wang), ganbingcheng@caas.cn (B. Gan).

[1] These authors contributed equally to this work.

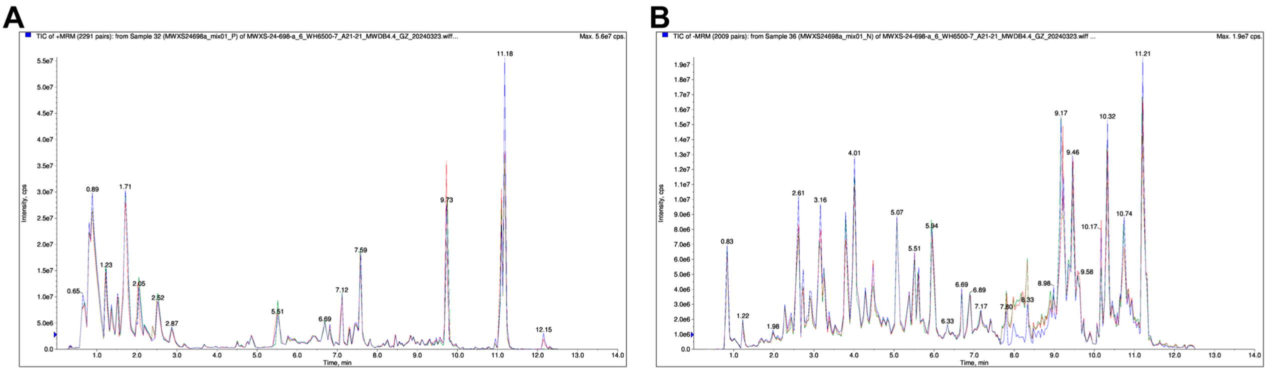

Fig. S1. The overlay of the total ion current (TIC) chromatograms for the quality control (QC) sample essence spectrometry. (A) Positive ion mode; (B) Negative ion mode.

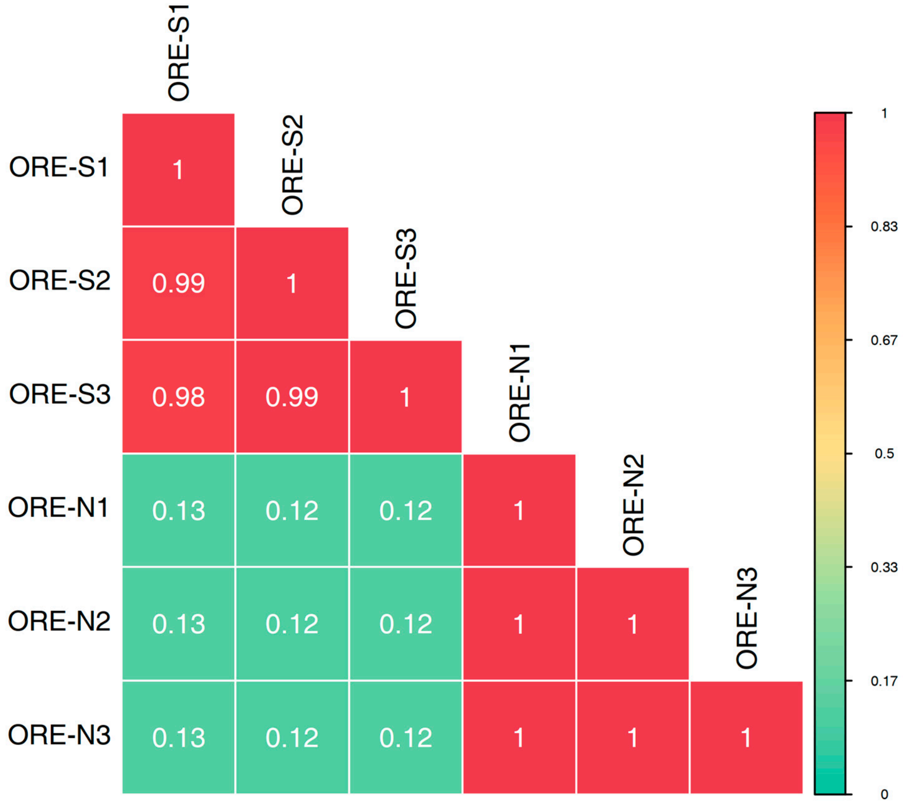

Fig. S2. Pearson's correlation coefficients among the different sample groups.

Table S1. All the identified compounds and their information.

Index	Q1 (Da)	Q3 (Da)	Molecular weight (Da)	Formula	Ionization model	Compounds	Class I	Class II	CAS
Lmqp000377	7.01E+01	6.81E+01	6.91E+01	C4H7N	[M+H]+	Pyrroline	Alkaloids	Pyrrole alkaloids	638-31-3
pme3005	7.41E+01	4.60E+01	7.31E+01	C3H7NO	[M+H]+	N,N-Dimethylformamide	Alkaloids	Alkaloids	68-12-2
pme3200	7.41E+01	5.71E+01	7.31E+01	C2H7N3	[M+H]+	1-Methylguanidine	Alkaloids	Alkaloids	471-29-4
pmb0782	8.61E+01	6.91E+01	8.51E+01	C5H11N	[M+H]+	Piperidine	Alkaloids	Piperidine alkaloids	110-89-4
pme2292	8.91E+01	7.21E+01	8.81E+01	C4H12N2	[M+H]+	Putrescine	Alkaloids	Alkaloids	110-60-1
Zmyn000268	8.90E+01	7.10E+01	9.00E+01	C3H6O3	[M-H]-	2,3-Dihydroxypropanal	Others	Aldehyde compounds	56-82-6
pma6298	9.60E+01	6.81E+01	9.50E+01	C5H5NO	[M+H]+	3-Hydroxypyridine	Alkaloids	Pyridine alkaloids	109-00-2
MWSmce460	1.00E+02	5.61E+01	9.91E+01	C5H9NO	[M+H]+	2-Piperidone	Alkaloids	Piperidine alkaloids	675-20-7

Wcfp001744	1.00E+02	5.80E+01	9.91E+01	C5H9NO	[M+H]+	N-Methylpyrrolidone	Others	Others	872-50-4
MWS1784	1.00E+02	5.51E+01	9.91E+01	C6H13N	[M+H]+	Cyclohexylamine	Alkaloids	Alkaloids	108-91-8
MWStz073	1.02E+02	8.41E+01	1.01E+02	C4H7NO2	[M+H]+	5-Hydroxy-2-pyrrolidinone	Alkaloids	Pyrrole alkaloids	62312-55-4
MWSmce331	1.02E+02	5.61E+01	1.01E+02	C4H7NO2	[M+H]+	Azetidine-2-carboxylic acid*	Alkaloids	Alkaloids	2517-04-6
MWSmce461	1.02E+02	5.61E+01	1.01E+02	C4H7NO2	[M+H]+	L-Azetidine-2-carboxylic acid*	Alkaloids	Alkaloids	2133-34-8
MWS1840	1.02E+02	5.81E+01	1.01E+02	C6H15N	[M+H]+	Triethylamine	Alkaloids	Alkaloids	121-44-8
pmf0256	1.01E+02	8.31E+01	1.02E+02	C6H14O	[M-H]-	3-Methyl-1-pentanol	Others	Alcohol compounds	589-35-5
pme1841	1.03E+02	8.61E+01	1.02E+02	C5H14N2	[M+H]+	Cadaverine	Alkaloids	Alkaloids	462-94-2
pmb0484	1.04E+02	6.01E+01	1.04E+02	C5H14NO+	[M]+	Choline	Alkaloids	Alkaloids	62-49-7
pme2433	1.06E+02	8.81E+01	1.05E+02	C4H11NO2	[M+H]+	Diethanolamine	Alkaloids	Alkaloids	111-42-2

Zmyn002321	1.07E+02	1.06E+02	1.08E+02	C7H8O	[M-H]-	4-Methylphenol	Others	Others	106-44-5
MWS2076	1.10E+02	6.50E+01	1.09E+02	C6H7NO	[M+H]+	2-Aminophenol	Alkaloids	Alkaloids	95-55-6
MWS1854	1.10E+02	6.50E+01	1.09E+02	C6H7NO	[M+H]+	4-Aminophenol	Alkaloids	Alkaloids	123-30-8
Wcdp000839	1.11E+02	5.51E+01	1.10E+02	C6H6O2	[M+H]+	5-methylfurfural	Others	Others	620-02-0
MA10014775	1.09E+02	8.10E+01	1.10E+02	C6H6O2	[M-H]-	Hydroquinone	Others	Others	123-31-9
mws1358	1.09E+02	8.10E+01	1.10E+02	C6H6O2	[M-H]-	Pyrocatechol	Others	Others	120-80-9
mws0601	1.10E+02	6.60E+01	1.11E+02	C5H5NO2	[M-H]-	Pyrrole-2-carboxylic acid	Alkaloids	Pyrrole alkaloids	634-97-9
mws1648	1.11E+02	6.90E+01	1.12E+02	C6H8O2	[M-H]-	Cyclohexane-1,3-dione	Others	Ketone compounds	504-02-9
pme1936	1.14E+02	8.61E+01	1.13E+02	C4H7N3O	[M+H]+	Creatinine	Alkaloids	Alkaloids	60-27-5
HX1345	1.15E+02	6.90E+01	1.14E+02	C5H6O3	[M+H]+	5-(hydroxymethyl)-2(5H)-furanone	Others	Others	-

Yacp000453	1.16E+02	7.01E+01	1.15E+02	C5H9NO2	[M+H]+	3-hydroxy-1-methylpyrrolidin-2-one*	Alkaloids	Pyrrole alkaloids	132996-63-5
Lamp000484	1.16E+02	7.01E+01	1.15E+02	C5H9NO2	[M+H]+	4-Methylazetidine-2-Carboxylic acid*	Alkaloids	Alkaloids	1779725-63-1
MWStz040	1.16E+02	7.01E+01	1.15E+02	C5H9NO2	[M+H]+	Pterolactam*	Alkaloids	Pyrrole alkaloids	38072-88-7
pmb1096	1.18E+02	9.11E+01	1.17E+02	C8H7N	[M+H]+	Indole	Alkaloids	Plumerane	120-72-9
Zasp102439	1.18E+02	9.11E+01	1.17E+02	C8H7N	[M+H]+	m-Aminophenylacetylene	Alkaloids	Alkaloids	54060-30-9
MWSmce548	1.18E+02	5.81E+01	1.17E+02	C5H11NO2	[M+H]+	Betaine	Alkaloids	Alkaloids	107-43-7
Hahp000801	1.18E+02	7.21E+01	1.17E+02	C5H11NO2	[M+H]+	alanine betaine	Alkaloids	Alkaloids	-
Zmmp002106	1.20E+02	7.70E+01	1.19E+02	C3H9N3O2	[M+H]+	4-methyl-1,5,2,3-dioxadiazinan-2-amine	Others	Others	-
pmp001287	1.20E+02	1.03E+02	1.19E+02	C8H9N	[M+H]+	N-Benzylmethylene isomethylamine	Alkaloids	Alkaloids	-
Lmbp000728	1.21E+02	7.70E+01	1.20E+02	C8H8O	[M+H]+	2-Phenyloxirane	Others	Others	20780-54-5

Lmbn002737	1.19E+02	9.30E+01	1.20E+02	C8H8O	[M-H]-	3-Methylbenzaldehyde*	Others	Aldehyde compounds	620-23-5
Lmbn002644	1.19E+02	9.30E+01	1.20E+02	C8H8O	[M-H]-	4-Methylbenzaldehyde*	Others	Aldehyde compounds	104-87-0
Wcdp000970	1.21E+02	1.03E+02	1.20E+02	C8H8O	[M+H]+	acetophenone	Others	Others	98-86-2
pmb0069	1.22E+02	1.05E+02	1.21E+02	C7H7NO	[M+H]+	Benzamide	Alkaloids	Alkaloids	55-21-0
MWSslk106	1.22E+02	1.05E+02	1.21E+02	C8H11N	[M+H]+	2-Phenylethylamine	Alkaloids	Benzylphenylethylamine alkaloids	156-28-5
Lmbn001930	1.21E+02	7.70E+01	1.22E+02	C7H6O2	[M-H]-	2-Hydroxybenzaldehyde (Salicylaldehyde)	Others	Aldehyde compounds	90-02-8
Lmgp002593	1.23E+02	7.70E+01	1.22E+02	C7H6O2	[M+H]+	3-hydroxybenzaldehyde	Others	Aldehyde compounds	100-83-4
mws0628	1.21E+02	9.20E+01	1.22E+02	C7H6O2	[M-H]-	4-Hydroxybenzaldehyde	Others	Aldehyde compounds	123-08-0
pme0241	1.23E+02	7.91E+01	1.22E+02	C7H6O2	[M+H]+	Benzoic acid	Phenolic acids	Phenolic acids	65-85-0
ML10179289	1.05E+02	7.70E+01	1.22E+02	C8H10O	[M-H2O+H]+	2-Phenylethanol	Others	Alcohol compounds	60-12-8

MWS1851	1.21E+02	1.06E+02	1.22E+02	C8H10O	[M-H]-	4-Ethylphenol	Phenolic acids	Phenolic acids	123-07-9
pme1216	1.22E+02	7.80E+01	1.23E+02	C6H5NO2	[M-H]-	2-Picolinic acid	Alkaloids	Pyridine alkaloids	98-98-6
Lmqp002315	1.24E+02	1.07E+02	1.23E+02	C7H9NO	[M+H]+	4-Hydroxybenzylamine	Alkaloids	Phenolamine	696-60-6
HJN025	1.23E+02	1.22E+02	1.24E+02	C7H8O2	[M-H]-	4-Hydroxybenzyl Alcohol	Others	Others	623-05-2
MWSmce274	1.23E+02	9.30E+01	1.24E+02	C7H8O2	[M-H]-	Salicyl Alcohol	Others	Alcohol compounds	90-01-7
NK10264324	1.27E+02	8.10E+01	1.26E+02	C6H6O3	[M+H]+	Phloroglucinol; 1,3,5-Benzenetriol	Others	Others	108-73-6
mws0025	1.25E+02	7.90E+01	1.26E+02	C6H6O3	[M-H]-	Pyrogallol	Others	Others	87-66-1
MWSmce118	1.27E+02	8.10E+01	1.26E+02	C5H6N2O2	[M+H]+	Imidazole-4-Acetic Acid	Alkaloids	Alkaloids	645-65-8
MWSmce127	1.27E+02	8.51E+01	1.26E+02	C3H6N6	[M+H]+	Melamine;1,3,5-Triazine-2,4,6-Triamine	Alkaloids	Alkaloids	108-78-1
MWS1777	1.28E+02	9.31E+01	1.27E+02	C6H6ClN	[M+H]+	3-Chloroaniline	Alkaloids	Alkaloids	108-42-9

MWSmce293	1.28E+02	5.80E+01	1.29E+02	C5H7NOS	[M-H]-	Epigoitrin	Others	Others	1072-93-1
Lmbn000612	1.28E+02	8.20E+01	1.29E+02	C5H7NO3	[M-H]-	1-Pyrroline-4-hydroxy-2-carboxylic acid	Alkaloids	Pyrrole alkaloids	9054-77-7
MWSmce709	1.30E+02	1.03E+02	1.29E+02	C9H7N	[M+H]+	Isoquinoline	Alkaloids	Isoquinoline alkaloids	119-65-3
MWS0811	1.30E+02	8.41E+01	1.29E+02	C6H11NO2	[M+H]+	L-Pipecolic Acid	Alkaloids	Piperidine alkaloids	3105-95-1
Ysjp000315	1.30E+02	5.61E+01	1.29E+02	C6H11NO2	[M+H]+	Pipecolic acid	Alkaloids	Piperidine alkaloids	535-75-1
pme2693	1.31E+02	1.14E+02	1.30E+02	C6H14N2O	[M+H]+	N-Acetylputrescine	Alkaloids	Alkaloids	18233-70-0
pmb0501	1.31E+02	1.14E+02	1.30E+02	C5H14N4	[M+H]+	Agmatine	Alkaloids	Alkaloids	306-60-5
pma3649	1.32E+02	8.61E+01	1.31E+02	C5H9NO3	[M+H]+	5-Aminolevulinic Acid	Alkaloids	Alkaloids	106-60-5
pmp001198	1.32E+02	5.70E+01	1.31E+02	C6H13NO2	[M+H]+	6-Deoxyfagomine	Alkaloids	Piperidine alkaloids	197449-09-5
HX1349	1.33E+02	6.90E+01	1.32E+02	C5H8O4	[M+H]+	dihydro-4-hydroxy-5-hydroxymethyl-2(3H)-furanone	Others	Others	-

MWSslk 225	1.33 E+02	7.70 E+01	1.32E+02	C9H8O	[M+H]+	1-Indanone	Others	Ketone compounds	83-33-0
Samp00 1300	1.34 E+02	1.17 E+02	1.33E+02	C8H7N O	[M+H]+	2-Cyanomethylphenol	Alkaloids	Alkaloids	-
Zmsp00 0306	1.34 E+02	6.81 E+01	1.33E+02	C5H11N O3	[M+H]+	1,4-dideoxy-1,4-imino-D-arabinitol	Alkaloids	Pyrrole alkaloids	-
mws502 8	1.36 E+02	8.01 E+01	1.35E+02	C7H5N O2	[M+H]+	2-Benzoxazolinone	Alkaloids	Alkaloids	59-49-4
Lmxp00 0939	1.36 E+02	1.19 E+02	1.35E+02	C5H5N5	[M+H]+	Zarzissine	Alkaloids	Alkaloids	160568 -14-9
HJKP00 0649	1.36 E+02	9.11 E+01	1.35E+02	C8H9N O	[M+H]+	N-benzylformamide	Alkaloids	Alkaloids	6343- 54-0
MWSm ce466	1.35 E+02	9.20 E+01	1.36E+02	C8H8O2	[M-H]-	4-Hydroxyacetophenone	Others	Ketone compounds	99-93-4
MWS20 99	1.37 E+02	7.70 E+01	1.36E+02	C8H8O2	[M+H]+	4-Methylbenzoic acid	Phenolic acids	Phenolic acids	99-94-5
MWS18 48	1.35 E+02	9.20 E+01	1.36E+02	C8H8O2	[M-H]-	Phenyl acetate	Others	Others	122-79- 2
Zapn005 388	1.35 E+02	9.11 E+01	1.36E+02	C8H8O2	[M-H]-	phenylacetic acid	Phenolic acids	Phenolic acids	103-82- 2

MWS2032	1.37E+02	6.50E+01	1.36E+02	C7H8N2O	[M+H]+	6-Methylnicotinamide	Alkaloids	Pyridine alkaloids	6960-22-1
mws1336	1.38E+02	1.20E+02	1.37E+02	C7H7NO2	[M+H]+	4-Aminobenzoic acid	Phenolic acids	Phenolic acids	150-13-0
mws1078	1.38E+02	9.21E+01	1.37E+02	C7H7NO2	[M+H]+	Anthranilic Acid	Phenolic acids	Phenolic acids	118-92-3
Lmqp002784	1.38E+02	7.70E+01	1.37E+02	C7H7NO2	[M+H]+	Salicylamide	Alkaloids	Alkaloids	65-45-2
pme2268	1.38E+02	9.41E+01	1.37E+02	C7H7NO2	[M+H]+	Trigonelline	Alkaloids	Pyridine alkaloids	535-83-1
pme1002	1.38E+02	1.03E+02	1.37E+02	C8H11NO	[M+H]+	L-Tyramine	Alkaloids	Alkaloids	51-67-2
pma0948	1.38E+02	1.20E+02	1.37E+02	C8H11NO	[M+H]+	Phenylethanolamine	Alkaloids	Alkaloids	7568-93-6
MWSmce581	1.37E+02	9.10E+01	1.38E+02	C7H6O3	[M-H]-	2,4-Dihydroxybenzaldehyde	Others	Aldehyde compounds	95-01-2
Lmbn001981	1.37E+02	9.30E+01	1.38E+02	C7H6O3	[M-H]-	2,5-Dihydroxybenzaldehyde	Others	Aldehyde compounds	1194-98-5
mws0749	1.37E+02	9.30E+01	1.38E+02	C7H6O3	[M-H]-	4-Hydroxybenzoic acid	Phenolic acids	Phenolic acids	99-96-7

MWSH C2067	1.39 E+02	6.50 E+01	1.38E+02	C7H6O3	[M+H]+	Protocatechualdehyde	Others	Aldehyde compounds	139-85-5
mws0336	1.37 E+02	9.31 E+01	1.38E+02	C6H6N2 O2	[M-H]-	Urocanic acid	Alkaloids	Alkaloids	104-98-3
mws2368	1.37 E+02	1.19 E+02	1.38E+02	C8H10O 2	[M-H]-	Tyrosol; 4-Hydroxyphenylethanol	Others	Alcohol compounds	501-94-0
MWS2044	1.39 E+02	6.90 E+01	1.38E+02	C9H14O	[M+H]+	Isophorone	Others	Ketone compounds	78-59-1
Zmpn001713	1.38 E+02	1.08 E+02	1.39E+02	C6H5N O3	[M-H]-	3-Nitrophenol	Others	Others	554-84-7
pme2828	1.40 E+02	9.90 E+01	1.39E+02	C6H5N O3	[M+H]+	4-Nitrophenol	Others	Others	100-02-7
pmf0132	1.41 E+02	1.23 E+02	1.40E+02	C6H4O4	[M+H]+	Coumalic acid	Phenolic acids	Phenolic acids	500-05-0
mws0704	1.40 E+02	7.90 E+01	1.41E+02	C2H8N O4P	[M-H]-	O-Phosphorylethanolamine	Alkaloids	Alkaloids	1071-23-4
pma2987	1.42 E+02	1.24 E+02	1.41E+02	C6H11N 3O	[M+H]+	Histidinol	Alkaloids	Alkaloids	501-28-0
pmb0764	1.44 E+02	1.13 E+02	1.43E+02	C6H9N OS	[M+H]+	4-Methyl-5-thiazoleethanol	Others	Others	137-00-8

Lmcp000611	1.44E+02	8.41E+01	1.43E+02	C5H9N3O2	[M+H]+	2-Amino-4,5-dihydro-1H-imidazole-4-acetic acid	Alkaloids	Alkaloids	69098-41-5
Zajp000573	1.44E+02	8.41E+01	1.43E+02	C7H13NO2	[M+H]+	N-(4-oxopentyl)-acetamide	Alkaloids	Alkaloids	-
MWSmce157	1.44E+02	5.81E+01	1.43E+02	C7H13NO2	[M+H]+	Stachydrine	Alkaloids	Pyrrole alkaloids	471-87-4
Hmcp000405	1.44E+02	8.41E+01	1.43E+02	C8H17NO	[M+H]+	Dihydroisopelletierine	Alkaloids	Piperidine alkaloids	-
MWSslk071	1.44E+02	5.71E+01	1.43E+02	C8H17NO	[M+H]+	Valpromide	Alkaloids	Alkaloids	2430-27-5
MWS2066	1.43E+02	1.15E+02	1.44E+02	C10H8O	[M-H]-	1-Naphthol*	Others	Others	90-15-3
MWS1875	1.43E+02	1.15E+02	1.44E+02	C10H8O	[M-H]-	2-Naphthol*	Others	Others	135-19-3
MWS3020	1.45E+02	8.61E+01	1.44E+02	C7H16N2O	[M+H]+	N-Acetylcadaverine	Alkaloids	Alkaloids	32343-73-0
MA10074217	1.44E+02	1.15E+02	1.45E+02	C9H7NO	[M-H]-	4-Hydroxyquinoline	Alkaloids	Quinoline alkaloids	611-36-9
Wcjp002598	1.46E+02	9.11E+01	1.45E+02	C9H7NO	[M+H]+	8-hydroxyquinoline	Alkaloids	Quinoline alkaloids	148-24-3

mws0103	1.46E+02	9.11E+01	1.45E+02	C9H7NO	[M+H]+	Indole-3-carboxaldehyde	Alkaloids	Plumerane	487-89-8
Wbmp002283	1.46E+02	1.18E+02	1.45E+02	C9H7NO	[M+H]+	A-hydroxyquinoline	Alkaloids	Alkaloids	-
Wbjp000520	1.46E+02	8.21E+01	1.45E+02	C6H11NO3	[M+H]+	4-Hydroxypipecolic acid	Alkaloids	Alkaloids	14228-16-1
mws0017	1.46E+02	7.21E+01	1.45E+02	C7H19N3	[M+H]+	Spermidine	Alkaloids	Alkaloids	124-20-9
MWSmce128	1.47E+02	1.30E+02	1.46E+02	C8H6N2O	[M+H]+	4(3H)-Quinazolinone	Alkaloids	Alkaloids	491-36-1
MWS20194	1.49E+02	1.03E+02	1.48E+02	C9H8O2	[M+H]+	Cinnamic acid	Phenolic acids	Phenolic acids	140-10-3
Latp011219	1.49E+02	1.21E+02	1.48E+02	C10H12O	[M+H]+	3,5-Dimethyl-2,3-dihydrobenzofuran	Others	Others	-
MWSmce698	1.49E+02	6.50E+01	1.48E+02	C10H12O	[M+H]+	2',4'-Dimethylacetophenone	Others	Ketone compounds	89-74-7
Hmmn001882	1.48E+02	9.21E+01	1.49E+02	C8H7NO2	[M-H]-	2,5-Dihydroxy-Indole	Alkaloids	Plumerane	-
MWSmce653	1.49E+02	1.06E+02	1.50E+02	C9H10O2	[M-H]-	2'-Hydroxy-4'-Methylacetophenone	Phenolic acids	Phenolic acids	6921-64-8

MWSmce573	1.49E+02	1.05E+02	1.50E+02	C9H10O2	[M-H]-	3,4-Dimethylbenzoic acid	Phenolic acids	Phenolic acids	619-04-5
MWSmce283	1.49E+02	9.20E+01	1.50E+02	C9H10O2	[M-H]-	4'-Hydroxypropiophenone	Others	Ketone compounds	70-70-2
Zamp007073	1.51E+02	1.05E+02	1.50E+02	C9H10O2	[M+H]+	4'-Methoxyacetophenone	Others	Ketone compounds	100-06-1
mws0008	1.49E+02	1.05E+02	1.50E+02	C9H10O2	[M-H]-	Hydrocinnamic acid	Phenolic acids	Phenolic acids	501-52-0
Lmxn006423	1.50E+02	1.22E+02	1.51E+02	C7H5NOS	[M-H]-	2(3H)-Benzothiazolone	Alkaloids	Alkaloids	934-34-9
Lmtp003569	1.52E+02	1.06E+02	1.51E+02	C8H9NO2	[M+H]+	Mandelamide	Alkaloids	Alkaloids	24008-62-6
MWStz054	1.52E+02	9.11E+01	1.51E+02	C9H13NO	[M+H]+	2,4,6,6-Tetramethyl-3(6H)-pyridinone	Alkaloids	Pyridine alkaloids	203524-64-5
MWS5185	1.51E+02	1.09E+02	1.52E+02	C8H8O3	[M-H]-	2',4'-Dihydroxyacetophenone	Others	Ketone compounds	89-84-9
MWSmce363	1.51E+02	1.08E+02	1.52E+02	C8H8O3	[M-H]-	2,5-Dihydroxyacetophenone	Others	Ketone compounds	490-78-8
Zmyn002323	1.51E+02	1.07E+02	1.52E+02	C8H8O3	[M-H]-	2-Hydroxyphenylacetic acid	Phenolic acids	Phenolic acids	614-75-5

MWSmce468	1.51E+02	7.70E+01	1.52E+02	C8H8O3	[M-H]-	2-hydroxymethyl benzoic acid	Phenolic acids	Phenolic acids	612-20-4
MWSmce454	1.51E+02	6.50E+01	1.52E+02	C8H8O3	[M-H]-	3,5-Dihydroxyacetophenone	Others	Ketone compounds	51863-60-6
MWS2058	1.51E+02	1.07E+02	1.52E+02	C8H8O3	[M-H]-	3-Methylsalicylic Acid	Phenolic acids	Phenolic acids	83-40-9
Wchn003237	1.51E+02	7.70E+01	1.52E+02	C8H8O3	[M-H]-	3-hydroxy-5-methoxybenzaldehyde	Phenolic acids	Phenolic acids	57179-35-8
MWS4301	1.51E+02	1.07E+02	1.52E+02	C8H8O3	[M-H]-	3-hydroxyphenylacetic acid*	Phenolic acids	Phenolic acids	621-37-4
MWSmce370	1.51E+02	1.07E+02	1.52E+02	C8H8O3	[M-H]-	4-Hydroxyphenylacetic acid*	Phenolic acids	Phenolic acids	156-38-7
pme2362	1.51E+02	1.07E+02	1.52E+02	C8H8O3	[M-H]-	Mandelic acid	Phenolic acids	Phenolic acids	90-64-2
Zmgn004894	1.51E+02	1.36E+02	1.52E+02	C8H8O3	[M-H]-	Methyl 4-hydroxybenzoate	Phenolic acids	Phenolic acids	99-76-3
mws0145	1.51E+02	9.30E+01	1.52E+02	C8H8O3	[M-H]-	O-Anisic acid (2-Methoxybenzoic acid)*	Phenolic acids	Phenolic acids	529-75-9
MWS1846	1.51E+02	9.30E+01	1.52E+02	C8H8O3	[M-H]-	Phenoxyacetic acid*	Phenolic acids	Phenolic acids	122-59-8

mws0458	1.51 E+02	1.36 E+02	1.52E+02	C8H8O3	[M-H]-	Vanillin; 4-Hydroxy-3-Methoxybenzaldehyde	Others	Aldehyde compounds	121-33-5
mws0943	1.51 E+02	7.10 E+01	1.52E+02	C7H8N2O2	[M-H]-	1,4-Dihydro-1-Methyl-4-oxo-3-pyridinecarboxamide	Alkaloids	Pyridine alkaloids	769-49-3
MWS0687	1.53 E+02	1.08 E+02	1.52E+02	C7H8N2O2	[M+H]+	1-Methyl-6-Oxo-1,6-Dihydropyridine-3-Carboxamide	Alkaloids	Pyridine alkaloids	701-44-0
MWSmce713	1.51 E+02	1.06 E+02	1.52E+02	C9H12O2	[M-H]-	3-(4-Hydroxyphenyl)-1-propanol	Others	Alcohol compounds	10210-17-0
MWS1962	1.53 E+02	8.31 E+01	1.52E+02	C10H16O	[M+H]+	cis-Citral	Terpenoids	Monoterpenoids	5392-40-5
mws0444	1.52 E+02	1.08 E+02	1.53E+02	C7H7NO3	[M-H]-	3-Aminosalicylic acid	Phenolic acids	Phenolic acids	570-23-0
mws0596	1.54 E+02	1.36 E+02	1.53E+02	C7H7NO3	[M+H]+	3-Hydroxyanthranilic acid	Alkaloids	Alkaloids	548-93-6
ML10177402	1.52 E+02	1.08 E+02	1.53E+02	C7H7NO3	[M-H]-	4-Aminosalicylic acid	Phenolic acids	Phenolic acids	65-49-6
Hmlp000935	1.54 E+02	1.37 E+02	1.53E+02	C8H11NO2	[M+H]+	Vanillylamine	Alkaloids	Alkaloids	1196-92-5
mws0639	1.53 E+02	1.09 E+02	1.54E+02	C7H6O4	[M-H]-	2,3-Dihydroxybenzoic Acid*	Phenolic acids	Phenolic acids	303-38-8

mws088 5	1.53 E+02	1.09 E+02	1.54E+02	C7H6O4	[M-H]-	2,4-Dihydroxybenzoic acid	Phenolic acids	Phenolic acids	89-86-1
mws018 0	1.53 E+02	1.09 E+02	1.54E+02	C7H6O4	[M-H]-	2,5-Dihydroxybenzoic acid; Gentisic Acid*	Phenolic acids	Phenolic acids	490-79- 9
mws018 3	1.53 E+02	1.09 E+02	1.54E+02	C7H6O4	[M-H]-	3,4-Dihydroxybenzoic acid (Protocatechuic acid)*	Phenolic acids	Phenolic acids	99-50-3
MWSH C20168	1.53 E+02	1.09 E+02	1.54E+02	C7H6O4	[M-H]-	Methyl cumalate	Others	Lactones	6018- 41-3
MWSm ce264	1.53 E+02	1.23 E+02	1.54E+02	C8H10O 3	[M-H]-	Hydroxytyrosol	Others	Alcohol compounds	10597- 60-1
MWSm ce663	1.53 E+02	1.20 E+02	1.54E+02	C8H10O 3	[M-H]-	Vanillyl alcohol	Phenolic acids	Phenolic acids	498-00- 0
Lhlp120 612	1.55 E+02	8.11 E+01	1.54E+02	C10H18 O	[M+H]+	Menthone	Terpenoids	Monoterpenoids	14073- 97-3
MA100 40889	1.54 E+02	1.23 E+02	1.55E+02	C6H5N O4	[M-H]-	4-Nitrocatechol	Others	Others	3316- 09-4
Zbzp000 519	1.56 E+02	1.10 E+02	1.55E+02	C8H13N O2	[M+H]+	Retronecine	Alkaloids	Pyrrole alkaloids	480-85- 3
MWSslk 051	1.56 E+02	8.41 E+01	1.55E+02	C8H13N O2	[M+H]+	Scopine	Alkaloids	Tropan alkaloids	498-45- 3

pmb0819	1.57E+02	1.30E+02	1.56E+02	C10H8N2	[M+H]+	3-Indoleacetonitrile	Alkaloids	Plumerane	771-51-7
MWSmce158	1.57E+02	5.51E+01	1.56E+02	C10H20O	[M+H]+	Citronellol	Others	Alcohol compounds	1117-61-9
Zmbp000490	1.58E+02	1.12E+02	1.57E+02	C8H15NO2	[M+H]+	Valerine	Alkaloids	Alkaloids	-
Smbp002161	1.58E+02	8.41E+01	1.57E+02	C9H19NO	[M+H]+	8-ethylnorlobelol	Alkaloids	Piperidine alkaloids	-
MWS0205	1.57E+02	9.70E+01	1.58E+02	C4H6N4O3	[M-H]-	Allantoin	Alkaloids	Alkaloids	97-59-6
Lskp211263	1.59E+02	7.91E+01	1.58E+02	C8H14O3	[M+H]+	Rengyoxide	Others	Others	-
Zmsp000758	1.58E+02	7.01E+01	1.58E+02	C8H15NO2	[M+H]+	1-(Hydroxymethyl)hexahydro-1h-pyrrolizin-2-ol	Alkaloids	Pyrrole alkaloids	-
pmf0174	1.57E+02	1.57E+02	1.58E+02	C10H22O	[M-H]-	1-Decanol*	Others	Alcohol compounds	112-30-1
pmf0175	1.57E+02	1.57E+02	1.58E+02	C10H22O	[M-H]-	2-Decanol*	Others	Alcohol compounds	1120-06-5
Lwhp010633	1.60E+02	1.15E+02	1.59E+02	C10H9NO	[M+H]+	Echinopsine	Alkaloids	Quinoline alkaloids	83-54-5

pmp001196	1.60E+02	9.81E+01	1.59E+02	C7H13NO3	[M+H]+	Calystegine A3	Alkaloids	Tropan alkaloids	131580-36-4
Zahn004036	1.59E+02	1.15E+02	1.60E+02	C10H8O2	[M-H]-	2,3-Dihydro-1,4-naphthoquinone	Quinones	Quinones	21545-31-3
mws0005	1.61E+02	1.44E+02	1.60E+02	C10H12N2	[M+H]+	Tryptamine	Alkaloids	Plumerane	61-54-1
Lmgp001898	1.62E+02	1.16E+02	1.61E+02	C9H7NO2	[M+H]+	4,6-Dihydroxyquinoline	Alkaloids	Quinoline alkaloids	3517-61-1
mws1417	1.60E+02	1.16E+02	1.61E+02	C9H7NO2	[M-H]-	Indole-3-carboxylic acid*	Alkaloids	Plumerane	771-50-6
mws0102	1.60E+02	1.16E+02	1.61E+02	C9H7NO2	[M-H]-	Indole-5-carboxylic acid*	Alkaloids	Plumerane	1670-81-1
mws1346	1.62E+02	9.81E+01	1.61E+02	C6H11NO4	[M+H]+	DL-2-Aminoadipic acid	Alkaloids	Alkaloids	542-32-5
mws1320	1.60E+02	1.30E+02	1.61E+02	C10H11NO	[M-H]-	Tryptophol	Alkaloids	Plumerane	526-55-6
Lmcp000282	1.62E+02	8.50E+01	1.61E+02	C7H15NO3	[M+H]+	L-Carnitine	Alkaloids	Alkaloids	541-15-1
pme2996	1.61E+02	1.17E+02	1.62E+02	C9H6O3	[M-H]-	4-Hydroxycoumarin	Lignans and Coumarins	Coumarins	1076-38-6

Wcfp005519	1.63E+02	1.33E+02	1.62E+02	C9H6O3	[M+H]+	5-hydroxyindene-1,3-dione	Terpenoids	Sesquiterpenoids	131977-61-2
MWSHY0141	1.63E+02	1.07E+02	1.62E+02	C9H6O3	[M+H]+	7-Hydroxy-4-chromone	Others	Chromone	59887-89-7
Zmpn003044	1.61E+02	1.33E+02	1.62E+02	C9H6O3	[M-H]-	7-Hydroxycoumarin;Umbelliferone	Lignans and Coumarins	Coumarins	93-35-6
MWSslk072	1.63E+02	1.46E+02	1.62E+02	C8H6N2O2	[M+H]+	Benzoyleneurea	Alkaloids	Alkaloids	86-96-4
pmf0440	1.63E+02	9.11E+01	1.62E+02	C10H10O2	[M+H]+	4-MethoxycinnaMaldehyde	Others	Aldehyde compounds	1963-36-6
MWSmce275	1.63E+02	1.07E+02	1.62E+02	C10H10O2	[M+H]+	5-Hydroxy-1-tetralone	Others	Ketone compounds	28315-93-7
mws1478	1.63E+02	1.30E+02	1.62E+02	C10H14N2	[M+H]+	Nicotine	Alkaloids	Pyridine alkaloids	54-11-5
Lmmn001643	1.63E+02	1.19E+02	1.64E+02	C9H8O3	[M-H]-	2-Hydroxycinnamic acid*	Phenolic acids	Phenolic acids	583-17-5
MWSmce248	1.63E+02	1.19E+02	1.64E+02	C9H8O3	[M-H]-	3-Hydroxycinnamic Acid*	Phenolic acids	Phenolic acids	14755-02-3
MWSCX017	1.65E+02	1.19E+02	1.64E+02	C9H8O3	[M+H]+	4-hydroxyphenyl acrylaldehyde	Others	Aldehyde compounds	-

pme1439	1.65E+02	1.19E+02	1.64E+02	C9H8O3	[M+H]+	p-Coumaric acid	Phenolic acids	Phenolic acids	501-98-4
Lmbn002648	1.63E+02	1.19E+02	1.64E+02	C9H8O3	[M-H]-	α-Hydroxycinnamic Acid*	Phenolic acids	Phenolic acids	5801-57-0
MWSmce712	1.65E+02	9.11E+01	1.64E+02	C10H12O2	[M+H]+	Ethyl phenylacetate	Phenolic acids	Phenolic acids	101-97-3
pme3083	1.64E+02	1.20E+02	1.65E+02	C8H7NO3	[M-H]-	2-(Formylamino)benzoic acid	Phenolic acids	Phenolic acids	3342-77-6
pmb0037	1.66E+02	1.21E+02	1.65E+02	C10H15NO	[M+H]+	Hordenine	Alkaloids	Alkaloids	539-15-1
Zmhn001420	1.65E+02	1.21E+02	1.66E+02	C8H6O4	[M-H]-	5,7-Dihydroxy-1(3H)-isobenzofuranone	Others	Lactones	27979-58-4
pme0282	1.65E+02	7.70E+01	1.66E+02	C8H6O4	[M-H]-	Phthalic acid	Phenolic acids	Phenolic acids	88-99-3
MWSmce583	1.65E+02	9.10E+01	1.66E+02	C8H6O4	[M-H]-	Piperonylic acid	Phenolic acids	Phenolic acids	94-53-1
pme0281	1.65E+02	1.21E+02	1.66E+02	C8H6O4	[M-H]-	Terephthalic acid	Phenolic acids	Phenolic acids	100-21-0
Lmbn005172	1.65E+02	1.19E+02	1.66E+02	C9H10O3	[M-H]-	2,6-Dimethoxybenzaldehyde	Others	Aldehyde compounds	3392-97-0

Lmrn003000	1.65E+02	1.03E+02	1.66E+02	C9H10O3	[M-H]-	2-Hydroxy-3-phenylpropanoic acid	Phenolic acids	Phenolic acids	7326-19-4
mws0346	1.65E+02	1.06E+02	1.66E+02	C9H10O3	[M-H]-	3-(3-Hydroxyphenyl)-propionic acid	Phenolic acids	Phenolic acids	621-54-5
pme3381	1.65E+02	1.06E+02	1.66E+02	C9H10O3	[M-H]-	3-Hydroxyphenylacetic Acid Methyl Ester	Phenolic acids	Phenolic acids	42058-59-3
MWS0274	1.65E+02	1.19E+02	1.66E+02	C9H10O3	[M-H]-	DL-3-Phenyllactic acid	Phenolic acids	Phenolic acids	828-01-3
MWS1839	1.65E+02	9.20E+01	1.66E+02	C9H10O3	[M-H]-	Ethylparaben	Phenolic acids	Phenolic acids	120-47-8
Hahn002372	1.65E+02	1.50E+02	1.66E+02	C9H10O3	[M-H]-	apocynin	Others	Others	-
pmn001681	1.65E+02	1.49E+02	1.66E+02	C10H14O2	[M-H]-	1-(4-Methoxyphenyl)-1-propanol	Others	Alcohol compounds	5349-60-0
NK10253223	1.68E+02	9.41E+01	1.67E+02	C8H9NO3	[M+H]+	2-Amino-3-methoxybenzoic acid	Phenolic acids	Phenolic acids	3177-80-8
Hmtp000776	1.68E+02	1.05E+02	1.67E+02	C8H9NO3	[M+H]+	4,5,6-Trihydroxy-2-cyclohexen-1-ylideneacetonitrile	Alkaloids	Alkaloids	-
MWStz147	1.68E+02	1.35E+02	1.67E+02	C9H13NO2	[M+H]+	Synephrine; 4-[1-Hydroxy-2-(methylamino)ethyl]phenol	Alkaloids	Phenolamine	94-07-5

MWSmce490	1.67E+02	1.23E+02	1.68E+02	C8H8O4	[M-H]-	2',4',6'-Trihydroxyacetophenone	Others	Ketone compounds	480-66-0
pme2598	1.67E+02	1.23E+02	1.68E+02	C8H8O4	[M-H]-	3,4-Dihydroxybenzeneacetic acid*	Phenolic acids	Phenolic acids	102-32-9
MWS2417	1.67E+02	1.21E+02	1.68E+02	C8H8O4	[M-H]-	3-Hydroxymandelate	Phenolic acids	Phenolic acids	17119-15-2
MWS3136	1.67E+02	1.08E+02	1.68E+02	C8H8O4	[M-H]-	4-Methoxysalicylic Acid	Phenolic acids	Phenolic acids	2237-36-7
Hmtp000827	1.69E+02	6.71E+01	1.68E+02	C8H8O4	[M+H]+	Griffonilide	Others	Lactones	61371-55-9
pme1292	1.67E+02	1.23E+02	1.68E+02	C8H8O4	[M-H]-	Homogentisic acid*	Phenolic acids	Phenolic acids	451-13-8
MWSmce501	1.67E+02	1.08E+02	1.68E+02	C8H8O4	[M-H]-	Protocatechuic Acid Methyl Ester	Phenolic acids	Phenolic acids	2150-43-8
mws0028	1.67E+02	1.08E+02	1.68E+02	C8H8O4	[M-H]-	Vanillic acid	Phenolic acids	Phenolic acids	121-34-6
MWSmce208	1.69E+02	1.15E+02	1.68E+02	C11H8N2	[M+H]+	Norharmane; Beta-Carboline	Alkaloids	Plumerane	244-63-3
mws4193	1.67E+02	1.21E+02	1.68E+02	C9H12O3	[M-H]-	Homovanillic alcohol; 4-Hydroxy-3-methoxyphenethanol	Others	Alcohol compounds	2380-78-1

pme3081	1.69E+02	1.34E+02	1.68E+02	C8H12N2O2	[M+H]+	pyridoxamine	Alkaloids	Pyridine alkaloids	85-87-0
NK10252689	1.68E+02	1.23E+02	1.69E+02	C7H7NO4	[M-H]-	2-Methoxy-5-nitrophenol	Others	Others	636-93-1
Zmpp000906	1.70E+02	1.07E+02	1.69E+02	C8H11NO3	[M+H]+	Norepinephrine	Alkaloids	Alkaloids	51-41-2
MWSslk100	1.70E+02	9.31E+01	1.69E+02	C12H11N	[M+H]+	Diphenylamine	Alkaloids	Alkaloids	122-39-4
MWStz063	1.70E+02	1.52E+02	1.69E+02	C10H19NO	[M+H]+	2-Ethyl-2,6,6-trimethylpiperidin-4-one	Alkaloids	Piperidine alkaloids	133568-79-3
Zmzn000078	1.69E+02	7.90E+01	1.70E+02	C3H7O6P	[M-H]-	Dihydroxyacetone phosphate	Others	Ketone compounds	57-04-5
MWSslk138	1.69E+02	1.51E+02	1.70E+02	C7H6O5	[M-H]-	2,3,4-Trihydroxybenzoic acid	Phenolic acids	Phenolic acids	610-02-6
mws0024	1.69E+02	1.25E+02	1.70E+02	C7H6O5	[M-H]-	Gallic acid	Phenolic acids	Phenolic acids	149-91-7
MWS1812	1.71E+02	5.51E+01	1.70E+02	C11H22O	[M+H]+	2-Undecanone	Others	Ketone compounds	112-12-9
Hmcp000409	1.72E+02	9.81E+01	1.71E+02	C10H21NO	[M+H]+	Halosaline	Alkaloids	Piperidine alkaloids	26648-71-5

MWSslk 070	1.73 E+02	7.01 E+01	1.72E+02	C8H20N 4	[M+H]+	Cyclen	Alkaloids	Alkaloids	294-90-6
MWS51 57	1.72 E+02	8.00 E+01	1.73E+02	C6H7N O3S	[M-H]-	2-Aminobenzenesulfonic Acid	Alkaloids	Alkaloids	88-21-1
MWS51 69	1.72 E+02	1.28 E+02	1.73E+02	C10H7N O2	[M-H]-	Quinoline-4-carboxylic acid	Alkaloids	Quinoline alkaloids	486-74-8
Lwhp01 0632	1.74 E+02	1.59 E+02	1.73E+02	C11H11 NO	[M+H]+	1,2-dimethylquinolin-4(1H)-one	Alkaloids	Quinoline alkaloids	6760-40-3
MWSprf 047	1.75 E+02	1.47 E+02	1.74E+02	C10H6O 3	[M+H]+	Juglone; 5-Hydroxy-1,4-naphthoquinone	Quinones	Quinones	481-39-0
MWSprf 138	1.76 E+02	1.03 E+02	1.75E+02	C10H9N O2	[M+H]+	Indole-3-acetic acid (IAA)	Alkaloids	Plumerane	87-51-4
Zahp006 076	1.77 E+02	1.31 E+02	1.76E+02	C10H8O 3	[M+H]+	2-Hydroxy-2,3-dihydronaphthalene-1,4-dione	Quinones	Quinones	63534-43-0
Wagp00 2530	1.77 E+02	1.17 E+02	1.76E+02	C10H8O 3	[M+H]+	2-Methoxy-1-benzofuran-5-carbaldehyde	Others	Aldehyde compounds	-
Zahp007 439	1.77 E+02	1.49 E+02	1.76E+02	C10H8O 3	[M+H]+	3,4-Methylenedioxycinnamaldehyde	Others	Aldehyde compounds	58095-77-5
mws013 9	1.75 E+02	1.47 E+02	1.76E+02	C10H8O 3	[M-H]-	7-Hydroxy-4-methylcoumarin	Lignans and Coumarins	Coumarins	90-33-5

pmf0323	1.77E+02	1.18E+02	1.76E+02	C10H12N2O	[M+H]+	Cotinine; (S)-1-Methyl-5-(3-pyridinyl)-2-pyrrolidinone	Alkaloids	Pyridine alkaloids	486-56-6
Wayp002004	1.77E+02	1.32E+02	1.76E+02	C10H12N2O	[M+H]+	N-Formyl nornicotine	Alkaloids	Pyridine alkaloids	3000-81-5
pmb0774	1.77E+02	1.60E+02	1.76E+02	C10H12N2O	[M+H]+	N-Hydroxytryptamine*	Alkaloids	Plumerane	-
pme2024	1.77E+02	1.60E+02	1.76E+02	C10H12N2O	[M+H]+	Serotonin; 5-Hydroxytryptamine*	Alkaloids	Plumerane	50-67-9
mws1013	1.77E+02	1.33E+02	1.78E+02	C9H6O4	[M-H]-	Esculetin (6,7-Dihydroxycoumarin)	Lignans and Coumarins	Coumarins	305-01-1
Jmzn006005	1.77E+02	1.17E+02	1.78E+02	C10H10O3	[M-H]-	3,4-methylenedioxy cinnamyl alcohol	Others	Alcohol compounds	58095-76-4
pmb2795	1.77E+02	1.33E+02	1.78E+02	C10H10O3	[M-H]-	4-Methoxycinnamic acid	Phenolic acids	Phenolic acids	830-09-1
Haxp007330	1.79E+02	1.35E+02	1.78E+02	C10H10O3	[M+H]+	p-Methoxycinnamic acid	Phenolic acids	Phenolic acids	-
pme3255	1.79E+02	1.23E+02	1.78E+02	C11H14O2	[M+H]+	Methyleugenol	Others	Others	93-15-2
Lmyp001138	1.79E+02	1.30E+02	1.78E+02	C10H14N2O	[M+H]+	6-Hydroxynicotine	Alkaloids	Pyridine alkaloids	10516-09-3

Wagp001323	1.80E+02	1.21E+02	1.79E+02	C11H17NO	[M+H]+	4-[2-(1-methylethyl)aminoethyl]phenol	Alkaloids	Quinoline alkaloids	-
pmp000725	1.80E+02	1.62E+02	1.79E+02	C11H17NO	[M+H]+	Methylephedrine	Alkaloids	Alkaloids	17605-71-9
MWSmce001	1.81E+02	1.07E+02	1.80E+02	C9H8O4	[M+H]+	5-Acetylsalicylic acid	Phenolic acids	Phenolic acids	13110-96-8
mws2212	1.79E+02	1.35E+02	1.80E+02	C9H8O4	[M-H]-	Caffeic acid	Phenolic acids	Phenolic acids	331-39-5
Zblp002750	1.81E+02	1.21E+02	1.80E+02	C10H12O3	[M+H]+	Methyl-3-(3-hydroxyphenyl)Propionate	Phenolic acids	Phenolic acids	61389-68-2
MWS2070	1.79E+02	9.20E+01	1.80E+02	C10H12O3	[M-H]-	Propyl 4-hydroxybenzoate	Phenolic acids	Phenolic acids	94-13-3
Zbzp007397	1.81E+02	1.35E+02	1.80E+02	C11H16O2	[M+H]+	5,6,7,7a-tetrahydro-4,4,7a-trimethyl-2(4H)-benzofuranone	Others	Others	15356-74-8
Cmyp007180	1.81E+02	1.07E+02	1.80E+02	C11H16O2	[M+H]+	Dihydroactinidiolide	Terpenoids	Monoterpenoids	17092-92-1
MWStz070	1.82E+02	9.11E+01	1.81E+02	C9H11NO3	[M+H]+	N-(2-Hydroxy-4-methoxyphenyl)acetamide	Alkaloids	Alkaloids	58469-06-0
Lmrn001951	1.81E+02	1.35E+02	1.82E+02	C9H10O4	[M-H]-	2-Hydroxy-3-(4-Hydroxyphenyl)Propanoic Acid*	Phenolic acids	Phenolic acids	23508-35-2

mws0160	1.81E+02	1.09E+02	1.82E+02	C9H10O4	[M-H]-	3,4-Dihydroxybenzoic Acid Ethyl Ester (Protocatechuic acid ethyl ester)	Phenolic acids	Phenolic acids	3943-89-3
MWS5206	1.81E+02	1.35E+02	1.82E+02	C9H10O4	[M-H]-	4-Hydroxyphenyllactic Acid*	Phenolic acids	Phenolic acids	306-23-0
mws0117	1.81E+02	1.22E+02	1.82E+02	C9H10O4	[M-H]-	Homovanillic acid; 4-Hydroxy-3-methoxyphenylacetic acid	Phenolic acids	Phenolic acids	306-08-1
Hmtn001288	1.81E+02	1.35E+02	1.82E+02	C9H10O4	[M-H]-	Methyl 2,4-dihydroxyphenylacetate*	Phenolic acids	Phenolic acids	67828-42-6
Lahp002797	1.83E+02	1.23E+02	1.82E+02	C9H10O4	[M+H]+	Methyl Orsellinate	Phenolic acids	Phenolic acids	3187-58-4
ZbWn004505	1.81E+02	9.70E+01	1.82E+02	C6H14O4S	[M-H]-	Hexyl Hydrogen Sulfate	Others	Others	3233-49-6
Wagp006039	1.83E+02	9.90E+01	1.82E+02	C6H15O4P	[M+H]+	2-Hexylphosphoric Acid	Others	Others	-
Lmtn004929	1.81E+02	1.35E+02	1.82E+02	C10H14O3	[M-H]-	Dihydroconiferyl Alcohol	Phenolic acids	Phenolic acids	2305-13-7
Zmpp001726	1.84E+02	1.24E+02	1.83E+02	C9H13NO3	[M+H]+	Epinephrine	Alkaloids	Alkaloids	51-43-4
MA10002917	1.83E+02	1.09E+02	1.84E+02	C6H4N2O5	[M-H]-	2,4-Dinitrophenol	Others	Others	51-28-5

Lmqn001857	1.83E+02	1.39E+02	1.84E+02	C8H8O5	[M-H]-	3,5-Dihydroxy-4-methoxybenzoic acid; 4-O-Methylgallic Acid	Phenolic acids	Phenolic acids	4319-02-2
MWSmce387	1.83E+02	1.24E+02	1.84E+02	C8H8O5	[M-H]-	3-O-Methylgallic acid	Phenolic acids	Phenolic acids	3934-84-7
pme1299	1.83E+02	1.37E+02	1.84E+02	C8H8O5	[M-H]-	DL-3,4-Dihydroxymandelic acid	Phenolic acids	Phenolic acids	775-01-9
MWSmce230	1.83E+02	1.24E+02	1.84E+02	C8H8O5	[M-H]-	Methyl gallate	Phenolic acids	Phenolic acids	99-24-1
pmb1754	1.84E+02	1.25E+02	1.84E+02	C5H15NO4P+	[M]+	O-Phosphocholine	Alkaloids	Alkaloids	107-73-3
MWSslk083	1.83E+02	1.53E+02	1.84E+02	C9H12O4	[M-H]-	4-Hydroxy-3,5-dimethoxybenzyl alcohol	Others	Others	530-56-3
Lmfn006097	1.83E+02	1.39E+02	1.84E+02	C10H16O3	[M-H]-	Oleuropeic acid	Terpenoids	Monoterpenoids	5027-76-9
Wcdp005909	1.87E+02	1.32E+02	1.86E+02	C11H10N2O	[M+H]+	1,2,3,4-tetrahydronorharman-l-one	Alkaloids	Alkaloids	-
Lahp002608	1.88E+02	1.18E+02	1.87E+02	C11H9NO2	[M+H]+	3,5-Dihydro-2H-Furo[3,2-C]Quinolin-4-One*	Alkaloids	Quinoline alkaloids	-
Hmmp001310	1.88E+02	1.18E+02	1.87E+02	C11H9NO2	[M+H]+	3-Indoleacrylic acid*	Alkaloids	Plumerane	1204-06-4

Hmgp002327	1.88E+02	1.18E+02	1.87E+02	C11H9NO2	[M+H]+	3-amino-2-naphthoic acid*	Alkaloids	Alkaloids	-
Lahp001956	1.88E+02	1.18E+02	1.87E+02	C11H9O2N	[M+H]+	Pyran[3,4-b]indole-2-ketone	Alkaloids	Plumerane	-
Smcp001137	1.88E+02	1.18E+02	1.87E+02	C11H9NO2	[M+H]+	naphthisoxazol A*	Alkaloids	Alkaloids	-
MWSmce492	1.89E+02	1.71E+02	1.88E+02	C11H12N2O	[M+H]+	Vasicine	Alkaloids	Pyrrole alkaloids	7174-27-8
pmn001380	1.87E+02	1.69E+02	1.88E+02	C9H16O4	[M-H]-	Eucommiol	Others	Alcohol compounds	55930-44-4
pmf0348	1.87E+02	9.71E+01	1.88E+02	C10H20O3	[M-H]-	2,6-Dimethyl-7-octene-2,3,6-triol	Terpenoids	Monoterpenoids	73815-21-1
Ladp002935	1.90E+02	1.44E+02	1.89E+02	C10H7NO3	[M+H]+	3-quinolinecarboxylic acid	Alkaloids	Quinoline alkaloids	-
Zmtn001624	1.88E+02	1.44E+02	1.89E+02	C10H7NO3	[M-H]-	N-Acetylisatin	Alkaloids	Plumerane	574-17-4
pme2244	1.90E+02	1.18E+02	1.89E+02	C11H11NO2	[M+H]+	3-Indolepropionic acid	Alkaloids	Plumerane	830-96-6
Zmbn004194	1.89E+02	1.29E+02	1.90E+02	C8H14O5	[M-H]-	2-hydroxy-2-isobutylsuccinic acid	Others	Others	-

Wbjp001631	1.91E+02	1.73E+02	1.90E+02	C11H14N2O	[M+H]+	1,2,3,3a-tetrahydropyrrolo[2,1-b]quinazolin-4(9H)-ol	Alkaloids	Alkaloids	-
Lcsp001671	1.92E+02	1.46E+02	1.91E+02	C10H9NO3	[M+H]+	2-oxindole-3-acetic acid	Alkaloids	Plumerane	2971-31-5
mws0597	1.90E+02	1.46E+02	1.91E+02	C10H9NO3	[M-H]-	5-Hydroxyindole-3-acetic acid	Alkaloids	Plumerane	54-16-0
Cmpp003619	1.93E+02	1.05E+02	1.92E+02	C10H8O4	[M+H]+	6,7-Dihydroxy-4-methylcoumarin	Lignans and Coumarins	Coumarins	529-84-0
Zmjn003263	1.91E+02	8.10E+01	1.92E+02	C10H8O4	[M-H]-	6,8-Dihydroxy-3-methylisocoumarin	Lignans and Coumarins	Coumarins	1204-37-1
MWSHC20155	1.91E+02	1.76E+02	1.92E+02	C10H8O4	[M-H]-	Scopoletin (7-Hydroxy-6-methoxycoumarin)	Lignans and Coumarins	Coumarins	92-61-5
Lhhp120823	1.93E+02	1.05E+02	1.92E+02	C11H12O3	[M+H]+	Eugenyl formate	Others	Others	-
Wbjp001097	1.93E+02	1.48E+02	1.92E+02	C10H12N2O2	[M+H]+	3-(2-amino-1-hydroxyethyl)-1H-indol-5-ol	Alkaloids	Alkaloids	40979-78-0
Lhcp090203	1.93E+02	1.09E+02	1.92E+02	C13H20O	[M+H]+	α-Ionone	Terpenoids	Monoterpenoids	127-41-3
Wcdp001930	1.95E+02	1.63E+02	1.94E+02	C10H10O4	[M+H]+	3ξ-(1ξ-hydroxyethyl)-7-hydroxy-1-isobenzofuranone	Others	Others	-

mws0014	1.93E+02	1.34E+02	1.94E+02	C10H10O4	[M-H]-	Ferulic acid*	Phenolic acids	Phenolic acids	537-98-4
pme0422	1.93E+02	1.34E+02	1.94E+02	C10H10O4	[M-H]-	Isoferulic Acid*	Phenolic acids	Phenolic acids	25522-33-2
Zadp003164	1.95E+02	1.63E+02	1.94E+02	C11H14O3	[M+H]+	Dehydroloiolide	Terpenoids	Terpene	19355-58-9
MWSmce678	1.93E+02	1.08E+02	1.94E+02	C11H14O3	[M-H]-	Deoxyarbutin	Phenolic acids	Phenolic acids	53936-56-4
pmp001235	1.95E+02	7.91E+01	1.94E+02	C12H18O2	[M+H]+	Cnidilide	Others	Lactones	-
mws0612	1.95E+02	1.21E+02	1.96E+02	C10H12O4	[M-H]-	3,4-Dimethoxyphenyl acetic acid	Phenolic acids	Phenolic acids	93-40-3
pmf0319	1.95E+02	1.37E+02	1.96E+02	C10H12O4	[M-H]-	Acetosyringone	Others	Ketone compounds	2478-38-8
MWS20182	1.95E+02	1.36E+02	1.96E+02	C10H12O4	[M-H]-	Dihydroferulic Acid	Phenolic acids	Phenolic acids	1135-23-5
MWSprf048	1.95E+02	1.38E+02	1.96E+02	C12H20O2	[M-H]-	Linalyl Acetate	Others	Others	115-95-7
Zbqp000710	1.98E+02	1.54E+02	1.97E+02	C9H15N3O2	[M+H]+	3-(1H-imidazol-5-yl)-2-(trimethylammonio)propanoate	Alkaloids	Alkaloids	-

Lmxp00 0429	1.98 E+02	9.51 E+01	1.97E+02	C9H15N 3O2	[M+H]+	Hercynine	Alkaloids	Alkaloids	534-30-5
MWSm ce308	1.97 E+02	1.24 E+02	1.98E+02	C9H10O 5	[M-H]-	Gallic Acid Ethyl Ester; Ethyl gallate	Phenolic acids	Phenolic acids	831-61-8
mws002 7	1.97 E+02	1.23 E+02	1.98E+02	C9H10O 5	[M-H]-	Syringic acid	Phenolic acids	Phenolic acids	530-57-4
Wbjp00 2893	1.99 E+02	1.99 E+02	1.98E+02	C12H10 N2O	[M+H]+	Harmol	Alkaloids	Alkaloids	487-03-6
Zahn007 990	1.97 E+02	1.53 E+02	1.98E+02	C11H18 O3	[M-H]-	5-hydroxy-3,4-dimethyl-5-pentylfuran-2(5H)-one*	Others	Lactones	-
Lmxn00 8846	1.97 E+02	1.53 E+02	1.98E+02	C11H18 O3	[M-H]-	Hydroxydihydrobovolide*	Others	Lactones	6067-11-4
Hmln00 8119	1.97 E+02	1.53 E+02	1.98E+02	C11H18 O3	[M-H]-	Robinlin	Terpenoids	Monoterpenoids	-
MWSprf 155	2.01 E+02	1.60 E+02	2.00E+02	C12H12 N2O	[M+H]+	Harmalol; Harmidol; Harmolol;	Alkaloids	Alkaloids	525-57-5
Zbsn006 299	2.01 E+02	1.57 E+02	2.02E+02	C12H10 O3	[M-H]-	Plumbagin Methyl Ether; 5-Methoxy-2-Methyl-1,4-Naphthoquinone	Quinones	Quinones	22266-99-5
Wbjp00 1317	2.03 E+02	1.30 E+02	2.02E+02	C11H10 N2O2	[M+H]+	vasicinone	Alkaloids	Alkaloids	486-64-6

pmb0130	2.03 E+02	1.44 E+02	2.02E+02	C12H14 N2O	[M+H]+	Acetryptine	Alkaloids	Plumerane	3551-18-6
Wbjp001557	2.03 E+02	1.86 E+02	2.02E+02	C12H14 N2O	[M+H]+	tetrahydroharmol	Alkaloids	Alkaloids	17952-75-9
MWS1844	2.03 E+02	1.29 E+02	2.02E+02	C14H18 O	[M+H]+	2-Pentyl-3-phenyl-2-propenal	Others	Aldehyde compounds	122-40-7
mws0018	2.03 E+02	8.41 E+01	2.02E+02	C10H26 N4	[M+H]+	Spermine	Alkaloids	Alkaloids	71-44-3
MWSmce431	2.04 E+02	1.61 E+02	2.03E+02	C11H9N O3	[M+H]+	Quininic acid; 6-Methoxyquinoline-4-Carboxylic Acid	Alkaloids	Quinoline alkaloids	86-68-0
MWS5247	2.02 E+02	1.28 E+02	2.03E+02	C12H13 NO2	[M-H]-	Ethyl 3-Indoleacetate	Alkaloids	Plumerane	778-82-5
pme0224	2.02 E+02	1.58 E+02	2.03E+02	C12H13 NO2	[M-H]-	Indole-3-butyric acid	Alkaloids	Plumerane	133-32-4
MWSslk108	2.04 E+02	8.50 E+01	2.03E+02	C9H17N O4	[M+H]+	O-Acetyl-L-carnitine	Alkaloids	Alkaloids	3040-38-8
ZbWp001556	2.04 E+02	1.58 E+02	2.03E+02	C8H17N 3O3	[M+H]+	5-guanidino-2-(2-hydroxyethyl)pentanoic acid	Others	Others	-
Zbsn006549	2.03 E+02	1.59 E+02	2.04E+02	C11H8O 4	[M-H]-	8-Hydroxy-2-methoxy-1,4-naphthoquinone; 3-Methoxyjuglone	Quinones	Quinones	15254-76-9

Zahp011689	2.05E+02	1.49E+02	2.04E+02	C12H12O3	[M+H]+	2,2-dimethylchromene-6-carboxylic acid*	Others	Others	34818-56-9
Zmcp102201	2.05E+02	1.49E+02	2.04E+02	C12H12O3	[M+H]+	Senkyunolide B*	Others	Lactones	93236-67-0
Zmtn001464	2.04E+02	1.60E+02	2.05E+02	C10H7NO4	[M-H]-	4,8-Dihydroxyquinoline-2-carboxylic acid	Alkaloids	Quinoline alkaloids	59-00-7
Lmrn003201	2.04E+02	1.58E+02	2.05E+02	C11H11NO3	[M-H]-	Indole-3-lactic acid	Alkaloids	Plumerane	1821-52-9
pmb0818	2.06E+02	1.45E+02	2.05E+02	C11H11NO3	[M+H]+	Methoxyindoleacetic acid	Alkaloids	Plumerane	3471-31-6
HX1350	2.06E+02	1.46E+02	2.05E+02	C11H11NO3	[M+H]+	methyl 6-methoxy-3-indolecarbonate	Alkaloids	Alkaloids	-
pme1261	2.06E+02	7.61E+01	2.05E+02	C9H19NO4	[M+H]+	D-Panthenol	Others	Alcohol compounds	81-13-0
Lmbn013410	2.05E+02	1.89E+02	2.06E+02	C14H22O	[M-H]-	2,4-Di-Tert-Butylphenol	Others	Others	96-76-4
Lmln010063	2.05E+02	1.89E+02	2.06E+02	C14H22O	[M-H]-	2,6-Di-tert-butylphenol	Phenolic acids	Phenolic acids	128-39-2
MWS1877	2.05E+02	1.33E+02	2.06E+02	C14H22O	[M-H]-	4-tert-Octylphenol	Others	Others	140-66-9

Lmyn003131	2.06E+02	1.62E+02	2.07E+02	C10H9NO4	[M-H]-	Dioxindole-3-acetic acid	Alkaloids	Plumerane	-
Yamp002343	2.08E+02	1.20E+02	2.07E+02	C11H13NO3	[M+H]+	1-[2-(Furan-2-yl)-2-oxoethyl]piperidin-2-one	Alkaloids	Alkaloids	-
Zahp002452	2.08E+02	1.03E+02	2.07E+02	C12H17NO2	[M+H]+	phenylalanine betaine	Alkaloids	Alkaloids	56755-22-7
ZbWn003583	2.07E+02	8.00E+01	2.08E+02	C10H8O3S	[M-H]-	1-NAPHTHALENESULFONIC ACID	Others	Others	85-47-2
MA10107783	2.07E+02	1.19E+02	2.08E+02	C10H8O5	[M-H]-	3-[(1-Carboxyvinyl)oxy]benzoic acid	Phenolic acids	Phenolic acids	16929-37-6
MWSmce332	2.09E+02	1.91E+02	2.08E+02	C11H12O4	[M+H]+	3,4-Dimethoxycinnamic acid	Phenolic acids	Phenolic acids	2316-26-9
mws2184	2.07E+02	1.35E+02	2.08E+02	C11H12O4	[M-H]-	Ethyl caffeate	Phenolic acids	Phenolic acids	102-37-4
Lmtp004382	2.09E+02	1.49E+02	2.08E+02	C11H12O4	[M+H]+	Sinapinaldehyde	Others	Aldehyde compounds	4206-58-0
MWSmce294	2.09E+02	1.53E+02	2.08E+02	C12H16O3	[M+H]+	Elemicin	Others	Others	487-11-6
Hmcp003783	2.11E+02	1.93E+02	2.10E+02	C13H10N2O	[M+H]+	1-Acetyl-β-carboline	Alkaloids	Plumerane	-

Lmhp003958	2.11E+02	7.01E+01	2.10E+02	C11H18N2O2	[M+H]+	Deoxymutaaspergillic acid	Alkaloids	Alkaloids	17004-73-8
Hmlp008487	2.11E+02	8.11E+01	2.10E+02	C13H22O2	[M+H]+	Blumenol C	Terpenoids	Monoterpenoids	36151-02-7
MWS5159	2.12E+02	1.68E+02	2.11E+02	C9H9NOS2	[M+H]+	2-(2-Benzothiazolylthio)ethanol	Alkaloids	Alkaloids	4665-63-8
Lasp008777	2.12E+02	1.84E+02	2.11E+02	C13H9NO2	[M+H]+	1-hydroxyacridone	Alkaloids	Quinorisidine alkaloids	65582-54-9
Wccp001401	2.12E+02	1.94E+02	2.11E+02	C10H13NO4	[M+H]+	4-[2-formyl-5-(hydroxymethyl)pyrrol-1-yl]butanoic acid	Alkaloids	Alkaloids	-
Hmcp001824	2.13E+02	1.67E+02	2.12E+02	C12H8N2O2	[M+H]+	Ailanindole	Alkaloids	Plumerane	159903-51-2
Wcfn002460	2.11E+02	1.48E+02	2.12E+02	C10H12O5	[M-H]-	2,3-Dihydroxy-1-(4'-hydroxy-3'-methoxyphenyl)-propan-1-one	Others	Others	-
Cmlp002967	2.13E+02	1.49E+02	2.12E+02	C10H12O5	[M+H]+	Gentiolactone	Terpenoids	Monoterpenoids	-
mws1512	2.11E+02	1.24E+02	2.12E+02	C10H12O5	[M-H]-	Propyl gallate	Phenolic acids	Phenolic acids	121-79-9
Wasn003547	2.11E+02	9.70E+01	2.12E+02	C7H16O5S	[M-H]-	Heptane-1,7-diol sulfate	Others	Others	-

mws1509	2.15E+02	1.21E+02	2.14E+02	C13H10O3	[M+H]+	Phenyl salicylate	Phenolic acids	Phenolic acids	118-55-8
Lazn001573	2.13E+02	1.50E+02	2.14E+02	C10H14O5	[M-H]-	Erythro-Guaiacylglycerol	Phenolic acids	Phenolic acids	-
Qajp007826	2.16E+02	1.31E+02	2.15E+02	C14H17NO	[M+H]+	1-Cinnamoylpiperidine	Alkaloids	Piperidine alkaloids	5422-81-1
Wcdp003967	2.18E+02	8.81E+01	2.17E+02	C12H27NO2	[M+H]+	Deacetylclavaminol H	Others	Others	-
Lmcp003522	2.19E+02	1.73E+02	2.18E+02	C12H10O4	[M+H]+	Baldrinal	Terpenoids	Monoterpenoids	18234-46-3
Wbjp001606	2.19E+02	1.76E+02	2.18E+02	C12H14N2O2	[M+H]+	3-(4-aminobut-1-en-1-yl)-4,7-dihydro-1-indole-5,6-dione	Alkaloids	Alkaloids	-
mws0677	2.19E+02	1.60E+02	2.18E+02	C12H14N2O2	[M+H]+	N-Acetyl-5-hydroxytryptamine	Alkaloids	Plumerane	1210-83-9
Lasp001037	2.19E+02	7.21E+01	2.18E+02	C13H18N2O	[M+H]+	N-ethylcytisine	Alkaloids	Piperidine alkaloids	-
Lhcp120603	2.19E+02	2.03E+02	2.18E+02	C15H22O	[M+H]+	8,12-Epoxy-eremophila-9,11(13)-diene*	Terpenoids	Sesquiterpenoids	-
MWSslk234	2.19E+02	1.21E+02	2.18E+02	C15H22O	[M+H]+	Aristolone	Terpenoids	Sesquiterpenoids	25274-27-5

Lhfp110832	2.19E+02	2.03E+02	2.18E+02	C15H22O	[M+H]+	Furanoeremophilane*	Terpenoids	Sesquiterpenoids	6750-13-6
Lskp211392	2.19E+02	1.45E+02	2.18E+02	C15H22O	[M+H]+	Libocedrine B	Terpenoids	Sesquiterpenoids	-
MWSmce096	2.19E+02	8.11E+01	2.18E+02	C15H22O	[M+H]+	Nootkatone*	Terpenoids	Sesquiterpenoids	4674-50-4
zjbp111643	2.19E+02	8.11E+01	2.18E+02	C15H22O	[M+H]+	neopetasane*	Terpenoids	Sesquiterpenoids	-
Wbjp002330	2.21E+02	1.75E+02	2.20E+02	C11H12N2O3	[M+H]+	1,3-dihydroxy-2,3,3a,4-tetrahydropyrrolo[2,1-b]quinazolin-9(1H)-one	Alkaloids	Alkaloids	-
Wbjp001169	2.21E+02	1.30E+02	2.20E+02	C11H12N2O3	[M+H]+	o-Carboxy-5-hydroxytryptamine	Alkaloids	Alkaloids	-
Zjyp102940	2.21E+02	1.19E+02	2.20E+02	C14H20O2	[M+H]+	Oxyphyllone C	Terpenoids	Sesquiterpenoids	-
HX1224	2.21E+02	1.47E+02	2.20E+02	C15H24O	[M+H]+	2-methyl-2-vinyl-3-isopropenyl-5-isopropylidene cyclohexanol*	Terpenoids	Monoterpenoids	-
Qmjp080607	2.21E+02	1.47E+02	2.20E+02	C15H24O	[M+H]+	Acoragermacrone	Terpenoids	Sesquiterpenoids	50281-45-3
Lhfp110340	2.21E+02	1.19E+02	2.20E+02	C15H24O	[M+H]+	Eremofukinone	Terpenoids	Sesquiterpenoids	-

Lmtp005226	2.21E+02	1.05E+02	2.20E+02	C15H24O	[M+H]+	Santalol	Terpenoids	Sesquiterpenoids	11031-45-1
Zmcp102205	2.21E+02	1.19E+02	2.20E+02	C15H24O	[M+H]+	Spathulenol	Terpenoids	Sesquiterpenoids	6750-60-3
Sacp008256	2.21E+02	1.47E+02	2.20E+02	C15H24O	[M+H]+	isoshyobunone*	Terpenoids	Sesquiterpenoids	21698-46-4
Ymmg000003	2.21E+02	8.11E+01	2.20E+02	C15H24O	[M+H]+	rosacorenol	Terpenoids	Sesquiterpenoids	-
Xmln005368	2.21E+02	1.77E+02	2.22E+02	C13H18O3	[M-H]-	Dehydrovomifoliol	Terpenoids	Monoterpenoids	15764-81-5
Wbmn003185	2.21E+02	2.21E+02	2.22E+02	C14H22O2	[M-H]-	Kobusone	Terpenoids	Sesquiterpenoids	24173-71-5
Hmcp003852	2.23E+02	2.07E+02	2.22E+02	C15H26O	[M+H]+	Elemol	Terpenoids	Sesquiterpenoids	639-99-6
Zmln002252	2.23E+02	1.49E+02	2.24E+02	C10H8O6	[M-H]-	Sideretin (5,7,8-Trihydroxy-6-methoxycoumarin)	Lignans and Coumarins	Coumarins	-
MWS1872	2.25E+02	1.05E+02	2.24E+02	C14H8O3	[M+H]+	1-Hydroxyanthraquinone	Quinones	Anthraquinone	129-43-1
MWSN0154	2.23E+02	1.17E+02	2.24E+02	C15H12O2	[M-H]-	4'-Hydroxychalcone	Flavonoids	Chalcones	2657-25-2

MWSslk101	2.25E+02	1.47E+02	2.24E+02	C15H12O2	[M+H]+	4-Hydroxychalcone	Flavonoids	Chalcones	20426-12-4
Wcgn008207	2.23E+02	1.79E+02	2.24E+02	C12H16O4	[M-H]-	Aspidinol	Others	Others	519-40-4
Lmdn006074	2.23E+02	1.79E+02	2.24E+02	C12H16O4	[M-H]-	Senkyunolide I	Others	Lactones	94596-28-8
HX1300	2.25E+02	1.21E+02	2.24E+02	C13H20O3	[M+H]+	2-(4-methylpent-3-enyl)-6-oxohept-2-enoic acid	Terpenoids	Ditepenoids	-
mws1429	2.23E+02	1.61E+02	2.24E+02	C13H20O3	[M-H]-	Vomifoliol (Blumenol A)	Terpenoids	Monoterpenoids	189351-15-3
Lssp210412	2.25E+02	1.00E+02	2.24E+02	C13H24N2O	[M+H]+	Cuscohygrine	Alkaloids	Pyrrole alkaloids	454-14-8
pmp001296	2.26E+02	6.00E+01	2.25E+02	C11H15NO4	[M+H]+	Radicamine B	Alkaloids	Pyrrole alkaloids	431981-75-8
Wbsp000191	2.26E+02	1.80E+02	2.25E+02	C12H23N3O	[M+H]+	3,7,11-trimethyl-2-oxa-6,10,13-triazatricyclo[7.3.1.05,13]tridecane	Alkaloids	Alkaloids	-
MWSHC2029	2.27E+02	1.67E+02	2.26E+02	C13H10N2O2	[M+H]+	Harman-3-carboxylic acid	Alkaloids	Plumerane	22329-38-0
HJAP051	2.27E+02	1.81E+02	2.26E+02	C12H18O4	[M+H]+	3-(3,4,5-Trimethoxyphenyl)propan-1-ol	Others	Alcohol compounds	-

pmp001294	2.28E+02	2.10E+02	2.27E+02	C13H25NO2	[M+H]+	N-Methyl-2-(2-hydroxypropyl)-6-(2-hydroxybutyl)-Δ3-piperideine	Alkaloids	Piperidine alkaloids	-
Zahp011321	2.28E+02	2.28E+02	2.27E+02	C14H29NO	[M+H]+	N-Isobutyl Decanamide	Alkaloids	Alkaloids	73785-31-6
mws0021	2.27E+02	1.85E+02	2.28E+02	C14H12O3	[M-H]-	Resveratrol	Others	Stilbene	501-36-0
HX1151	2.29E+02	1.07E+02	2.28E+02	C14H12O3	[M+H]+	Trans resveratrol	Others	Stilbene	-
Lmhp003426	2.29E+02	1.83E+02	2.28E+02	C13H12N2O2	[M+H]+	Aspernigrin A	Alkaloids	Pyridine alkaloids	773855-60-0
Zamn006847	2.27E+02	1.83E+02	2.28E+02	C12H20O4	[M-H]-	glycoric acid	Others	Others	-
Wcdp004275	2.30E+02	5.71E+01	2.29E+02	C14H31NO	[M+H]+	Lauramine oxide	Others	Others	1643-20-5
MWSHC2027	2.31E+02	1.58E+02	2.30E+02	C13H14N2O2	[M+H]+	(1R,3S)-1-Methyl-1,2,3,4-tetrahydro-β-carboline-3-carboxylic acid*	Alkaloids	Plumerane	42438-72-2
MWSHC2028	2.31E+02	1.58E+02	2.30E+02	C13H14N2O2	[M+H]+	(1S,3S)-1-Methyl-1,2,3,4-tetrahydro-β-carboline-3-carboxylic acid*	Alkaloids	Plumerane	40678-46-4
Wbjp002156	2.31E+02	1.85E+02	2.30E+02	C13H14N2O2	[M+H]+	2-[(9R)-1,2,3,9-tetrahydropyrrolo[2,1-b]quinazolin-9-yl]acetic acid	Alkaloids	Alkaloids	-

MWS1113	2.32E+02	5.70E+01	2.31E+02	C11H21NO4	[M+H]+	Isobutyryl carnitine	Alkaloids	Alkaloids	25518-49-4
Lmhn005886	2.31E+02	1.87E+02	2.32E+02	C13H12O4	[M-H]-	5-(2'-Hydroxy-6'-methyl phenyl)-3-methylfuran-2-carboxylic acid	Others	Others	-
Zayp007475	2.33E+02	1.59E+02	2.32E+02	C13H12O4	[M+H]+	dihydrocoriandrin	Lignans and Coumarins	Coumarins	116383-99-4
mws0019N	2.31E+02	1.45E+02	2.32E+02	C13H16N2O2	[M-H]-	Melatonin (N-Acetyl-5-methoxytryptamine)	Alkaloids	Plumerane	73-31-4
Qmyp122107	2.33E+02	1.05E+02	2.32E+02	C15H20O2	[M+H]+	3,5,11(13)-Trieneudesma-13-oic acid	Terpenoids	Sesquiterpenoids	-
Zmdn004719	2.33E+02	1.89E+02	2.34E+02	C13H14O4	[M-H]-	2-(2'-hydroxypropyl)-5-methyl-7-hydroxychromone	Others	Chromone	-
Zjyp102909	2.35E+02	1.19E+02	2.34E+02	C15H22O2	[M+H]+	11α-hydroxy-3-oxo-4(5),6(7)-diene-eudesman-12-ol	Terpenoids	Sesquiterpenoids	-
Zjyp102905	2.35E+02	1.19E+02	2.34E+02	C15H22O2	[M+H]+	7-epi-teucrenone	Terpenoids	Sesquiterpenoids	-
Lhgp080602	2.35E+02	6.71E+01	2.34E+02	C15H22O2	[M+H]+	8-Keto-9(10)-α-patchoulene-4α-ol	Terpenoids	Sesquiterpenoids	-
zjbp111604	2.35E+02	1.33E+02	2.34E+02	C15H22O2	[M+H]+	8α-hydroxyeudesma-3,11(13)-dien-14-al	Terpenoids	Sesquiterpenoids	-

zjbp111602	2.35E+02	1.05E+02	2.34E+02	C15H22O2	[M+H]+	9β-hydroxyeudesma-4,11(13)-dien-12-al	Terpenoids	Sesquiterpenoids	-
ML10174588	2.33E+02	2.33E+02	2.34E+02	C15H22O2	[M-H]-	Confertifoline	Terpenoids	Sesquiterpenoids	1811-23-0
Qatp009393	2.35E+02	1.05E+02	2.34E+02	C15H22O2	[M+H]+	Dihydrocostunolide	Terpenoids	Sesquiterpenoids	2225-79-8
Lhsp101904	2.35E+02	1.79E+02	2.34E+02	C15H22O2	[M+H]+	polygodial	Terpenoids	Sesquiterpenoids	6754-20-7
Wbsp000037	2.36E+02	5.80E+01	2.35E+02	C14H21NO2	[M+H]+	1-[3-(4-methyl-6-oxocyclohex-1-en-1-yl)propyl]pyrrolidin-2-one	Others	Ketone compounds	-
Hmcp002554	2.37E+02	2.09E+02	2.36E+02	C14H8N2O2	[M+H]+	1-Hydroxycanthin-6-one*	Alkaloids	Plumerane	80787-59-3
MWStz311	2.37E+02	2.09E+02	2.36E+02	C14H8N2O2	[M+H]+	10-Hydroxycanthin-6-One*	Alkaloids	Plumerane	86293-41-6
MWStz295	2.37E+02	2.09E+02	2.36E+02	C14H8N2O2	[M+H]+	11-Hydroxycanthin-6-One; Amalorin*	Alkaloids	Plumerane	75969-83-4
MWSHC20196	2.37E+02	8.11E+01	2.36E+02	C15H24O2	[M+H]+	10α-Hydroxycadin-4-en-15-al	Terpenoids	Sesquiterpenoids	398147-37-0
MWSslk086	2.37E+02	8.11E+01	2.36E+02	C16H28O	[M+H]+	Ambroxide	Terpenoids	Ditepenoids	6790-58-5

MWS20166	2.37E+02	2.09E+02	2.38E+02	C15H10O3	[M-H]-	2-hydroxy-3-methyl-anthraquinone	Quinones	Anthraquinone	-
MWSHY0149	2.39E+02	1.37E+02	2.38E+02	C15H10O3	[M+H]+	5-Hydroxyflavone	Flavonoids	Flavones	491-78-1
Lmyn000160	2.37E+02	8.70E+01	2.38E+02	C8H14O8	[M-H]-	Mucic acid Dimethyl Ester	Phenolic acids	Phenolic acids	-
pmn001320	2.37E+02	1.63E+02	2.38E+02	C12H14O5	[M-H]-	1-O-p-Cumaroylglycerol	Phenolic acids	Phenolic acids	106055-11-2
pmn001669	2.37E+02	2.07E+02	2.38E+02	C12H14O5	[M-H]-	Methyl sinapate	Phenolic acids	Phenolic acids	20733-94-2
Zasp001180	2.39E+02	1.10E+02	2.38E+02	C11H14N2O4	[M+H]+	γ-L-glutaminy-4-hydroxybenzene	Alkaloids	Alkaloids	30382-24-2
Smdp002817	2.40E+02	1.40E+02	2.39E+02	C13H21NO3	[M+H]+	Farfugine	Alkaloids	Pyrrole alkaloids	86194-08-3
pmp000476	2.40E+02	5.71E+01	2.39E+02	C14H25NO2	[M+H]+	N-Isobutyl-4,5-epoxy-2E-decaenamide	Alkaloids	Alkaloids	-
Wabn005430	2.39E+02	1.25E+02	2.40E+02	C11H12O6	[M-H]-	Hydroxyplumbagic acid	Phenolic acids	Phenolic acids	-
Zmdn011161	2.39E+02	2.23E+02	2.40E+02	C15H12O3	[M-H]-	Chrysophanol-9-anthrone	Quinones	Anthraquinone	491-58-7

MWStz 201	2.41 E+02	2.23 E+02	2.40E+02	C14H12 N2O2	[M+H]+	β-Carboline-1-propanoic acid	Alkaloids	Plumerane	89915-39-9
Lmzn00 1925	2.39 E+02	1.95 E+02	2.40E+02	C12H16 O5	[M-H]-	3,4'-Dihydroxy-3'-methoxybenzenepentanoic acid	Phenolic acids	Phenolic acids	-
Hmln01 3535	2.39 E+02	2.23 E+02	2.40E+02	C15H28 O2	[M-H]-	Cryptomeridiol	Terpenoids	Sesquiterpenoids	4666-84-6
Wbtn00 6160	2.41 E+02	1.95 E+02	2.42E+02	C14H10 O4	[M-H]-	5-hydroxy-2-methoxyxanthen-9-one	Flavonoids	Other Flavonoids	141766-17-8
Wbjp00 3795	2.43 E+02	1.97 E+02	2.42E+02	C13H10 N2O3	[M+H]+	7-methoxy-9H-β-carboline-1-carboxylic acid	Alkaloids	Alkaloids	17019-05-5
mws138 3	2.43 E+02	1.45 E+02	2.42E+02	C12H10 N4O2	[M+H]+	Lumichrome	Alkaloids	Alkaloids	1086-80-2
Lmmn0 05696	2.43 E+02	2.28 E+02	2.44E+02	C15H16 O3	[M-H]-	Desoxyhemigossypol	Phenolic acids	Phenolic acids	57765-65-8
MWSm ce509	2.45 E+02	1.16 E+02	2.46E+02	C17H14 N2	[M-H]-	3,3'-Diindolylmethane	Alkaloids	Plumerane	1968-05-4
pmp000 912	2.47 E+02	2.29 E+02	2.46E+02	C15H18 O3	[M+H]+	Hydroxyindesteolide	Others	Lactones	-
MWSm ce062	2.47 E+02	1.31 E+02	2.46E+02	C15H18 O3	[M+H]+	Xanthatin	Terpenoids	Sesquiterpenoids	26791-73-1

Smjp001498	2.47E+02	1.46E+02	2.46E+02	C14H18N2O2	[M+H]+	Hypaphorine	Alkaloids	Plumerane	487-58-1
zjbp111639	2.49E+02	1.85E+02	2.48E+02	C15H20O3	[M+H]+	7βH-guaia-1(10)-en-12,8β-olide	Terpenoids	Sesquiterpenoids	-
Hmcp003285	2.49E+02	1.85E+02	2.48E+02	C15H20O3	[M+H]+	Santamarin	Terpenoids	Sesquiterpenoids	4290-13-5
Hmsp005892	2.49E+02	1.37E+02	2.48E+02	C15H20O3	[M+H]+	[4]-Shogaol	Phenolic acids	Phenolic acids	-
Sadn008165	2.47E+02	1.61E+02	2.48E+02	C15H20O3	[M-H]-	β-Cannabispiranol	Terpenoids	Sesquiterpenoids	64052-90-0
Wcgp011192	2.49E+02	1.93E+02	2.48E+02	C16H24O2	[M+H]+	5-Hydroxy-8,8-dimethyl-1,3,4,4a,4b,5,6,7,8a,9-decahydrophenanthren-2-one	Others	Others	-
pmb0323n	2.49E+02	1.35E+02	2.50E+02	C13H18N2O3	[M-H]-	N-Caffeoylputrescine	Alkaloids	Phenolamine	29554-26-5
Lmjn006711	2.49E+02	2.05E+02	2.50E+02	C15H22O3	[M-H]-	4,5-Epoxyartemisinic Acid	Terpenoids	Sesquiterpenoids	92466-31-4
Jmcn006879	2.49E+02	2.03E+02	2.50E+02	C15H22O3	[M-H]-	5β-Hydroxycostic acid	Terpenoids	Sesquiterpenoids	-
Lahp008617	2.52E+02	1.31E+02	2.51E+02	C17H17NO	[M+H]+	N-2-Phenylethyl-cinnamamide	Alkaloids	Phenolamine	-

MWSmce413	2.51E+02	2.07E+02	2.52E+02	C15H8O4	[M-H]-	Anthraquinone-2-carboxylic acid	Quinones	Anthraquinone	117-78-2
Zasn001080	2.51E+02	1.22E+02	2.52E+02	C11H12N2O5	[M-H]-	γ-L-glutaminyl-3,4-benzoquinone	Alkaloids	Alkaloids	30382-25-3
Xmdn001002	2.51E+02	1.64E+02	2.52E+02	C17H16O2	[M-H]-	Trans-hinokiresinol	Lignans and Coumarins	Lignans	17676-24-3
Lmmp001410	2.53E+02	1.65E+02	2.52E+02	C13H20N2O3	[M+H]+	Dihydrocaffeoylputrescine	Alkaloids	Phenolamine	-
Qmqp102535	2.53E+02	1.21E+02	2.52E+02	C15H24O3	[M+H]+	Ilicic acid	Terpenoids	Sesquiterpenoids	4586-68-9
Wcsn009782	2.51E+02	2.51E+02	2.52E+02	C16H28O2	[M-H]-	12-hydroxy-2,6,10-trimethyltrideca-2,5-dien-4-one	Others	Others	-
Lmhn003373	2.53E+02	1.37E+02	2.54E+02	C11H10O7	[M-H]-	4-Hydroxybenzoylmalic acid	Phenolic acids	Phenolic acids	-
Lmhn002629	2.53E+02	1.21E+02	2.54E+02	C11H10O7	[M-H]-	Benzoyltartaric acid	Phenolic acids	Phenolic acids	-
MWSslk183	2.55E+02	1.81E+02	2.54E+02	C15H10O4	[M+H]+	7,8-Dihydroxy-4-phenylcoumarin	Lignans and Coumarins	Coumarins	842-01-3
mws1176	2.53E+02	2.25E+02	2.54E+02	C15H10O4	[M-H]-	Anhydroglycinol(3,9-Dihydroxypterocarpen)	Others	Alcohol compounds	67685-22-7

MWSN0003	2.53E+02	2.09E+02	2.54E+02	C15H10O4	[M-H]-	Chrysin	Flavonoids	Flavones	480-40-0
MWSN0004	2.53E+02	2.08E+02	2.54E+02	C15H10O4	[M-H]-	Daidzein	Flavonoids	Isoflavones	486-66-8
ZbWn004461	2.53E+02	9.70E+01	2.54E+02	C9H18O6S	[M-H]-	2-(2-pent-4-enoxyethoxy)ethyl hydrogen sulfate	Others	Others	-
Zasn000905	2.53E+02	1.24E+02	2.54E+02	C11H14N2O5	[M-H]-	γ-L-glutaminy-3,4-dihydroxybenzene(GDHB)	Alkaloids	Alkaloids	-
Wcdp010864	2.56E+02	8.81E+01	2.55E+02	C16H33NO	[M+H]+	Hexadecanamide	Alkaloids	Alkaloids	629-54-9
Zmdn006929	2.55E+02	2.13E+02	2.56E+02	C15H12O4	[M-H]-	Aloe-Emodin-9-Anthrone	Quinones	Anthraquinone	6247-99-0
mws0789	2.55E+02	1.51E+02	2.56E+02	C15H12O4	[M-H]-	Pinocembrin (Dihydrochrysin)	Flavonoids	Flavanones	480-39-7
Wagp009130	2.59E+02	1.44E+02	2.58E+02	C16H22N2O	[M+H]+	N-Hexanoyl Tryptamine	Alkaloids	Plumerane	-
Wmjn000315	2.59E+02	2.59E+02	2.60E+02	C13H8O6	[M-H]-	1,3,6,7-Tetrahydroxyxanthone	Flavonoids	Flavones	-
Wbtn006411	2.59E+02	2.15E+02	2.60E+02	C13H8O6	[M-H]-	Urolithin D	Flavonoids	Other Flavonoids	131086-98-1

pmp000626	2.61E+02	2.43E+02	2.60E+02	C15H20N2O2	[M+H]+	Baptifoline	Alkaloids	Quinorisidine alkaloids	732-50-3
Wcdp010180	2.61E+02	1.21E+02	2.60E+02	C18H28O	[M+H]+	9,11,13,15-Octadecatetraenal	Others	Aldehyde compounds	148408-16-6
MWSslk045	2.63E+02	2.35E+02	2.62E+02	C16H10N2O2	[M+H]+	Indigo	Alkaloids	Plumerane	482-89-3
Hmmn006338	2.61E+02	2.17E+02	2.62E+02	C15H18O4	[M-H]-	Armexifolin	Terpenoids	Sesquiterpenoids	64929-15-3
Wagp007852	2.63E+02	1.31E+02	2.62E+02	C15H18O4	[M+H]+	Osthol hydrate	Lignans and Coumarins	Coumarins	69219-24-5
Zmgp005232	2.63E+02	9.11E+01	2.62E+02	C19H18O	[M+H]+	1,7-diphenyl-4,6-diene-3-heptanone	Others	Ketone compounds	-
Wcdp010708	2.63E+02	8.11E+01	2.62E+02	C18H30O	[M+H]+	Octadeca-2,9,12,15-tetraen-1-ol	Others	Others	-
Lmtn004049	2.63E+02	2.04E+02	2.64E+02	C15H20O4	[M-H]-	Abscisic acid	Terpenoids	Sesquiterpenoids	21293-29-8
Lmtn004697	2.63E+02	2.19E+02	2.64E+02	C15H20O4	[M-H]-	Ambrosic acid	Terpenoids	Sesquiterpenoids	6617-14-7
Hmcn007243	2.63E+02	2.19E+02	2.64E+02	C15H20O4	[M-H]-	Artemisiifolin	Terpenoids	Sesquiterpenoids	26931-87-3

pmp000917	2.47E+02	2.29E+02	2.64E+02	C15H20O4	[M-H2O+H]+	Lindenenol K	Terpenoids	Sesquiterpenoids	-
Latn006216	2.65E+02	1.17E+02	2.66E+02	C17H14O3	[M-H]-	Asparenydiol	Lignans and Coumarins	Lignans	166762-98-7
Cmpn007831	2.65E+02	2.21E+02	2.66E+02	C15H22O4	[M-H]-	Arsanin	Terpenoids	Sesquiterpenoids	41135-04-0
Lmjn008379	2.65E+02	2.21E+02	2.66E+02	C15H22O4	[M-H]-	Chloranthalic acid	Terpenoids	Sesquiterpenoids	-
Lmxn006699	2.65E+02	1.23E+02	2.66E+02	C15H22O4	[M-H]-	Deacetylinulicin(Britannilactone)	Terpenoids	Sesquiterpenoids	33620-72-3
Hmsp004517	2.67E+02	1.37E+02	2.66E+02	C15H22O4	[M+H]+	[4]-Gingerol	Phenolic acids	Phenolic acids	77398-90-4
Zmgp004207	2.67E+02	2.49E+02	2.66E+02	C19H22O	[M+H]+	1,7-Diphenyl-2-hydroxy-heptene	Others	Others	-
Zbdp001892	2.68E+02	1.36E+02	2.67E+02	C16H13NO3	[M+H]+	7-Methoxy-3-[1-(3-pyridyl)methylidene]-4-chromanone	Flavonoids	Other Flavonoids	-
Lacp001431	2.68E+02	1.36E+02	2.67E+02	C14H21NO4	[M+H]+	N-(3-hydroxy-4-methoxyphenethyl)-4-hydroxybutanamide	Alkaloids	Alkaloids	-
MWS20177	2.67E+02	2.23E+02	2.68E+02	C15H8O5	[M-H]-	5-hydroxy-anthraquinone-2-carboxylic acid	Quinones	Anthraquinone	-

mws1405	2.67E+02	2.11E+02	2.68E+02	C15H8O5	[M-H]-	Coumestrol	Lignans and Coumarins	Coumarins	479-13-0
Zmyn003693	2.67E+02	2.52E+02	2.68E+02	C16H12O4	[M-H]-	6-Hydroxy-2'-methoxyflavone	Flavonoids	Flavones	61546-59-6
MWS20167	2.67E+02	2.52E+02	2.68E+02	C16H12O4	[M-H]-	Rubiadin-1-Methyl Ether	Quinones	Anthraquinone	7460-43-7
MWSHY0192	2.69E+02	2.26E+02	2.68E+02	C16H12O4	[M+H]+	Tectochrysin	Flavonoids	Flavones	520-28-5
Lhmp122201	2.69E+02	2.53E+02	2.68E+02	C16H12O4	[M+H]+	dalbergin	Lignans and Coumarins	Coumarins	482-83-7
Wcjp001032	2.69E+02	1.37E+02	2.68E+02	C10H12N4O5	[M+H]+	9-[(2S,3S,4S,5R)-3,4-dihydroxy-5-(hydroxymethyl)oxolan-2-yl]-1H-purin-6-one	Alkaloids	Alkaloids	-
Sadp007533	2.69E+02	2.23E+02	2.68E+02	C17H16O3	[M+H]+	5-formyl-2,6-dihydroxy-1,7-dimethyl-9,10-dihydrophenanthrene	Terpenoids	Ditepenoids	-
Sadp008019	2.69E+02	2.23E+02	2.68E+02	C17H16O3	[M+H]+	5-formyl-2,7-dihydroxy-1,6-dimethyl-9,10-dihydrophenanthrene	Terpenoids	Ditepenoids	-
Lahp007087	2.70E+02	1.05E+02	2.69E+02	C17H19NO2	[M+H]+	N-Benzoyltyramine	Alkaloids	Phenolamine	-
MWSN0178	2.69E+02	2.25E+02	2.70E+02	C15H10O5	[M-H]-	2'-Hydroxydaidzein	Flavonoids	Isoflavones	7678-85-5

pmp000344	2.71E+02	2.43E+02	2.70E+02	C15H10O5	[M+H]+	3',4',7-Trihydroxyflavone	Flavonoids	Flavones	2150-11-0
Lmyn004949	2.69E+02	1.33E+02	2.70E+02	C15H10O5	[M-H]-	3,7,3'-Trihydroxyflavone	Flavonoids	Flavones	-
Wbkp005691	2.71E+02	2.25E+02	2.70E+02	C15H14N2O3	[M+H]+	3-(4-methoxy-9H-pyrido[3,4-b]indol-1-yl)propanoic acid	Alkaloids	Alkaloids	-
Sadn007155	2.69E+02	2.25E+02	2.70E+02	C17H18O3	[M-H]-	4-(hydroxymethyl)-1,8-dimethyl-9,10-dihydrophenanthrene-2,7-diol	Terpenoids	Ditepenoids	-
Zblp001009	2.72E+02	1.10E+02	2.71E+02	C12H17NO6	[M+H]+	3-pyridine-methanol-O-β-D-glucopyranosyl	Alkaloids	Pyridine alkaloids	-
pmp000483	2.72E+02	2.01E+02	2.71E+02	C16H17NO3	[M+H]+	1-[1-O-5(3,4-methylenedioxyphenyl)-2E,4E-pentadienyl]-pyrrolidine	Alkaloids	Pyrrole alkaloids	-
Wcdp006912	2.74E+02	8.81E+01	2.73E+02	C16H35NO2	[M+H]+	Lauryldiethanolamine	Others	Others	1541-67-9
Lmjp101205	2.75E+02	2.19E+02	2.74E+02	C16H18O4	[M+H]+	Coumurrayin	Lignans and Coumarins	Coumarins	-
Wcgp010745	2.75E+02	1.49E+02	2.74E+02	C18H26O2	[M+H]+	3-(4-hydroxycyclohex-1-en-1-yl)-4a,5-dimethyl-3,4,4a,5,6,8a-hexahydronaphthalen-1(2H)-one	Others	Others	-
Wbhp009515	2.75E+02	2.75E+02	2.74E+02	C18H26O2	[M+H]+	Tagalsin Q	Terpenoids	Terpene	-

Wbmp0 07068	2.77 E+02	1.75 E+02	2.76E+02	C15H16 O5	[M+H]+	(2s,3r)-8-hydroxy-3-(hydroxymethyl)-2,3,9-trimethyl-2h-furo[3,2-c]chromen-4-one	Others	Others	-
Qmjp08 0402	2.77 E+02	2.35 E+02	2.76E+02	C18H28 O2	[M+H]+	15,16-Bisnor-13-oxo-8(17),11-labdadien-19-ol	Terpenoids	Sesquiterpenoids	-
Wcgp01 0223	2.77 E+02	9.31 E+01	2.76E+02	C18H28 O2	[M+H]+	3-(Cyclohexen-1-yl)-2-hydroxy-4a,5-dimethyl-2,3,4,5,6,7,8,8a-octahydronaphthalen-1-one	Others	Others	-
Wbmp0 10456	2.77 E+02	9.31 E+01	2.76E+02	C18H28 O2	[M+H]+	Macrophypene B	Terpenoids	Ditepenoids	-
Wcsn00 9515	2.75 E+02	2.31 E+02	2.76E+02	C18H28 O2	[M-H]-	Methyl (2E,4E,9E)-5,9,13-trimethyltetradeca-2,4,9,12-tetraenoate	Others	Others	-
Latp009 929	2.79 E+02	1.49 E+02	2.78E+02	C15H18 O5	[M+H]+	6α,10α-Dihydroxy-1-oxoeremophila-7(11),8(9)-dien-12,8-olide	Terpenoids	Sesquiterpenoids	-
mws103 8	2.77 E+02	1.46 E+02	2.78E+02	C11H22 N2O4S	[M-H]-	Pantetheine	Alkaloids	Alkaloids	496-65-1
MWSm ce341	2.79 E+02	1.49 E+02	2.78E+02	C16H22 O4	[M+H]+	Butyl isobutyl phthalate*	Phenolic acids	Phenolic acids	17851-53-5
Zbzp011 453	2.79 E+02	1.49 E+02	2.78E+02	C16H22 O4	[M+H]+	Des-O-Methyllasiodiplodin	Others	Lactones	32885-82-8
Wbmp0 11762	2.79 E+02	1.49 E+02	2.78E+02	C16H22 O4	[M+H]+	Dibutylphthalate*	Phenolic acids	Phenolic acids	-

Lmxp01 1770	2.79 E+02	1.49 E+02	2.78E+02	C16H22 O4	[M+H]+	Diisobutyl phthalate*	Phenolic acids	Phenolic acids	84-69-5
Ljmp12 1501	2.79 E+02	1.49 E+02	2.78E+02	C16H22 O4	[M+H]+	Mansonone N	Terpenoids	Sesquiterpenoids	-
Hmbp00 0848	2.79 E+02	1.49 E+02	2.78E+02	C16H22 O4	[M+H]+	Phthalic acid Mono-2-ethylhexyl Ester	Phenolic acids	Phenolic acids	4376-20-9
pmp001 230	2.79 E+02	1.21 E+02	2.78E+02	C16H22 O4	[M+H]+	Senkyunolide M	Others	Lactones	146986-60-9
Lmjn01 0508	2.77 E+02	2.05 E+02	2.78E+02	C17H26 O3	[M-H]-	Fusarester B	Others	Ketone compounds	-
Wcsn01 0254	2.77 E+02	2.77 E+02	2.78E+02	C18H30 O2	[M-H]-	4-[3-(4,8-dimethylnona-3,7-dienyl)-3-methyloxiran-2-yl]butan-2-one	Others	Others	-
Wcsn01 0496	2.77 E+02	2.33 E+02	2.78E+02	C18H30 O2	[M-H]-	6,10,14-Trimethylpentadeca-5,9-Diene-2,13-Dione	Others	Others	-
Wcsn01 0375	2.77 E+02	5.90 E+01	2.78E+02	C18H30 O2	[M-H]-	Methyl (4E,8E,11E)-5,9,13-trimethyltetradeca-4,8,11-trienoate	Others	Others	-
Wcdp01 0432	2.80 E+02	2.45 E+02	2.79E+02	C18H33 NO	[M+H]+	Linoleamide	Others	Others	3999-01-7
Zahp012 577	2.80 E+02	8.11 E+01	2.79E+02	C18H33 NO	[M+H]+	Octadecadienamide	Alkaloids	Alkaloids	-

Lmhn003223	2.79E+02	1.47E+02	2.80E+02	C13H12O7	[M-H]-	Cinnamoyltartaric acid	Phenolic acids	Phenolic acids	-
Zmsn110105	2.79E+02	5.90E+01	2.80E+02	C10H16O9	[M-H]-	Rumexobtusifolius	Others	Others	-
Zbsp004026	2.81E+02	2.63E+02	2.80E+02	C14H16O6	[M+H]+	Pteleifolosin A	Others	Others	-
Lmbp010382	2.81E+02	9.11E+01	2.80E+02	C18H16O3	[M+H]+	6-Methoxy-2-(2-phenylethyl)chromone	Others	Chromone	84294-89-3
Wcgn007904	2.79E+02	1.53E+02	2.80E+02	C15H20O5	[M-H]-	3-hydroxy-6-(2-hydroxyethyl)-2,7-bis(hydroxymethyl)-2,5-dimethyl-3H-inden-1-one	Others	Others	-
Zmgp004297	2.81E+02	1.07E+02	2.80E+02	C19H20O2	[M+H]+	1-phenyl-7-(4-hydroxyphenyl)-4-ene-3-heptanone	Others	Ketone compounds	-
Wcsn011122	2.79E+02	2.79E+02	2.80E+02	C18H32O2	[M-H]-	(5Z,10E,14S)-14-hydroxy-2,6,10-trimethylpentadeca-5,10-dien-4-one	Others	Others	-
Wcsn011332	2.79E+02	2.61E+02	2.80E+02	C18H32O2	[M-H]-	Ethyl (10Z,13Z)-hexadeca-10,13-dienoate	Others	Others	-
MWSmce495	2.81E+02	1.24E+02	2.82E+02	C15H22O5	[M-H]-	Octyl gallate	Phenolic acids	Phenolic acids	1034-01-1
Ymmg000006	2.81E+02	1.23E+02	2.82E+02	C15H22O5	[M-H]-	Rugosic acid A	Terpenoids	Sesquiterpenoids	-

Wchp006446	2.85E+02	2.39E+02	2.84E+02	C15H8O6	[M+H]+	1,5-Dihydroxy-9,10-dioxo-9,10-dihydroanthracene-3-carboxylic acid	Others	Others	-
Lmhn002051	2.83E+02	1.67E+02	2.84E+02	C12H12O8	[M-H]-	Vnilloylmalic acid	Phenolic acids	Phenolic acids	-
MWSN0014	2.83E+02	2.68E+02	2.84E+02	C16H12O5	[M-H]-	Acacetin*	Flavonoids	Flavones	480-44-4
mws0908	2.83E+02	2.11E+02	2.84E+02	C16H12O5	[M-H]-	Glycitein	Flavonoids	Isoflavones	40957-83-3
mws0918	2.83E+02	2.68E+02	2.84E+02	C16H12O5	[M-H]-	Prunetin (5,4'-Dihydroxy-7-methoxyisoflavone)*	Flavonoids	Isoflavones	552-59-0
Lmap001821	2.85E+02	1.23E+02	2.84E+02	C13H16O7	[M+H]+	Helicide	Phenolic acids	Phenolic acids	80154-34-3
MWSmce677	2.83E+02	2.65E+02	2.84E+02	C15H24O5	[M-H]-	Dihydroartemisinin	Terpenoids	Sesquiterpenoids	71939-50-9
pmp001269	2.86E+02	2.68E+02	2.85E+02	C18H39NO	[M+H]+	Hexadecyl ethanolamine	Alkaloids	Alkaloids	-
ZBN0041	2.85E+02	1.53E+02	2.86E+02	C15H10O6	[M-H]-	2'-Hydroxygenistein	Flavonoids	Isoflavones	1156-78-1
pme0088	2.85E+02	1.51E+02	2.86E+02	C15H10O6	[M-H]-	Luteolin (5,7,3',4'-Tetrahydroxyflavone)	Flavonoids	Flavones	491-70-3

Wafn002081	2.85E+02	1.53E+02	2.86E+02	C12H14O8	[M-H]-	Dihydroxybenzoyl xyloside	Phenolic acids	Phenolic acids	-
HJN005	2.85E+02	1.39E+02	2.86E+02	C16H14O5	[M-H]-	5,2'-Dihydroxy-7-methoxyflavanone	Flavonoids	Flavones	-
MWSslk035	2.85E+02	1.23E+02	2.86E+02	C13H18O7	[M-H]-	Orcinol glucoside	Phenolic acids	Phenolic acids	21082-33-7
MWSmce194	2.88E+02	2.13E+02	2.87E+02	C17H21NO3	[M+H]+	Galanthamine; Galantamine; Lycoremin; Lycoremine; Jilkon	Alkaloids	Alkaloids	357-70-0
Lmdp003110	2.89E+02	2.43E+02	2.88E+02	C15H12O6	[M+H]+	2,6,7,4'-Tetrahydroxyisoflavanone	Flavonoids	Dihydroisoflavones	-
Zbln008942	2.87E+02	1.21E+02	2.88E+02	C17H20O4	[M-H]-	Batatasin V (2'-Hydroxy-3,4,5-trimethoxybibenzyl)	Others	Stilbene	65817-45-0
MWS0434	2.87E+02	2.69E+02	2.88E+02	C19H28O2	[M-H]-	Trans-dehydrorosinone	Steroids	Steroid	53-43-0
Wcdp006604	2.91E+02	2.45E+02	2.90E+02	C18H10O4	[M+H]+	2-(4'-hydroxyphenyl)naphthalene-1,8-dicarboxylic anhydride	Quinones	Quinones	-
MWSHY0203	2.91E+02	1.23E+02	2.90E+02	C15H14O6	[M+H]+	Catechin	Flavonoids	Flavanols	154-23-4
Wafp002664	2.93E+02	1.47E+02	2.92E+02	C15H20N2O4	[M+H]+	1-O-p-Coumaroyllysine	Alkaloids	Phenolamine	-

pmb2893	2.91E+02	1.19E+02	2.92E+02	C14H20N4O3	[M-H]-	N-p-Coumaroylhydroxyagmatine	Alkaloids	Phenolamine	-
Zbzp007301	2.93E+02	5.51E+01	2.92E+02	C18H28O3	[M+H]+	2-Methoxy-6-undecyl-1,4-benzoquinone	Quinones	Quinones	-
Qmyp101336	2.93E+02	1.49E+02	2.92E+02	C18H28O3	[M+H]+	JiangxiBaiyingsu I	Terpenoids	Sesquiterpenoids	-
MWSmce098	2.94E+02	1.37E+02	2.93E+02	C17H27NO3	[M+H]+	Nonivamide*	Alkaloids	Phenolamine	2444-46-4
mws2216	2.94E+02	1.37E+02	2.93E+02	C17H27NO3	[M+H]+	Nordihydrocapsaicin*	Alkaloids	Phenolamine	28789-35-7
Zbzn008048	2.93E+02	2.21E+02	2.94E+02	C17H26O4	[M-H]-	Embelin	Quinones	Quinones	550-24-3
Zjxp110301	2.95E+02	1.51E+02	2.94E+02	C18H30O3	[M+H]+	3-Hydroxy-3,7,11-trimethyldodeca-1,6E,10-trien-9-yl isobutyrate	Terpenoids	Sesquiterpenoids	-
Zjjp092610	2.95E+02	1.79E+02	2.94E+02	C18H30O3	[M+H]+	Dendronobilin I	Terpenoids	Sesquiterpenoids	-
MWSmce223	2.96E+02	2.50E+02	2.95E+02	C19H21NO2	[M+H]+	Nuciferine	Alkaloids	Aporphine alkaloids	475-83-2
zjbp110823	2.97E+02	1.21E+02	2.96E+02	C18H16O4	[M+H]+	6-hydroxy-2-[2-(4-methoxyphenyl)ethyl]chromone	Others	Chromone	125092-36-6

Hmmp001541	2.98E+02	1.36E+02	2.97E+02	C17H15NO4	[M+H]+	3-(3',5'-Dimethoxy-4'-hydroxy-benzylidene')-2-indolinone	Alkaloids	Plumerane	-
Hmhp011220	2.98E+02	1.61E+02	2.97E+02	C16H27NO4	[M+H]+	10,11-Dihydroxy-N-(2-hydroxy-2-methylpropyl)-2,6,8-dodecatrienam-Ide	Alkaloids	Alkaloids	-
Lmzp009151	2.98E+02	1.41E+02	2.97E+02	C18H35NO2	[M+H]+	Lepadin D	Alkaloids	Quinoline alkaloids	-
Wbkp007750	2.99E+02	2.53E+02	2.98E+02	C16H14N2O4	[M+H]+	1-hydroxy-4-methoxy-3-methyl-2,3-dihydro-1H-indolo[3,2,1-de][1,5]naphthyridine-5,6-dione	Alkaloids	Alkaloids	-
ZbWn003804	2.97E+02	9.70E+01	2.98E+02	C12H26O6S	[M-H]-	8-(methoxymethoxy)decyl hydrogen sulfate	Others	Others	-
Lmhn001386	2.99E+02	1.67E+02	3.00E+02	C12H12O9	[M-H]-	Vnilloyltartaric acid	Phenolic acids	Phenolic acids	-
Lmjp004941	3.01E+02	2.86E+02	3.00E+02	C16H12O6	[M+H]+	3,5,4'-Trihydroxy-7-methoxyflavone (Rhamnocitrin)*	Flavonoids	Flavonols	569-92-6
MWSHC20114	3.01E+02	2.86E+02	3.00E+02	C16H12O6	[M+H]+	5,7,2'-Trihydroxy-8-methoxyflavone; Scutevulin*	Flavonoids	Flavones	80713-32-2
Xmyn008071	2.99E+02	2.84E+02	3.00E+02	C16H12O6	[M-H]-	5,7-dihydroxy-2-(3-hydroxyphenyl)-3-methoxy-4H-chromen-4-one*	Flavonoids	Flavonols	140671-06-3
Zmhp003514	3.01E+02	2.86E+02	3.00E+02	C16H12O6	[M+H]+	6,7,8-Tetrahydroxy-5-methoxyflavone	Flavonoids	Flavones	-

MWSHC20110	3.01E+02	2.86E+02	3.00E+02	C16H12O6	[M+H]+	8-Methoxyapigenin*	Flavonoids	Flavones	57096-02-3
Wbtn007603	2.99E+02	2.84E+02	3.00E+02	C16H12O6	[M-H]-	9,11-dimethoxy-2h-[1,3]dioxolo[4,5-b]xanthen-10-one*	Flavonoids	Other Flavonoids	-
Lmhn004976	2.99E+02	2.84E+02	3.00E+02	C16H12O6	[M-H]-	Aracarpene 2*	Flavonoids	Isoflavones	-
mws0058	2.99E+02	2.84E+02	3.00E+02	C16H12O6	[M-H]-	Diosmetin (5,7,3'-Trihydroxy-4'-methoxyflavone)*	Flavonoids	Flavones	520-34-3
MWSHY0069	3.01E+02	2.86E+02	3.00E+02	C16H12O6	[M+H]+	Hispidulin (5,7,4'-Trihydroxy-6-methoxyflavone)*	Flavonoids	Flavones	1447-88-7
pmc1990	2.99E+02	1.19E+02	3.00E+02	C17H16O5	[M-H]-	4'-Hydroxy-5,7-dimethoxyflavanone	Flavonoids	Flavanones	-
Lhmp122205	3.01E+02	2.45E+02	3.00E+02	C18H20O4	[M+H]+	5-O-Methyllatifolin*	Others	Others	-
Wdbp008550	3.01E+02	2.45E+02	3.00E+02	C18H20O4	[M+H]+	Stemanthrene C*	Others	Others	-
Ylgn010606	2.99E+02	2.99E+02	3.00E+02	C20H28O2	[M-H]-	triptonoterpene	Terpenoids	Ditepenoids	-
Wcfp007836	3.02E+02	8.81E+01	3.01E+02	C18H39NO2	[M+H]+	Tetradecyldiethanolamine	Alkaloids	Alkaloids	18924-66-8

pme2246	3.01E+02	1.85E+02	3.02E+02	C14H6O8	[M-H]-	Ellagic acid	Tannins	Tannin	476-66-4
Zmpp002571	3.03E+02	2.57E+02	3.02E+02	C15H10O7	[M+H]+	3,3',4',5',7-Pentahydroxyflavone; Robinetin	Flavonoids	Flavonols	490-31-3
Cmsp004086	3.03E+02	2.29E+02	3.02E+02	C16H14O6	[M+H]+	Dihydrokaempferide	Flavonoids	Flavanonols	137225-59-3
Zaxn006988	3.01E+02	3.01E+02	3.02E+02	C20H30O2	[M-H]-	Isopimaric acid*	Terpenoids	Ditepenoids	5835-26-7
MWSslk208	3.01E+02	3.01E+02	3.02E+02	C20H30O2	[M-H]-	Kaurenoic Acid*	Terpenoids	Ditepenoids	6730-83-2
Cmmn013378	3.01E+02	3.01E+02	3.02E+02	C20H30O2	[M-H]-	Levopimaric acid	Terpenoids	Ditepenoids	79-54-9
mws1375	3.04E+02	1.85E+02	3.03E+02	C12H21N3O6	[M+H]+	Nicotianamine	Alkaloids	Alkaloids	34441-14-0
Wasn007177	3.03E+02	1.83E+02	3.04E+02	C15H12O7	[M-H]-	3-O-Hydroxybenzoyl Methylgallate	Phenolic acids	Phenolic acids	-
Lmfn001893	3.05E+02	1.25E+02	3.06E+02	C15H14O7	[M-H]-	Leucocyanidin	Flavonoids	Flavanols	27015-21-0
pmb0496	3.07E+02	1.77E+02	3.06E+02	C15H22N4O3	[M+H]+	N-Feruloylagmatine	Alkaloids	Phenolamine	-

pmb2601	3.07E+02	1.61E+02	3.08E+02	C15H16O7	[M-H]-	7-Hydroxycoumarin-O-rhamnoside	Lignans and Coumarins	Coumarins	-
mws1567	3.07E+02	1.19E+02	3.08E+02	C19H16O4	[M-H]-	Bisdemethoxycurcumin	Phenolic acids	Phenolic acids	33171-05-0
ZbWp001588	3.10E+02	1.20E+02	3.09E+02	C15H19NO6	[M+H]+	3-(((S)-1-carboxy-2-phenylethyl)amino)-2,5-dihydroxycyclopentane-1-carboxylic acid	Others	Others	-
Lmhn001477	3.11E+02	1.79E+02	3.12E+02	C13H12O9	[M-H]-	2-Caffeoyl-L-tartaric acid (Caftaric acid)	Phenolic acids	Phenolic acids	67879-58-7
Qmyp101415	3.13E+02	1.35E+02	3.12E+02	C18H32O4	[M+H]+	Septemlobin D	Terpenoids	Sesquiterpenoids	-
Lmgn009690	3.13E+02	2.55E+02	3.14E+02	C17H14O6	[M-H]-	3,5-Dihydroxy-7,4'-dimethoxyflavone	Flavonoids	Flavonols	15486-33-6
mws1174	3.13E+02	2.53E+02	3.14E+02	C17H14O6	[M-H]-	3-O-Acetylpinobanksin	Flavonoids	Flavanonols	52117-69-8
mws0038	3.13E+02	2.83E+02	3.14E+02	C17H14O6	[M-H]-	5,4'-Dihydroxy-3,7-dimethoxyflavone(Kumatakenin)	Flavonoids	Flavonols	3301-49-3
Wbtp007858	3.15E+02	2.82E+02	3.14E+02	C17H14O6	[M+H]+	7-O-Methyltectorigenin; 4',5-Dihydroxy-6,7-Dimethoxyisoflavone*	Flavonoids	Isoflavones	1096-58-8
HJAP057	3.15E+02	2.82E+02	3.14E+02	C17H14O6	[M+H]+	Dihydroxy-dimethoxyflavone*	Flavonoids	Flavonols	-

Ymjm000116	3.13E+02	1.51E+02	3.14E+02	C14H18O8	[M-H]-	6-O-Acetylarbutin	Others	Others	10338-88-2
MWSHY0120	3.15E+02	1.81E+02	3.14E+02	C18H18O5	[M+H]+	Flavokawain A	Flavonoids	Chalcones	3420-72-2
Lhbp073003	3.14E+02	2.69E+02	3.14E+02	C19H24NO3+	[M]+	Magnocurarine	Alkaloids	Isoquinoline alkaloids	6801-40-7
Lcmp122922	3.15E+02	2.73E+02	3.14E+02	C21H30O2	[M+H]+	Sugiol methyl ether	Terpenoids	Ditepenoids	-
Zbzp002197	3.16E+02	1.72E+02	3.15E+02	C15H25NO6	[M+H]+	lycopsamine N-oxide	Alkaloids	Pyrrole alkaloids	95462-15-0
MWS1116	3.16E+02	8.50E+01	3.15E+02	C17H33NO4	[M+H]+	Decanoyl L-Carnitine	Alkaloids	Alkaloids	3992-45-8
Lmqn004838	3.15E+02	3.00E+02	3.16E+02	C15H8O8	[M-H]-	3-O-Methylellagic acid	Tannins	Tannin	-
Lmfp001509	3.17E+02	1.53E+02	3.16E+02	C13H16O9	[M+H]+	1-O-Galloyl-rhamnose	Phenolic acids	Phenolic acids	-
ZBN0311	3.15E+02	1.69E+02	3.16E+02	C13H16O9	[M-H]-	Maplexin B (4-O-Galloyl-1,5-Anhydro-D-Glucitol)	Tannins	Tannin	-
Wcgn005541	3.15E+02	2.71E+02	3.16E+02	C17H16O6	[M-H]-	Cyrtominetin	Flavonoids	Flavones	95272-99-4

pmn001411	3.15E+02	1.35E+02	3.16E+02	C14H20O8	[M-H]-	Cornoside	Phenolic acids	Phenolic acids	40661-45-8
pme3459	3.17E+02	2.99E+02	3.16E+02	C20H28O3	[M+H]+	Cafestol	Terpenoids	Ditepenoids	469-83-0
Cmqn009444	3.15E+02	2.57E+02	3.16E+02	C21H32O2	[M-H]-	3-(11,13-Pentadecadienyl)-(Z,E)1,2-Benzenediol*	Phenolic acids	Phenolic acids	155475-58-4
Cmqn009387	3.15E+02	2.57E+02	3.16E+02	C21H32O2	[M-H]-	3-(7,10-Pentadecadienyl)-1,2-benzenediol*	Phenolic acids	Phenolic acids	68640-60-8
ZBN0174	3.17E+02	1.51E+02	3.18E+02	C15H10O8	[M-H]-	Myricetin	Flavonoids	Flavonols	529-44-2
Hmgp001996	3.19E+02	2.73E+02	3.18E+02	C15H10O8	[M+H]+	Quercetagetin; 3,3',4',5,6,7-Hexahydroxyflavone	Flavonoids	Flavonols	90-18-6
Yshj000013	3.19E+02	1.25E+02	3.18E+02	C13H18O9	[M+H]+	4,6-dimethoxy-2-methoxyphenyl-1-O-beta-D-glucopyranoside	Others	Others	-
Wbjp002336	3.19E+02	3.01E+02	3.18E+02	C16H18N2O5	[M+H]+	N,N-diethyl-5-hydroxytryptamineoxalate	Alkaloids	Alkaloids	-
pmp000970	3.19E+02	3.01E+02	3.18E+02	C20H30O3	[M+H]+	Hispanolone	Terpenoids	Ditepenoids	18676-07-8
Lssp210094	3.20E+02	2.01E+02	3.19E+02	C12H21N3O7	[M+H]+	2''-hydroxynicotinamide	Alkaloids	Alkaloids	-

Hmsp007613	3.21E+02	1.37E+02	3.20E+02	C19H28O4	[M+H]+	[8]-Gingerdione	Phenolic acids	Phenolic acids	77334-06-6
Ymjm000067	3.21E+02	1.79E+02	3.20E+02	C20H32O3	[M+H]+	14-Hydroxyvibsanin F	Terpenoids	Ditepenoids	-
Lmsp013116	3.21E+02	1.23E+02	3.20E+02	C20H32O3	[M+H]+	Ent-16α,17-Dihydroxykauran-2-one	Terpenoids	Ditepenoids	-
Qmxn081902	3.19E+02	2.75E+02	3.20E+02	C20H32O3	[M-H]-	Minheryin D	Terpenoids	Ditepenoids	-
Ladp008157	3.21E+02	1.09E+02	3.20E+02	C20H32O3	[M+H]+	Pilosanone A	Terpenoids	Monoterpenoids	-
Wmln004117	3.20E+02	1.31E+02	3.21E+02	C16H19NO6	[M-H]-	4-[(3-O-Acetyl-6-deoxy-α-L-mannosyl)oxy]benzeneacetonitrile	Alkaloids	Phenolamine	159397-73-6
YC512112	3.25E+02	1.63E+02	3.24E+02	C16H24O5N2	[M+H]+	Anabasine-glucoside	Alkaloids	Pyridine alkaloids	-
Zbqp000552	3.25E+02	1.63E+02	3.25E+02	C16H25N2O5+	[M]+	Nicotine N-glucoside	Alkaloids	Pyridine alkaloids	-
mws0983	3.26E+02	6.21E+01	3.25E+02	C20H39NO2	[M+H]+	N-Oleoylethanolamine	Alkaloids	Alkaloids	111-58-0
pmn001515	3.25E+02	9.30E+01	3.26E+02	C14H14O9	[M-H]-	3-Galloylshikimic acid	Phenolic acids	Phenolic acids	110082-91-2

Zmbp001962	3.27E+02	2.01E+02	3.26E+02	C18H18N2O4	[M+H]+	Isaindigodione	Alkaloids	Plumerane	-
Lamp009618	3.29E+02	2.83E+02	3.28E+02	C17H12O7	[M+H]+	2-Hydroxy-scillascillin	Flavonoids	Other Flavonoids	-
Lmhn003897	3.27E+02	2.95E+02	3.28E+02	C17H12O7	[M-H]-	Aflatoxin M4	Others	Lactones	104700-21-2
Lmsp008264	3.29E+02	2.68E+02	3.28E+02	C18H16O6	[M+H]+	4'-Hydroxy-5,6,7-trimethoxyflavone	Flavonoids	Flavones	6938-18-7
Hmxp006586	3.29E+02	2.99E+02	3.28E+02	C18H16O6	[M+H]+	5-Hydroxy-7,3',4'-trimethoxyflavone*	Flavonoids	Flavones	29080-58-8
Lmsp011263	3.29E+02	2.68E+02	3.28E+02	C18H16O6	[M+H]+	6-Hydroxy-5,7,4'-trimethoxyflavone	Flavonoids	Flavones	6938-19-8
Wagp007355	3.29E+02	2.99E+02	3.28E+02	C18H16O6	[M+H]+	8-Hydroxy-4',5,7-trimethoxyflavone*	Flavonoids	Flavones	21919-71-1
Labn003679	3.27E+02	1.47E+02	3.28E+02	C15H20O8	[M-H]-	Caffeyl alcohol 4-O-β-D-glucopyranoside	Others	Alcohol compounds	-
Hmsn002272	3.27E+02	1.65E+02	3.28E+02	C15H20O8	[M-H]-	Demethyl coniferin	Others	Alcohol compounds	-
Lmqp006559	3.30E+02	3.12E+02	3.29E+02	C20H43NO2	[M+H]+	2-Amino-1,3-eicosanediol	Others	Alcohol compounds	-

pme2768	3.29E+02	2.41E+02	3.30E+02	C14H6N2O8	[M-H]-	Pyrroloquinoline quinone	Alkaloids	Quinoline alkaloids	72909-34-3
Zbln006102	3.29E+02	2.99E+02	3.30E+02	C16H10O8	[M-H]-	3,3'-O-Dimethylellagic Acid	Tannins	Tannin	2239-88-5
ZBN0060	3.29E+02	3.14E+02	3.30E+02	C17H14O7	[M-H]-	Quercetin-3,4'-Dimethyl Ether	Flavonoids	Flavonols	33429-83-3
Lmzp004885	3.31E+02	3.15E+02	3.30E+02	C17H14O7	[M+H]+	Tricin (5,7,4'-Trihydroxy-3',5'-dimethoxyflavone)	Flavonoids	Flavones	520-32-1
Jmyn006479	3.29E+02	1.39E+02	3.30E+02	C18H18O6	[M-H]-	3,5,7-Trihydroxy-6,8-dimethyl-3-(4'-hydroxybenzyl)-chroman-4-one (Polygonatone C)	Flavonoids	Other Flavonoids	-
Sadn014276	3.29E+02	3.01E+02	3.30E+02	C21H30O3	[M-H]-	2-Geranyl-5-Hydroxy-3-N-Pentyl-1,4-Benzoquinone	Terpenoids	Sesquiterpenoids	-
Lmmn004951	3.29E+02	1.71E+02	3.30E+02	C18H34O5	[M-H]-	2-Hydroxy-4-methyl-3-undecanoyloxypentanoic acid methyl ester	Others	Others	-
Qmgp102504	3.32E+02	1.52E+02	3.31E+02	C20H17N3O2	[M+H]+	Angustoline	Alkaloids	Plumerane	40041-95-0
Hmln000659	3.31E+02	1.69E+02	3.32E+02	C13H16O10	[M-H]-	3-O-Galloyl-D-glucose*	Phenolic acids	Phenolic acids	-
Zmhn001703	3.31E+02	1.69E+02	3.32E+02	C13H16O10	[M-H]-	5-O-Galloyl-D-hamamelose*	Phenolic acids	Phenolic acids	-

Lmfn000604	3.31E+02	1.69E+02	3.32E+02	C13H16O10	[M-H]-	6-O-Galloyl-β-D-glucose*	Phenolic acids	Phenolic acids	13186-19-1
Zmxn110201	3.31E+02	1.08E+02	3.32E+02	C14H20O9	[M-H]-	2,6-Dimethoxy-4-hydroxyphenol-1-O-ß-D-glucopyranoside	Others	Others	-
Lmmn003088	3.31E+02	1.27E+02	3.32E+02	C14H20O9	[M-H]-	2,6-Dimethoxyhydroquinone-1-O-glucoside	Others	Others	-
Jmwn002117	3.31E+02	1.53E+02	3.32E+02	C14H20O9	[M-H]-	2,6-dimethoxybenzene-1,4-diol 1-O-β-D-glucopyranoside	Phenolic acids	Phenolic acids	-
Lmmn001294	3.31E+02	1.53E+02	3.32E+02	C14H20O9	[M-H]-	Koaburaside	Others	Others	41653-73-0
Cmrp004395	3.33E+02	2.87E+02	3.32E+02	C16H28O7	[M+H]+	2,6-Dimethyl-6-hydroxy-2,7-octadienyl-β-D-glucoside (Betulalbuside A; Betulabuside A)	Terpenoids	Monoterpenoids	64776-96-1
Wmcp000015	3.33E+02	3.15E+02	3.32E+02	C20H28O4	[M+H]+	clerodermic acid	Terpenoids	Monoterpenoids	-
Lamp011743	3.33E+02	1.21E+02	3.32E+02	C21H32O3	[M+H]+	2,3-Dihydroxy-5α-pregn-17(Z)-en-16-one	Steroids	Steroid	-
Hmqn005002	3.31E+02	2.99E+02	3.32E+02	C21H32O3	[M-H]-	12-Hydroxymethylabietate	Terpenoids	Ditepenoids	-
Lmfp012576	3.33E+02	1.25E+02	3.32E+02	C21H32O3	[M+H]+	Fusosteride A	Steroids	Steroid	-

MWSmce268	3.33E+02	3.15E+02	3.34E+02	C20H30O4	[M-H]-	Lathyrol	Terpenoids	Ditepenoids	34420-19-4
Wbhp009564	3.35E+02	3.35E+02	3.34E+02	C21H34O3	[M+H]+	Methyl 8,9-epoxypimaran-18-oate	Terpenoids	Terpene	42855-38-9
Zmhn005413	3.35E+02	1.83E+02	3.36E+02	C15H12O9	[M-H]-	p-Dimeric galloyl methyl ester	Phenolic acids	Phenolic acids	-
Zbsp010536	3.37E+02	1.37E+02	3.36E+02	C20H32O4	[M+H]+	Methyl[8]-gingerol	Phenolic acids	Phenolic acids	-
Lmqn003856	3.37E+02	1.75E+02	3.38E+02	C16H18O8	[M-H]-	1,4,8-Trihydroxynaphthalene-1-O-glucoside	Quinones	Naphthol	39015-63-9
MWSN0037	3.37E+02	1.19E+02	3.38E+02	C21H22O4	[M-H]-	4'-O-Methylbavachalcone	Flavonoids	Chalcones	20784-60-5
Lmmn009722	3.37E+02	2.35E+02	3.38E+02	C21H22O4	[M-H]-	Cajaninstilbene Acid	Phenolic acids	Phenolic acids	87402-84-4
Lmmn006246	3.37E+02	1.57E+02	3.38E+02	C20H34O4	[M-H]-	7β-Hydroxydarutigenol	Terpenoids	Ditepenoids	1188281-99-3
Lcsn000655	3.37E+02	2.91E+02	3.38E+02	C20H34O4	[M-H]-	ent-3β,16α,17,19-Tetrahydroxykaurane	Terpenoids	Ditepenoids	-
Lmbn001162	3.39E+02	1.77E+02	3.40E+02	C15H16O9	[M-H]-	Esculetin-7-O-glucoside	Lignans and Coumarins	Coumarins	-

Wmkn004416	3.39E+02	1.93E+02	3.40E+02	C20H20O5	[M-H]-	Desmethylxanthohumol	Flavonoids	Chalcones	115063-39-3
Zapn009595	3.39E+02	2.19E+02	3.40E+02	C20H20O5	[M-H]-	Sophoraflavanone B	Flavonoids	Flavanones	53846-50-7
pmp000541	3.42E+02	2.97E+02	3.41E+02	C20H23NO4	[M+H]+	Corydine	Alkaloids	Aporphine alkaloids	476-69-7
pmn001420	3.41E+02	1.79E+02	3.42E+02	C15H18O9	[M-H]-	1-O-Caffeoyl-β-D-glucose*	Phenolic acids	Phenolic acids	14364-08-0
Zmhn001793	3.41E+02	1.79E+02	3.42E+02	C15H18O9	[M-H]-	6-O-Caffeoyl-D-glucose*	Phenolic acids	Phenolic acids	-
Wagp009375	3.43E+02	3.13E+02	3.42E+02	C19H18O6	[M+H]+	3,5,6,7-Tetramethoxyflavone*	Flavonoids	Flavonols	75413-07-9
MWSHC2050	3.43E+02	3.13E+02	3.42E+02	C19H18O6	[M+H]+	5,6,7,4'-Tetramethoxyflavone*	Flavonoids	Flavones	1168-42-9
Hamp007846	3.43E+02	3.13E+02	3.42E+02	C19H18O6	[M+H]+	5,6,7,8-Tetramethoxyflavone	Flavonoids	Flavones	-
MWSHY0128	3.43E+02	3.13E+02	3.42E+02	C19H18O6	[M+H]+	5,7,8,4'-Tetramethoxyflavone	Flavonoids	Flavones	6601-66-7
Wdhp004132	3.44E+02	1.92E+02	3.43E+02	C20H25NO4	[M+H]+	1-[(3,4-dimethoxyphenyl)methyl]-7-methoxy-2-methyl-3,4-dihydro-1h-isoquinolin-6-ol	Alkaloids	Isoquinoline alkaloids	-

Lmyn00 0503	3.43 E+02	8.50 E+01	3.44E+02	C13H12 O11	[M-H]-	Mucic acid-1,4-lactone-2-O-gallate	Phenolic acids	Phenolic acids	-
ZBN015 9	3.43 E+02	3.13 E+02	3.44E+02	C17H12 O8	[M-H]-	3,3',4-O-Trimethylellagic acid	Tannins	Tannin	1617-49-8
Zbyn00 1562	3.43 E+02	1.91 E+02	3.44E+02	C14H16 O10	[M-H]-	3-Galloylquinic acid	Phenolic acids	Phenolic acids	17365-11-6
mws147 4	3.45 E+02	2.84 E+02	3.44E+02	C18H16 O7	[M+H]+	5,7-Dihydroxy-3',4',5'-trimethoxyflavone	Flavonoids	Flavones	18103-42-9
Zbsp005 869	3.45 E+02	3.15 E+02	3.44E+02	C18H16 O7	[M+H]+	7,4'-Dihydroxy-3,5,3'-trimethoxyflavone	Flavonoids	Flavones	-
Zmhp00 4065	3.45 E+02	3.30 E+02	3.44E+02	C18H16 O7	[M+H]+	7,8-Dihydroxy-5,6,4'-trimethoxyflavone*	Flavonoids	Flavones	-
Lmjn00 5592	3.43 E+02	3.13 E+02	3.44E+02	C18H16 O7	[M-H]-	Cirsilineol (4',5-Dihydroxy-3',6,7-trimethoxyflavone)	Flavonoids	Flavanones	41365-32-6
pmp000 006	3.45 E+02	3.30 E+02	3.44E+02	C18H16 O7	[M+H]+	Eupatilin (5,7-Dihydroxy-3',4',6-Trimethoxyflavone)*	Flavonoids	Flavones	22368-21-4
MWSH Y0054	3.45 E+02	3.12 E+02	3.44E+02	C18H16 O7	[M+H]+	Eupatorin; 3',5-Dihydroxy-4',6,7-Trimethoxyflavone	Flavonoids	Flavones	855-96-9
MWSslk 092	3.43 E+02	3.28 E+02	3.44E+02	C18H16 O7	[M-H]-	Usnic acid	Others	Ketone compounds	125-46-2

Lmqn00 0859	3.43 E+02	1.71 E+02	3.44E+02	C15H20 O9	[M-H]-	4-O-Glucosyl-2-hydroxy-6-methoxyacetophenone	Phenolic acids	Phenolic acids	-
Zbfn002 396	3.43 E+02	1.63 E+02	3.44E+02	C15H20 O9	[M-H]-	6-O-Dihydrocaffeoylglucose	Phenolic acids	Phenolic acids	-
Zmhn00 0563	3.43 E+02	1.97 E+02	3.44E+02	C15H20 O9	[M-H]-	Syringic acid 4-O-rhamnoside	Phenolic acids	Phenolic acids	-
Wagp00 9755	3.45 E+02	2.11 E+02	3.44E+02	C19H20 O6	[M+H]+	6'-Hydroxy-4,2',3',4'-Tetramethoxychalcone	Flavonoids	Chalcones	3877-67-6
Hmgn00 2254	3.45 E+02	2.85 E+02	3.46E+02	C17H14 O8	[M-H]-	5,7,3'-Trihydroxy-4,6,5'-trimethoxyxanthone	Flavonoids	Other Flavonoids	-
Lmmn0 04792	3.45 E+02	2.63 E+02	3.46E+02	C19H22 O6	[M-H]-	9α-Hydroxy-8β-methacryloyloxy-14-oxo-acanthospermolide	Terpenoids	Sesquiterpenoids	-
Smmp0 08919	3.47 E+02	3.01 E+02	3.46E+02	C20H26 O5	[M+H]+	Epirosmanol	Terpenoids	Ditepenoids	-
Lcsp003 365	3.52 E+02	2.20 E+02	3.51E+02	C15H21 N5O5	[M+H]+	cis-Zeatin riboside	Alkaloids	Alkaloids	15896-46-5
MWSprf 097	3.52 E+02	1.20 E+02	3.51E+02	C18H25 NO6	[M+H]+	USaramine	Alkaloids	Alkaloids	15503-87-4
Wbhp00 8068	3.53 E+02	3.53 E+02	3.52E+02	C20H32 O5	[M+H]+	(1R,4aS,4bR,7R,8R,8aR,9S,10aR)-7-ethenyl-8,8a,9-trihydroxy-1,4a,7-trimethyl-2,3,4,4b,5,6,8,9,10,10a-decahydrophenanthrene-1-carboxylic acid	Terpenoids	Terpene	-

Wcgn009980	3.51E+02	2.69E+02	3.52E+02	C20H32O5	[M-H]-	7-(1,2-Dihydroxyethyl)-2-hydroxy-1-(hydroxymethyl)-1,4b,7-trimethyl-2,5,6,8,8a,9,10,10a-octahydrophenanthren-3-one	Terpenoids	Ditepenoids	-
pme1816	3.53E+02	1.91E+02	3.54E+02	C16H18O9	[M-H]-	Neochlorogenic acid (5-O-Caffeoylquinic acid)	Phenolic acids	Phenolic acids	906-33-2
mws4113	3.53E+02	2.95E+02	3.54E+02	C21H22O5	[M-H]-	3'-Hydroxy-4'-O-methylglabridin	Flavonoids	Other Flavonoids	175554-11-7
MWSN0042	3.53E+02	2.33E+02	3.54E+02	C21H22O5	[M-H]-	Xanthohumol	Flavonoids	Chalcones	6754-58-1
Wabn004002	3.55E+02	2.23E+02	3.56E+02	C16H20O9	[M-H]-	3'-O-Beta-D-xylopyranosyl plumbagic acid	Phenolic acids	Phenolic acids	-
Zmhn003082	3.57E+02	1.73E+02	3.58E+02	C15H18O10	[M-H]-	5-O-Methylgalloylquinic Acid	Phenolic acids	Phenolic acids	-
pmp000008	3.59E+02	3.44E+02	3.58E+02	C19H18O7	[M+H]+	5-Desmethylsinensetin*	Flavonoids	Flavones	21763-80-4
Wagp009942	3.59E+02	3.44E+02	3.58E+02	C19H18O7	[M+H]+	5-Hydroxy-4',6,7,8-tetramethoxyflavone*	Flavonoids	Flavones	1245-15-4
Zbgp008956	3.59E+02	3.26E+02	3.58E+02	C19H18O7	[M+H]+	5-Hydroxy-7,8,2',6'-Tetramethoxyflavone (Altisin)	Flavonoids	Flavones	-
pmp000110	3.59E+02	3.44E+02	3.58E+02	C19H18O7	[M+H]+	7-Hydroxy-3,5,6,8-tetramethoxyflavone	Flavonoids	Flavonols	-

Lmsp010393	3.59E+02	2.98E+02	3.58E+02	C19H18O7	[M+H]+	Eupatorin-5-methylether (3'-hydroxy-5,6,7,4'-tetramethoxyflavone)	Flavonoids	Flavones	21764-09-0
MWSHC20189	3.57E+02	1.51E+02	3.58E+02	C20H22O6	[M-H]-	Epipinoresinol*	Lignans and Coumarins	Lignans	24404-50-0
mws0097	3.57E+02	1.51E+02	3.58E+02	C20H22O6	[M-H]-	Pinoresinol*	Lignans and Coumarins	Lignans	487-36-5
Lmmn006932	3.57E+02	2.97E+02	3.58E+02	C20H38O5	[M-H]-	2-Hydroxy-4-methyl-3-tridecanoyloxypentanoic acid methyl ester	Others	Others	-
Wagp009674	3.61E+02	1.97E+02	3.60E+02	C19H20O7	[M+H]+	3',6'-Dihydroxy-3,4,2',4'-Tetramethoxychalcone	Flavonoids	Chalcones	-
Lmyn000716	3.61E+02	2.09E+02	3.62E+02	C13H14O12	[M-H]-	2-O-Galloylmucic acid	Phenolic acids	Phenolic acids	-
Lmmn002747	3.61E+02	1.80E+02	3.62E+02	C20H26O6	[M-H]-	Secoisolariciresinol	Lignans and Coumarins	Lignans	29388-59-8
Lmbn015605	3.61E+02	3.17E+02	3.62E+02	C22H34O4	[M-H]-	Phthalic acid,6-ethyl-3-octylbutyl ester	Phenolic acids	Phenolic acids	-
Wbtn009057	3.63E+02	3.27E+02	3.64E+02	C17H16O9	[M-H]-	1,6,8-trihydroxy-2,3,4,7-tetramethoxyxanthen-9-one	Flavonoids	Other Flavonoids	-
Wasn002329	3.66E+02	1.60E+02	3.67E+02	C16H17NO9	[M-H]-	Xanthurenic Acid 8-O-Glucoside	Alkaloids	Quinoline alkaloids	97451-32-6

MWSHY0156	3.69E+02	3.13E+02	3.68E+02	C21H20O6	[M+H]+	β-Anhydroicaritin	Flavonoids	Other Flavonoids	38226-86-7
Wacn002584	3.69E+02	1.27E+02	3.70E+02	C15H14O11	[M-H]-	2-O-Caffeoylhydroxycitric Acid	Phenolic acids	Phenolic acids	-
Wafn003070	3.71E+02	2.09E+02	3.72E+02	C16H20O10	[M-H]-	Hydroxyferulic acid glucoside	Phenolic acids	Phenolic acids	-
pmb3317	3.71E+02	1.79E+02	3.72E+02	C16H20O10	[M-H]-	Quinacyl syringic acid	Phenolic acids	Phenolic acids	-
Zajp007311	3.73E+02	3.43E+02	3.72E+02	C20H20O7	[M+H]+	3',4',5',5,7-Pentamethoxyflavone*	Flavonoids	Flavones	53350-26-8
Lmsp010424	3.73E+02	2.11E+02	3.72E+02	C20H20O7	[M+H]+	3,4',5,6,7-Pentamethoxyflavone	Flavonoids	Flavonols	4472-73-5
Zmsp007375	3.73E+02	3.43E+02	3.72E+02	C20H20O7	[M+H]+	3,5,7,3'4'-Pentamethoxyflavone	Flavonoids	Flavonols	1247-97-8
MWSHC2053	3.73E+02	3.12E+02	3.72E+02	C20H20O7	[M+H]+	5-Demethoxynobiletin; 3',4',6,7,8-Pentamethoxyflavone*	Flavonoids	Flavones	42206-91-7
pmp001076	3.73E+02	3.43E+02	3.72E+02	C20H20O7	[M+H]+	Isosinensetin	Flavonoids	Flavones	17290-70-9
mws1313	3.73E+02	3.12E+02	3.72E+02	C20H20O7	[M+H]+	Sinensetin (5,6,7,3',4'-pentamethoxyflavone)*	Flavonoids	Flavones	2306-27-6

mws0055	3.73E+02	3.43E+02	3.72E+02	C20H20O7	[M+H]+	Tangeretin (4',5,6,7,8-Pentamethoxyflavone)*	Flavonoids	Flavones	481-53-8
Wbwp008548	3.73E+02	3.43E+02	3.72E+02	C20H20O7	[M+H]+	[(1R,2S)-1-(1,3-benzodioxol-5-yl)-2-methyl-3-oxobutyl]4-hydroxy-3-methoxybenzoate	Lignans and Coumarins	Lignans	-
Zbzn013709	3.71E+02	2.69E+02	3.72E+02	C21H24O6	[M-H]-	isovalerylshikonin	Quinones	Quinones	52387-14-1
HJN008	3.71E+02	2.07E+02	3.72E+02	C22H28O5	[M-H]-	Ganschisandrin	Lignans and Coumarins	Lignans	122872-00-8
Jmbp005771	3.75E+02	3.45E+02	3.74E+02	C19H18O8	[M+H]+	Gardenin D	Flavonoids	Flavones	29202-00-4
Lmsp009394	3.75E+02	2.11E+02	3.74E+02	C20H22O7	[M+H]+	3',4',5,6,7-Pentamethoxyflavanone*	Flavonoids	Flavanones	-
Hmxp007703	3.75E+02	2.11E+02	3.74E+02	C20H22O7	[M+H]+	5,7,8,3',4'-Pentamethoxyflavanone*	Flavonoids	Flavanones	-
Wagp010141	3.75E+02	2.41E+02	3.74E+02	C20H22O7	[M+H]+	6'-Hydroxy-2,2',3',4',5'-Pentamethoxychalcone	Flavonoids	Chalcones	-
Wagp008838	3.75E+02	2.11E+02	3.74E+02	C20H22O7	[M+H]+	6'-Hydroxy-2,4,2',3',4'-Pentamethoxychalcone*	Flavonoids	Chalcones	-
Wagp007719	3.75E+02	2.11E+02	3.74E+02	C20H22O7	[M+H]+	6'-Hydroxy-3,4,2',3',4'-Pentamethoxychalcone	Flavonoids	Chalcones	114021-62-4

pmn001 400	3.75 E+02	2.43 E+02	3.76E+02	C19H20 O8	[M-H]-	2,3,5,4'-Tetrahydroxystilbene-2-O-xyloside	Others	Stilbene	-
Lmzn00 1983	3.75 E+02	1.35 E+02	3.76E+02	C16H24 O10	[M-H]-	D-Threo-guaiacylglycerol-7-O-β-D-glucoside	Others	Alcohol compounds	-
Cmrn00 1591	3.75 E+02	1.69 E+02	3.76E+02	C16H24 O10	[M-H]-	Mussaenosidic acid	Terpenoids	Monoterpenoids	82451-22-7
Zbsn012 113	3.79 E+02	2.95 E+02	3.80E+02	C21H32 O6	[M-H]-	Diacetoxy-[6]-gingerdiol	Others	Alcohol compounds	143615-75-2
Wafn00 3429	4.41 E+02	2.49 E+02	3.82E+02	C16H30 O10	[M+CH3COOH-H]-	3-Methylbutyl 6-O-(alpha-L-arabinopyranosyl)-beta-D-glucopyranoside	Others	Alcohol compounds	-
Wacn00 3456	3.83 E+02	1.27 E+02	3.84E+02	C16H16 O11	[M-H]-	2-O-Feruloylhydroxycitric Acid	Phenolic acids	Phenolic acids	-
Cmbn00 5456	3.83 E+02	1.47 E+02	3.84E+02	C18H24 O9	[M-H]-	Regaloside J	Phenolic acids	Phenolic acids	138771-99-0
Lmcp00 7265	3.89 E+02	3.59 E+02	3.88E+02	C20H20 O8	[M+H]+	3'-Demethylnobiletin*	Flavonoids	Flavones	112448-39-2
Wagp00 7545	3.89 E+02	3.59 E+02	3.88E+02	C20H20 O8	[M+H]+	3'-Hydroxy-3,4',5,7,8-Pentamethoxyflavone	Flavonoids	Flavones	-
Wagp00 8065	3.89 E+02	3.59 E+02	3.88E+02	C20H20 O8	[M+H]+	3-Hydroxy-3',4',5,5',7-Pentamethoxyflavone*	Flavonoids	Flavones	-

MWSslk185	3.89E+02	3.59E+02	3.88E+02	C20H20O8	[M+H]+	5-Demethylnobiletin; 5-Hydroxy-6,7,8,3',4'-Pentamethoxyflavone*	Flavonoids	Flavones	2174-59-6
Wagp010678	3.89E+02	3.59E+02	3.88E+02	C20H20O8	[M+H]+	5-Hydroxy-3,3',4',5',7-Pentamethoxyflavone*	Flavonoids	Flavones	5084-19-5
Wagp008700	3.89E+02	3.59E+02	3.88E+02	C20H20O8	[M+H]+	5-Hydroxy-3,6,7,4',5'-Pentamethoxyflavone	Flavonoids	Flavones	-
Wagp009533	3.89E+02	3.59E+02	3.88E+02	C20H20O8	[M+H]+	5-Hydroxy-6,7,3',4',5'-Pentamethoxyflavone	Flavonoids	Flavones	29215-55-2
Wagp010987	3.89E+02	3.59E+02	3.88E+02	C20H20O8	[M+H]+	Gossypetin 3,7,8,3',4'-pentamethyl ether*	Flavonoids	Flavonols	-
Hmln003169	3.87E+02	3.29E+02	3.88E+02	C21H24O7	[M-H]-	Trachelogenin	Lignans and Coumarins	Lignans	34209-69-3
pmb0766	3.90E+02	3.28E+02	3.89E+02	C21H15N3O5	[M+H]+	Azoxystrobin acid	Alkaloids	Alkaloids	1185255-09-7
Wagp008273	3.91E+02	2.41E+02	3.90E+02	C20H22O8	[M+H]+	3,6'-Dihydroxy-4,2',3',4',5'-Pentamethoxychalcone	Flavonoids	Chalcones	-
Lmdn003075	3.89E+02	1.67E+02	3.90E+02	C17H26O10	[M-H]-	3,4-Dihydroverbenalin	Terpenoids	Monoterpenoids	-
Wagp009968	3.95E+02	3.65E+02	3.94E+02	C22H18O7	[M+H]+	Justicidin A*	Lignans and Coumarins	Lignans	25001-57-4

Wagp01 0109	3.95 E+02	3.65 E+02	3.94E+02	C22H18 O7	[M+H]+	Justicidin C*	Lignans and Coumarins	Lignans	17803-12-2
Wbtn01 0362	3.93 E+02	3.93 E+02	3.94E+02	C23H22 O6	[M-H]-	6,8,12-trihydroxy-2,2-dimethyl-7-(3-methylbut-2-en-1-yl)-1,10-dioxatetraphen-5-one	Others	Ketone compounds	-
Lmhn00 2573	3.99 E+02	2.05 E+02	4.00E+02	C17H20 O11	[M-H]-	Sinapoylglucuronic acid	Phenolic acids	Phenolic acids	-
Wagp01 0653	4.03 E+02	3.73 E+02	4.02E+02	C21H22 O8	[M+H]+	3,5,6,7,3',4'-Hexamethoxyflavone	Flavonoids	Flavonols	1251-84-9
Wagp00 9079	4.03 E+02	3.73 E+02	4.02E+02	C21H22 O8	[M+H]+	3,5,6,7,8,4'-Hexamethoxyflavone	Flavonoids	Flavonols	34170-18-8
Wagp00 8324	4.03 E+02	3.73 E+02	4.02E+02	C21H22 O8	[M+H]+	5,6,2',3',4',6'-Hexamethoxyflavone	Flavonoids	Flavones	-
Hajp008 550	4.03 E+02	3.42 E+02	4.02E+02	C21H22 O8	[M+H]+	5,6,7,3',4',5'-hexamethoxyflavone	Flavonoids	Flavones	29043-07-0
mws004 3	4.03 E+02	3.73 E+02	4.02E+02	C21H22 O8	[M+H]+	Nobiletin (5,6,7,8,3',4'-Hexamethoxyflavone)	Flavonoids	Flavones	478-01-3
Hmln00 3529	4.01 E+02	2.69 E+02	4.02E+02	C18H26 O10	[M-H]-	Benzyl β-primeveroside	Others	Others	130622-31-0
Wagp00 9874	4.05 E+02	3.75 E+02	4.04E+02	C20H20 O9	[M+H]+	3',4'-Dihydroxy-5,6,7,8,5'-Pentamethoxyflavone*	Flavonoids	Flavones	-

Wagp00 9729	4.05 E+02	3.75 E+02	4.04E+02	C20H20 O9	[M+H]+	5,7-Dihydroxy-3,6,8,3',4'-Pentamethoxyflavone*	Flavonoids	Flavones	-
Jmbp00 5971	4.05 E+02	3.75 E+02	4.04E+02	C20H20 O9	[M+H]+	Gardenin C	Flavonoids	Flavones	29550-05-8
Lmjp00 3402	4.05 E+02	3.87 E+02	4.04E+02	C21H24 O8	[M+H]+	2'-Hydroxy-3,4,5,3',4',6'-hexameth-oxychalcone	Flavonoids	Chalcones	-
pmp000 114	4.05 E+02	2.41 E+02	4.04E+02	C21H24 O8	[M+H]+	5,6,7,8,3',4'-Hexamethoxyflavanone*	Flavonoids	Flavanones	-
Wagp01 1022	4.05 E+02	2.41 E+02	4.04E+02	C21H24 O8	[M+H]+	6'-Hydroxy-2,4,2',3',4',5'-Hexamethoxychalcone*	Flavonoids	Chalcones	-
Wagp00 9231	4.05 E+02	2.41 E+02	4.04E+02	C21H24 O8	[M+H]+	6'-Hydroxy-3,4,2',3',4',5'-Hexamethoxychalcone	Flavonoids	Chalcones	-
Yphn01 2690	4.07 E+02	4.07 E+02	4.08E+02	C25H28 O5	[M-H]-	6-Geranylnaringenin; Mimulone; Bonannione A	Flavonoids	Flavanones	97126-57-3
MWSN 0045	4.07 E+02	2.61 E+02	4.08E+02	C25H28 O5	[M-H]-	Kushenol A	Flavonoids	Other Flavonoids	99217-63-7
Wbmp0 06300	4.12 E+02	4.12 E+02	4.11E+02	C25H33 NO4	[M+H]+	1,2,9,10-tetraethoxy-6-methyl-5,6,6a,7-tetrahydro-4H-dibenzo[de,g]quinoline	Alkaloids	Alkaloids	-
Hmcp00 6520	4.13 E+02	3.95 E+02	4.12E+02	C25H48 O4	[M+H]+	1,3-Dihydroxypropan-2-yl 12-methyl-6-(6-methylheptyl)tridec-12-enoate	Others	Others	-

Yaap009385	4.15E+02	1.19E+02	4.14E+02	C24H30O6	[M+H]+	Myrtucommulone B	Others	Others	54247-23-3
Yxxn002325	4.15E+02	4.15E+02	4.16E+02	C19H28O10	[M-H]-	icariside D1	Others	Ketone compounds	-
Wasn006882	4.17E+02	1.21E+02	4.18E+02	C20H18O10	[M-H]-	2,5-Dibenzoylglucaric acid	Phenolic acids	Phenolic acids	-
Hmxp008844	4.19E+02	4.04E+02	4.18E+02	C21H22O9	[M+H]+	Gardenin A	Flavonoids	Flavones	21187-73-5
pma6218	4.19E+02	3.83E+02	4.18E+02	C21H22O9	[M+H]+	O-MethylNaringenin-8-C-arabinoside	Flavonoids	Flavanones	-
Zmhn001934	4.17E+02	2.55E+02	4.18E+02	C21H22O9	[M-H]-	Polygonimitin B	Flavonoids	Other Flavonoids	-
MWSN0046	4.19E+02	2.97E+02	4.20E+02	C25H24O6	[M-H]-	Morusin	Flavonoids	Other Flavonoids	62596-29-6
MWSN0049	4.23E+02	2.61E+02	4.24E+02	C25H28O6	[M-H]-	Sophoraflavanone G	Flavonoids	Flavanones	97938-30-2
MWSHY0024	4.25E+02	3.69E+02	4.24E+02	C25H28O6	[M+H]+	β-Mangostin	Flavonoids	Flavones	20931-37-7
zjbp120812	4.25E+02	3.65E+02	4.24E+02	C22H32O8	[M+H]+	Scutebarbolide C	Terpenoids	Ditepenoids	-

Qmdp112412	4.25E+02	2.61E+02	4.24E+02	C24H40O6	[M+H]+	(Rel 5S,6R,8R,9R,10S,13S,15S,16R)-6-Acetoxy-9,13;15,16 diepoxy-15,16-dimethoxylabdane	Terpenoids	Ditepenoids	-
mws1430	4.25E+02	9.51E+01	4.24E+02	C30H48O	[M+H]+	Lup-20(29)-en-3-one (Lupenone)	Terpenoids	Triterpene	1617-70-5
Hmcp005589	4.25E+02	2.03E+02	4.24E+02	C30H48O	[M+H]+	Olean-12-en-3-one (β-Amyrone)	Terpenoids	Triterpene	638-97-1
Hmcp005697	4.25E+02	1.91E+02	4.24E+02	C30H48O	[M+H]+	Olean-13(18)-en-3-one (δ-Amyrenone)	Terpenoids	Triterpene	20248-08-2
Hmcp005535	4.25E+02	1.91E+02	4.24E+02	C30H48O	[M+H]+	α-Amyrenone	Terpenoids	Triterpene	638-96-0
Wbmp006077	4.27E+02	4.27E+02	4.26E+02	C26H36NO4+	[M+H]+	1,10-dimethoxy-6,6-dimethyl-2,9-di(propan-2-yloxy)-5,6,6a,7-tetrahydro-4H-dibenzo[de,g]quinolin-6-ium	Alkaloids	Alkaloids	-
pmp000436	4.27E+02	4.09E+02	4.26E+02	C30H50O	[M+H]+	13-Methyl-27-norolean-14-en-3-ol (Taraxerol)	Terpenoids	Triterpene	127-22-0
pmf0085	4.27E+02	3.45E+02	4.26E+02	C30H50O	[M+H]+	9,19-Cyclolanost-24-en-3-ol (Cycloartenol)	Terpenoids	Triterpene	469-38-5
Yhhn008774	4.29E+02	4.29E+02	4.30E+02	C26H22O6	[M-H]-	hemerocallin	Phenolic acids	Phenolic acids	99305-33-6
pmp000117	4.33E+02	4.03E+02	4.32E+02	C22H24O9	[M+H]+	3,5,6,7,8,3',4'-Heptamethoxyflavone	Flavonoids	Flavonols	1178-24-1

Smnp001527	4.35E+02	2.55E+02	4.34E+02	C21H22O10	[M+H]+	5-hydroxyaloin	Quinones	Anthraquinone	-
Labn004865	4.33E+02	2.71E+02	4.34E+02	C21H22O10	[M-H]-	Hydroxy isoliquiritigenin glucoside	Flavonoids	Chalcones	-
pma0724	4.35E+02	2.85E+02	4.34E+02	C21H22O10	[M+H]+	Naringenin-6-C-Glucoside	Flavonoids	Flavanones	3682-03-9
HJN055	4.35E+02	2.73E+02	4.36E+02	C21H24O10	[M-H]-	Dihydrocharcone-4'-O-glucoside*	Flavonoids	Chalcones	-
Zbpn005555	4.35E+02	2.73E+02	4.36E+02	C21H24O10	[M-H]-	Phloretin-4'-O-glucoside (Trilobatin)*	Flavonoids	Chalcones	4192-90-9
MWSN0054	4.37E+02	1.61E+02	4.38E+02	C26H30O6	[M-H]-	Kurarinone	Flavonoids	Flavanones	34981-26-5
mws0355	4.41E+02	1.69E+02	4.42E+02	C22H18O10	[M-H]-	Catechin gallate*	Flavonoids	Flavanols	130405-40-2
mws1397	4.41E+02	1.69E+02	4.42E+02	C22H18O10	[M-H]-	Epicatechin gallate*	Flavonoids	Flavanols	1257-08-5
Lhdp112401	4.43E+02	1.37E+02	4.42E+02	C28H42O4	[M+H]+	Maesol	Others	Others	119766-98-2
pmp000438	4.43E+02	4.25E+02	4.42E+02	C30H50O2	[M+H]+	3,28-Dihydroxylup-20(29)-ene (Betulin)	Terpenoids	Triterpene	473-98-3

pmp000437	4.43E+02	4.25E+02	4.42E+02	C30H50O2	[M+H]+	D-Friedoolean-14-ene-3,28-diol (Myricadoil)	Terpenoids	Triterpene	17884-88-7
Wbsp013361	4.43E+02	4.25E+02	4.42E+02	C30H50O2	[M+H]+	serratenediol	Terpenoids	Triterpene	2239-24-9
MWStz079	4.45E+02	2.24E+02	4.44E+02	C27H28N2O4	[M+H]+	Aurantiamide acetate	Alkaloids	Alkaloids	56121-42-7
Lmlp001118	4.48E+02	2.86E+02	4.47E+02	C23H29NO8	[M+H]+	3'-Glucosyl-6,7-dihydroxy-N-methyl-benzyltetrahydroisoquinoline	Alkaloids	Isoquinoline alkaloids	-
mws0089	4.47E+02	2.85E+02	4.48E+02	C21H20O11	[M-H]-	Kaempferol-7-O-glucoside	Flavonoids	Flavonols	16290-07-6
MWSHY0132	4.49E+02	3.03E+02	4.48E+02	C21H20O11	[M+H]+	Quercetin-3-O-rhamnoside(Quercitrin)	Flavonoids	Flavonols	522-12-3
Wtmn009951	4.51E+02	4.51E+02	4.52E+02	C27H32O6	[M-H]-	3-(3,4-dihydroxybenzoyl)-4-hydroxy-6,6-dimethyl-1-(3-methylbut-2-en-1-yl)-7-(prop-1-en-2-yl)bicyclo[3.3.2]dec-3-ene-2,10-dione	Others	Ketone compounds	-
Yphn011804	4.53E+02	4.53E+02	4.54E+02	C26H30O7	[M-H]-	3'-O-Methyldiplacol	Flavonoids	Flavanonols	-
Lmmn009550	4.53E+02	4.53E+02	4.54E+02	C30H46O3	[M-H]-	3-Oxo-9,19-cyclolanost-24-en-26-oic acid (Mangiferonic acid)*	Terpenoids	Triterpene	13878-90-5
MWSmce134	4.53E+02	4.53E+02	4.54E+02	C30H46O3	[M-H]-	3-Oxolup-20(29)-en-28-oic acid (Betulonic acid)*	Terpenoids	Triterpene	4481-62-3

Lmzn006795	4.53E+02	4.53E+02	4.54E+02	C30H46O3	[M-H]-	3-Oxours-12-en-28-oic acid (Ursonic acid)*	Terpenoids	Triterpene	6246-46-4
Yshs000050	4.55E+02	4.37E+02	4.54E+02	C30H46O3	[M+H]+	Lupane-20(29)-en-3-on-28-oic acid	Terpenoids	Triterpene	-
Hmbn004734	4.55E+02	4.55E+02	4.56E+02	C29H44O4	[M-H]-	3,23-Dihydroxy-30-noroleana-12,20(29)-dien-28-oic acid (30-Norhederagenin)	Terpenoids	Triterpene	117654-06-5
Wbsp011256	4.57E+02	4.57E+02	4.56E+02	C30H48O3	[M+H]+	16-oxo-21β-hydroxyserrat-14-en-3α-yl acetate	Terpenoids	Triterpene	-
Lmmn009170	4.55E+02	4.55E+02	4.56E+02	C30H48O3	[M-H]-	3-Hydroxy-9,19-cyclolanost-24-en-26-oic acid (Mangiferolic acid)	Terpenoids	Triterpene	4184-34-3
Lmmn008971	4.55E+02	4.55E+02	4.56E+02	C30H48O3	[M-H]-	3-Hydroxycycloarta-24-ene-26-oic acid (Isomangiferolic acid)*	Terpenoids	Triterpene	13878-92-7
MWSmce052	4.55E+02	4.55E+02	4.56E+02	C30H48O3	[M-H]-	3-Hydroxyurs-12-en-28-oic acid (3-Epiursolic acid)*	Terpenoids	Triterpene	989-30-0
mws4053	4.55E+02	4.55E+02	4.56E+02	C30H48O3	[M-H]-	3-Hydroxyurs-12-en-28-oic acid (Ursolic acid)*	Terpenoids	Triterpene	77-52-1
MWSHC2016	4.57E+02	1.69E+02	4.58E+02	C22H18O11	[M-H]-	Epigallocatechin-3-O-gallate	Flavonoids	Flavanols	989-51-5
Lasp010273	4.59E+02	2.03E+02	4.58E+02	C30H34O4	[M+H]+	Sophoranochromene	Flavonoids	Flavanones	-

Zmjp013616	4.59E+02	4.23E+02	4.58E+02	C30H50O3	[M+H]+	12,13-Dihydroursolic acid	Terpenoids	Triterpene	-
mws1086	4.57E+02	3.75E+02	4.58E+02	C30H50O3	[M-H]-	Olean-12-ene-3,22,23-triol (Soyasapogenol B)	Terpenoids	Triterpene	595-15-3
Wbsp008859	4.59E+02	4.23E+02	4.58E+02	C30H50O3	[M+H]+	Serratriol(Serrat-14-en-3β,21α,24-triol)	Terpenoids	Triterpene	13956-52-0
pmb2654	4.60E+02	1.18E+02	4.61E+02	C19H27NO12	[M-H]-	Anthranilate-1-O-Sophoroside	Phenolic acids	Phenolic acids	-
Lmpn006208	4.61E+02	3.15E+02	4.62E+02	C22H22O11	[M-H]-	8-Methoxykaempferol-7-O-rhamnoside	Flavonoids	Flavonols	-
Zbrn006627	4.61E+02	2.99E+02	4.62E+02	C22H22O11	[M-H]-	Chrysoeriol-5-O-glucoside	Flavonoids	Flavones	-
WaYn005032	4.61E+02	3.15E+02	4.62E+02	C22H22O11	[M-H]-	Gentisic acid 2-O-(6''-O-p-coumaroyl)Glucoside	Phenolic acids	Phenolic acids	-
pmn001468	4.61E+02	1.37E+02	4.62E+02	C19H26O13	[M-H]-	Sibiricose A3	Phenolic acids	Phenolic acids	139726-39-9
mws0091	4.63E+02	3.00E+02	4.64E+02	C21H20O12	[M-H]-	Quercetin-3-O-glucoside (Isoquercitrin)	Flavonoids	Flavonols	482-35-9
mws0856	4.63E+02	3.01E+02	4.64E+02	C21H20O12	[M-H]-	Quercetin-4'-O-glucoside (Spiraeoside)*	Flavonoids	Flavonols	20229-56-5

ZBN0352	4.63E+02	3.01E+02	4.64E+02	C21H20O12	[M-H]-	Quercetin-5-O-β-D-glucoside*	Flavonoids	Flavonols	-
mws1329	4.63E+02	3.01E+02	4.64E+02	C21H20O12	[M-H]-	Quercetin-7-O-glucoside*	Flavonoids	Flavonols	491-50-9
pme1598	4.63E+02	3.01E+02	4.64E+02	C22H24O11	[M-H]-	Hesperetin-5-O-glucoside	Flavonoids	Flavanones	69651-80-5
pmb0504	4.69E+02	1.77E+02	4.68E+02	C21H32N4O8	[M+H]+	N-(4'-O-glycosyl)-feruloyl agmatine	Alkaloids	Phenolamine	-
pmn001704	4.69E+02	4.23E+02	4.70E+02	C30H46O4	[M-H]-	2,3-Dihydroxy-5(6),12(13)-diene-ursolic acid*	Terpenoids	Triterpene	-
Hmjn008136	4.69E+02	4.69E+02	4.70E+02	C30H46O4	[M-H]-	2,3-Dihydroxyoleana-11,13(18)-dien-28-oic acid (Camaldulenic acid)	Terpenoids	Triterpene	71850-15-2
Lmsn012627	4.69E+02	4.23E+02	4.70E+02	C30H46O4	[M-H]-	2,3-Dihydroxyurs-12,18-dien-28-oic acid*	Terpenoids	Triterpene	-
Zmcn009787	4.69E+02	4.39E+02	4.70E+02	C30H46O4	[M-H]-	23-Hydroxy-3-oxoolean-12-en-28-oic acid (Hederagonic acid)	Terpenoids	Triterpene	466-01-3
Lmsn013282	4.69E+02	4.07E+02	4.70E+02	C30H46O4	[M-H]-	3-Oxo-19-hydroxyurs-12-en-28-oic acid (Pomonic acid)	Terpenoids	Triterpene	13849-90-6
Yhjp010205	4.71E+02	4.71E+02	4.70E+02	C30H46O4	[M+H]+	Virgatic acid	Terpenoids	Triterpene	14356-51-5

Lmmn009932	4.69E+02	4.69E+02	4.70E+02	C31H50O3	[M-H]-	3-Hydroxy-24-methylene-9,19-cyclolanostan-26-oic acid (Ambolic acid)	Terpenoids	Triterpene	13878-93-8
ZBN0054	4.71E+02	3.19E+02	4.72E+02	C23H20O11	[M-H]-	Epigallocatechin-3-O-(3-O-methyl)gallate	Flavonoids	Flavanols	83104-87-4
Yshs000051	4.73E+02	1.65E+02	4.72E+02	C29H44O5	[M+H]+	6-Hydroxy-3,20-dioxo-30-norlupane-28-oic acid	Terpenoids	Triterpene	-
pmn001427	4.71E+02	4.71E+02	4.72E+02	C30H48O4	[M-H]-	16,23:16,30-Diepoxydammar-24-ene-3,20-diol (Jujubogenin)*	Terpenoids	Triterpene	54815-36-0
Smpn011792	4.71E+02	4.71E+02	4.72E+02	C30H48O4	[M-H]-	2,3-Dihydroxy-12-ursen-28-oic acid*	Terpenoids	Triterpene	-
Lmzn106284	4.71E+02	4.71E+02	4.72E+02	C30H48O4	[M-H]-	2,3-Dihydroxylup-20(29)-en-28-oic acid (Alphitolic acid)*	Terpenoids	Triterpene	19533-92-7
pmn001706	4.71E+02	4.71E+02	4.72E+02	C30H48O4	[M-H]-	2,3-Dihydroxyolean-12-en-28-oic acid (2-Hydroxyoleanolic acid)*	Terpenoids	Triterpene	26707-60-8
Zmpn008194	4.71E+02	4.71E+02	4.72E+02	C30H48O4	[M-H]-	2,3-Dihydroxyurs-12-en-28-oic acid (Corosolic acid)*	Terpenoids	Triterpene	4547-24-4
mws1610	4.71E+02	4.71E+02	4.72E+02	C30H48O4	[M-H]-	2,3-Dihydroxyurs-12-en-29-oic acid (Maslinic acid)*	Terpenoids	Triterpene	4373-41-5
Lmzn006284	4.71E+02	4.71E+02	4.72E+02	C30H48O4	[M-H]-	2-Hydroxyursolic acid*	Terpenoids	Triterpene	-

Lmgn013947	4.71E+02	4.71E+02	4.72E+02	C30H48O4	[M-H]-	2α,3α-Dihydroxyolean-12-en-28-oic acid	Terpenoids	Triterpene	26563-68-8
Hmbn005207	4.71E+02	4.71E+02	4.72E+02	C30H48O4	[M-H]-	3,23-Dihydroxyolean-12-en-28-oic acid (Hederagenin)*	Terpenoids	Triterpene	465-99-6
pmn001705	4.71E+02	4.71E+02	4.72E+02	C30H48O4	[M-H]-	3,24-Dihydroxy-17,21-semiacetal-12(13)oleanolic fruit*	Terpenoids	Triterpene	-
Lmsn012425	4.71E+02	2.77E+02	4.72E+02	C30H48O4	[M-H]-	3,7-Dihydroxyolean-12-en-28-oic acid (Rubusic acid)	Terpenoids	Triterpene	23984-26-1
Ylyn012627	4.71E+02	4.71E+02	4.72E+02	C30H48O4	[M-H]-	salaspermic acid	Terpenoids	Triterpene	71247-78-4
pmb1912	4.74E+02	3.27E+02	4.73E+02	C20H23N7O7	[M+H]+	10-Formyltetrahydrofolic Acid	Alkaloids	Alkaloids	2800-34-2
Zbqn008291	4.73E+02	4.73E+02	4.74E+02	C30H50O4	[M-H]-	3,13,15-Trihydroxyoleanane-12-one	Terpenoids	Triterpene	-
pmn001727	4.74E+02	3.12E+02	4.75E+02	C24H29NO9	[M-H]-	N-(4-O-(Glucosyl)-E-feruloyl)-tyramine	Alkaloids	Phenolamine	-
Lmfn003760	4.77E+02	3.01E+02	4.78E+02	C21H18O13	[M-H]-	Quercetin-4'-O-glucuronide*	Flavonoids	Flavonols	201463-36-7
ZBN0137	4.77E+02	3.01E+02	4.78E+02	C21H18O13	[M-H]-	Quercetin-5-O-glucuronide*	Flavonoids	Flavonols	-

Wayn004603	4.77E+02	3.01E+02	4.78E+02	C21H18O13	[M-H]-	Tricetin 3'-glucuronide*	Flavonoids	Flavones	-
pmn001525	4.77E+02	1.69E+02	4.78E+02	C23H26O11	[M-H]-	3,5-Digalloylshikimic acid	Phenolic acids	Phenolic acids	95753-52-9
Wbwp013975	4.81E+02	3.55E+02	4.80E+02	C30H40O5	[M+H]+	lancilactone A	Terpenoids	Terpene	218915-15-2
Wbmp007680	4.84E+02	4.84E+02	4.83E+02	C29H41NO5	[M+H]+	5,6,6a,7-tetrahydro-1,2,9,10-tetraethoxy-6-n-pentyl-4H-dibenzo[de,g]quinolin-4-ol	Alkaloids	Alkaloids	-
pmp001090	4.85E+02	3.81E+02	4.84E+02	C26H28O9	[M+H]+	Evodol	Terpenoids	Triterpene	22318-10-1
Wbsp000333	4.85E+02	4.85E+02	4.84E+02	C31H48O4	[M+H]+	16-oxo-3α,21β-dihydroxyserrat-14-en-24-oic acid	Terpenoids	Terpene	-
pmb2723	4.85E+02	3.23E+02	4.86E+02	C21H26O13	[M-H]-	4-Hydroxycoumarin di-glucoside	Lignans and Coumarins	Coumarins	-
Zjlp100904	4.87E+02	3.95E+02	4.86E+02	C30H46O5	[M+H]+	3-Hydroxyolean-12-ene-28,30-dioic acid (Spergulagenic acid)	Terpenoids	Triterpene	18671-48-2
pmp000269	4.89E+02	4.07E+02	4.88E+02	C30H48O5	[M+H]+	2,3,19-Trihydroxyolean-12-en-28-oic acid (Arjunic acid)	Terpenoids	Triterpene	31298-06-3
pmn001591	4.87E+02	4.69E+02	4.88E+02	C30H48O5	[M-H]-	3,19,23-Trihydroxyurs-12-en-28-oic acid (Rutundic acid)	Terpenoids	Triterpene	20137-37-5

Wbsp006529	4.89E+02	4.89E+02	4.88E+02	C30H48O5	[M+H]+	Lycoclavanin	Terpenoids	Triterpene	27832-90-2
Yyhn011941	4.87E+02	4.87E+02	4.88E+02	C30H48O5	[M-H]-	acerogenic acid	Terpenoids	Triterpene	29038-42-4
Wagp004812	4.91E+02	3.29E+02	4.90E+02	C24H26O11	[M+H]+	Salvigenin 5-O-glucoside	Flavonoids	Flavones	-
Wbmp003594	4.92E+02	4.92E+02	4.91E+02	C27H41NO7	[M+H]+	6-acetyldelpheline	Alkaloids	Alkaloids	-
Wasn005803	4.91E+02	3.15E+02	4.92E+02	C23H24O12	[M-H]-	Protocatechuic acid 4-O-(6"-O-Feruloyl)Glucoside	Phenolic acids	Phenolic acids	-
Cmqp007543	4.95E+02	1.81E+02	4.94E+02	C24H30O11	[M+H]+	Harpagoside	Terpenoids	Monoterpenoids	19210-12-9
Zbzn010988	4.99E+02	4.57E+02	5.00E+02	C29H40O7	[M-H]-	Ardisianone E	Quinones	Quinones	-
Lmhn010561	4.99E+02	4.55E+02	5.00E+02	C30H44O6	[M-H]-	Kadcoccitone B	Terpenoids	Triterpene	1415976-57-6
Lmsn010222	5.03E+02	4.41E+02	5.04E+02	C30H48O6	[M-H]-	1,2,3-Trihydroxy-19-oxo-18,19-seco-urs-11,13(18)-dien-28-oic acid	Terpenoids	Triterpene	-
Lmsn009824	5.03E+02	4.41E+02	5.04E+02	C30H48O6	[M-H]-	2,3,19,23-Tetrahydroxyolean-12-en-28-oic acid	Terpenoids	Triterpene	55306-03-1

Smpn009074	5.03E+02	5.03E+02	5.04E+02	C30H48O6	[M-H]-	2,3,19,23-Tetrahydroxyurs-12-en-28-oic acid	Terpenoids	Triterpene	-
pmp000499	5.07E+02	4.36E+02	5.06E+02	C31H42N2O4	[M+H]+	Nigramide M	Alkaloids	Piperidine alkaloids	-
Lmmn007259	5.17E+02	4.89E+02	5.18E+02	C30H30O8	[M-H]-	Gossypol	Terpenoids	Sesquiterpenoids	303-45-7
Lazp003665	5.25E+02	5.25E+02	5.24E+02	C25H32O12	[M+H]+	Nagilactone C glucoside	Terpenoids	Ditepenoids	-
ZBN0228	5.67E+02	3.29E+02	5.68E+02	C29H28O12	[M-H]-	Tricin-4'-O-[β-guaiacyl-(9"-O-acetyl)glycerol]ether	Flavonoids	Flavones	-
ZBN0419	5.79E+02	2.89E+02	5.80E+02	C30H28O12	[M-H]-	Gambiriin A1	Tannins	Tannin	76250-49-2
Wtmn002164	5.79E+02	5.79E+02	5.80E+02	C27H32O14	[M-H]-	3,4-dimethoxy-2-[(3,4,5-trihydroxy-6-{[(3,4,5-trihydroxy-6-methyloxan-2-yl)oxy]methyl}oxan-2-yl)oxy]xanthen-9-one	Flavonoids	Other Flavonoids	-
mws0046	5.79E+02	2.71E+02	5.80E+02	C27H32O14	[M-H]-	Naringenin-7-O-Neohesperidoside(Naringin)*	Flavonoids	Flavanones	10236-47-2
mws1066	5.79E+02	2.71E+02	5.80E+02	C27H32O14	[M-H]-	Naringenin-7-O-Rutinoside(Narirutin)*	Flavonoids	Flavanones	14259-46-2
Xmzn007677	5.80E+02	5.06E+02	5.81E+02	C33H35N5O5	[M-H]-	Ergotamine	Alkaloids	Plumerane	113-15-5

pmb0492	5.84E+02	3.25E+02	5.83E+02	C34H37N3O6	[M+H]+	N',N'',N'''-p-Coumaroyl-cinnamoyl-caffeoyl spermidine	Alkaloids	Phenolamine	-
ZBN0043	5.93E+02	2.85E+02	5.94E+02	C30H26O13	[M-H]-	Kaempferol-3-O-(2''-p-Coumaroyl)galactoside*	Flavonoids	Flavonols	-
mws1290	5.93E+02	2.85E+02	5.94E+02	C30H26O13	[M-H]-	Kaempferol-3-O-(6''-p-Coumaroyl)glucoside (Tiliroside)*	Flavonoids	Flavonols	20316-62-5
MWSslk252	5.93E+02	2.85E+02	5.94E+02	C28H34O14	[M-H]-	Didymin (Isosakuranetin-7-O-rutinoside)*	Flavonoids	Flavanones	14259-47-3
mws0791	5.93E+02	2.85E+02	5.94E+02	C28H34O14	[M-H]-	Poncirin (Isosakuranetin-7-O-neohesperidoside)*	Flavonoids	Flavanones	14941-08-3
Lmtp004044	5.97E+02	3.03E+02	5.96E+02	C26H28O16	[M+H]+	Quercetin-3-O-apiosyl(1→2)galactoside	Flavonoids	Flavonols	-
mws1519	5.95E+02	2.87E+02	5.96E+02	C27H32O15	[M-H]-	Eriodictyol-7-O-Rutinoside (Eriocitrin)	Flavonoids	Flavanones	13463-28-0
MWSHY0190	6.09E+02	3.01E+02	6.08E+02	C28H32O15	[M+H]+	Diosmetin-7-O-rutinoside (Diosmin)	Flavonoids	Flavones	520-27-4
ZBN0306	6.09E+02	3.01E+02	6.10E+02	C27H30O16	[M-H]-	Quercetin-3-O-(4''-O-glucosyl)rhamnoside*	Flavonoids	Flavonols	59262-54-3
ZBN0082	6.09E+02	3.01E+02	6.10E+02	C27H30O16	[M-H]-	Quercetin-3-O-glucoside-7-O-rhamnoside*	Flavonoids	Flavonols	-

ZBN0146	6.09E+02	3.01E+02	6.10E+02	C27H30O16	[M-H]-	Quercetin-3-O-neohesperidoside*	Flavonoids	Flavonols	29662-79-1
pmn001583	6.09E+02	3.00E+02	6.10E+02	C27H30O16	[M-H]-	Quercetin-3-O-robinobioside	Flavonoids	Flavonols	52525-35-6
mws0059	6.09E+02	3.01E+02	6.10E+02	C27H30O16	[M-H]-	Quercetin-3-O-rutinoside (Rutin)*	Flavonoids	Flavonols	153-18-4
mws0036	6.09E+02	3.01E+02	6.10E+02	C28H34O15	[M-H]-	Hesperetin-7-O-rutinoside (Hesperidin)	Flavonoids	Flavanones	520-26-3
ZBN0014	6.21E+02	2.69E+02	6.22E+02	C31H26O14	[M-H]-	Apigenin-7-O-(6''-feruloyl)glucuronide	Flavonoids	Flavones	-
Lssp210167	6.25E+02	4.63E+02	6.24E+02	C32H48O12	[M+H]+	2α,5α,14β-Triacetoxy-10β-O-(B-D glucopyranosy)taxa-4(20),11-diene	Others	Others	-
MWSHY0162	6.27E+02	3.03E+02	6.26E+02	C27H30O17	[M+H]+	Quercetin-3-O-sophoroside (Baimaside)	Flavonoids	Flavonols	18609-17-1
Wbkn011324	6.65E+02	6.05E+02	6.66E+02	C39H54O9	[M-H]-	Bruceajavanone B	Terpenoids	Triterpene	-
ZBN0255	6.81E+02	3.29E+02	6.82E+02	C33H30O16	[M-H]-	Tricin-7-O-(2''-feruloyl)glucuronide	Flavonoids	Flavones	-
Lhhp102402	7.51E+02	4.37E+02	7.50E+02	C40H62O13	[M+H]+	6-O-Acetyl-3-O-(4-O-malonyl)-xylosylcycloastragenol	Terpenoids	Triterpene Saponin	-

Table S2. Differential metabolites and their information.

Compounds	Class I	Class II	VIP	P-value	FDR	Fold_Change	Log2FC	Type
Methylephedrine	Alkaloids	Alkaloids	1.04E+00	2.57E-08	8.93E-06	5.98E-02	-4.06E+00	down
Urolithin D	Flavonoids	Other Flavonoids	1.04E+00	2.67E-08	8.93E-06	7.47E+00	2.90E+00	up
glycoric acid	Others	Others	1.04E+00	2.86E-08	8.93E-06	5.14E+00	2.36E+00	up
3-hydroxy-1-methylpyrrolidin-2-one*	Alkaloids	Pyrrole alkaloids	1.04E+00	8.63E-08	2.02E-05	2.28E+01	4.51E+00	up
Sideretin (5,7,8-Trihydroxy-6-methoxycoumarin)	Lignans and Coumarins	Coumarins	1.04E+00	2.01E-07	3.77E-05	4.30E+00	2.11E+00	up
Valerine	Alkaloids	Alkaloids	1.03E+00	4.36E-07	6.82E-05	2.10E+01	4.40E+00	up
Cadaverine	Alkaloids	Alkaloids	1.04E+00	5.30E-07	7.04E-05	1.06E+01	3.41E+00	up
Caffeic acid	Phenolic acids	Phenolic acids	1.04E+00	6.00E-07	7.04E-05	3.75E+00	1.91E+00	up
Virgatic acid	Terpenoids	Triterpene	1.03E+00	7.02E-07	7.11E-05	7.45E-02	-3.75E+00	down
L-Carnitine	Alkaloids	Alkaloids	1.04E+00	8.63E-07	7.36E-05	2.02E+01	4.34E+00	up
Polygonimitin B	Flavonoids	Other Flavonoids	1.04E+00	1.80E-06	1.41E-04	2.00E-01	-2.32E+00	down

4(3H)-Quinazolinone	Alkaloids	Alkaloids	1.03E+00	2.30E-06	1.58E-04	3.17E-03	-8.30E+00	down
Tangeretin (4',5,6,7,8-Pentamethoxyflavone)*	Flavonoids	Flavones	1.04E+00	2.45E-06	1.58E-04	1.85E-01	-2.43E+00	down
2-Amino-4,5-dihydro-1H-imidazole-4-acetic acid	Alkaloids	Alkaloids	1.04E+00	2.62E-06	1.58E-04	7.06E+00	2.82E+00	up
Isoquinoline	Alkaloids	Isoquinoline alkaloids	1.04E+00	2.69E-06	1.58E-04	1.28E-01	-2.97E+00	down
Sinensetin (5,6,7,3',4'-pentamethoxyflavone)*	Flavonoids	Flavones	1.04E+00	3.19E-06	1.76E-04	5.36E+00	2.42E+00	up
Choline	Alkaloids	Alkaloids	1.04E+00	4.08E-06	1.85E-04	2.22E+01	4.47E+00	up
2-Amino-3-methoxybenzoic acid	Phenolic acids	Phenolic acids	1.04E+00	4.09E-06	1.85E-04	5.84E+00	2.55E+00	up

2'-Hydroxy-3,4,5,3',4',6'-hexameth-oxychalcone	Flavonoids	Chalcones	1.04E+00	4.13E-06	1.85E-04	5.33E-02	-4.23E+00	down
2-Hydroxy-4-methyl-3-undecanoyloxypentanoic acid methyl ester	Others	Others	1.04E+00	4.49E-06	1.85E-04	1.21E-01	-3.05E+00	down
5,7,2'-Trihydroxy-8-methoxyflavone; Scutevulin*	Flavonoids	Flavones	1.03E+00	4.53E-06	1.85E-04	9.29E-02	-3.43E+00	down
Kadcoccitone B	Terpenoids	Triterpene	1.03E+00	4.54E-06	1.85E-04	1.11E-01	-3.18E+00	down
4,8-Dihydroxyquinoline-2-carboxylic acid	Alkaloids	Quinoline alkaloids	1.04E+00	5.66E-06	2.14E-04	1.82E+01	4.18E+00	up
Norharmane; Beta-Carboline	Alkaloids	Plumerane	1.04E+00	5.71E-06	2.14E-04	8.84E-02	-3.50E+00	down

4-Nitrophenol	Others	Others	1.04E+00	7.99E-06	2.65E-04	3.12E+00	1.64E+00	up
N-Benzylmethylene isomethylamine	Alkaloids	Alkaloids	1.04E+00	7.99E-06	2.65E-04	3.08E+01	4.95E+00	up
4-Methyl-5-thiazoleethanol	Others	Others	1.04E+00	8.07E-06	2.65E-04	7.93E-03	-6.98E+00	down
6-Methoxy-2-(2-phenylethyl)chromone	Others	Chromone	1.04E+00	8.18E-06	2.65E-04	1.27E-01	-2.97E+00	down
2,3,5,4'-Tetrahydroxystilbene-2-O-xyloside	Others	Stilbene	1.03E+00	9.76E-06	2.94E-04	6.59E+00	2.72E+00	up
Baptifoline	Alkaloids	Quinorisidine alkaloids	1.04E+00	1.03E-05	2.94E-04	2.48E+02	7.95E+00	up
Nicotianamine	Alkaloids	Alkaloids	1.04E+00	1.03E-05	2.94E-04	6.32E+00	2.66E+00	up
2,3-Dihydroxyurs-12-en-29-oic acid (Maslinic acid)*	Terpenoids	Triterpene	1.02E+00	1.03E-05	2.94E-04	9.63E-02	-3.38E+00	down

2,6-Dimethyl-7-octene-2,3,6-triol	Terpenoids	Monoterpenoids	1.04E+00	1.16E-05	3.21E-04	4.69E-01	-1.09E+00	down
Imidazole-4-Acetic Acid	Alkaloids	Alkaloids	1.04E+00	1.39E-05	3.47E-04	3.06E+00	1.61E+00	up
5-hydroxy-anthraquinone-2-carboxylic acid	Quinones	Anthraquinone	1.04E+00	1.40E-05	3.47E-04	1.27E-01	-2.97E+00	down
2(3H)-Benzothiazolone	Alkaloids	Alkaloids	1.04E+00	1.44E-05	3.47E-04	8.43E-02	-3.57E+00	down
3-(3',5'-Dimethoxy-4'-hydroxy-benzylidene')-2-indolinone	Alkaloids	Plumerane	1.04E+00	1.56E-05	3.47E-04	2.75E+01	4.78E+00	up
Pterolactam*	Alkaloids	Pyrrole alkaloids	1.04E+00	1.57E-05	3.47E-04	2.13E+01	4.41E+00	up
Rugosic acid A	Terpenoids	Sesquiterpenoids	1.04E+00	1.58E-05	3.47E-04	6.03E+00	2.59E+00	up
3,4-Dimethoxyphenyl acetic acid	Phenolic acids	Phenolic acids	1.03E+00	1.59E-05	3.47E-04	2.67E-01	-1.91E+00	down

N-ethylcytisine	Alkaloids	Piperidine alkaloids	1.04E+00	1.64E-05	3.50E-04	1.16E+01	3.53E+00	up
Methyl-3-(3-hydroxyphenyl)Propionate	Phenolic acids	Phenolic acids	1.04E+00	1.73E-05	3.60E-04	2.34E-02	-5.42E+00	down
Coumalic acid	Phenolic acids	Phenolic acids	1.04E+00	1.76E-05	3.60E-04	2.65E-01	-1.92E+00	down
Retronecine	Alkaloids	Pyrrole alkaloids	1.01E+00	1.85E-05	3.69E-04	1.62E+01	4.02E+00	up
4-[2-(1-methylethyl)aminoethyl]phenol	Alkaloids	Quinoline alkaloids	1.03E+00	1.99E-05	3.89E-04	1.85E-01	-2.44E+00	down
β-Cannabispiranol	Terpenoids	Sesquiterpenoids	1.04E+00	2.04E-05	3.90E-04	3.54E-01	-1.50E+00	down
Anthranilate-1-O-Sophoroside	Phenolic acids	Phenolic acids	1.03E+00	2.11E-05	3.92E-04	4.74E+00	2.24E+00	up
1,2,3,4-tetrahydronorharman-l-one	Alkaloids	Alkaloids	1.04E+00	2.13E-05	3.92E-04	2.51E-02	-5.32E+00	down
Oleuropeic acid	Terpenoids	Monoterpenoids	1.03E+00	2.22E-05	4.00E-04	1.47E-01	-2.76E+00	down
Gallic acid	Phenolic acids	Phenolic acids	1.04E+00	2.31E-05	4.09E-04	2.15E+00	1.11E+00	up

Quercetin-3,4'-Dimethyl Ether	Flavonoids	Flavonols	1.04E+00	2.40E-05	4.17E-04	1.16E-02	-6.43E+00	down
6'-Hydroxy-2,4,2',3',4'-Pentamethoxychalcone*	Flavonoids	Chalcones	1.04E+00	2.51E-05	4.28E-04	1.14E-01	-3.13E+00	down
2,4-Dihydroxybenzoic acid	Phenolic acids	Phenolic acids	1.00E+00	2.68E-05	4.48E-04	3.46E+01	5.11E+00	up
Hydrocinnamic acid	Phenolic acids	Phenolic acids	1.04E+00	2.72E-05	4.48E-04	1.35E-01	-2.89E+00	down
Lindenenol K	Terpenoids	Sesquiterpenoids	1.04E+00	2.81E-05	4.54E-04	5.04E+00	2.33E+00	up
2-O-Caffeoylhydroxycitric Acid	Phenolic acids	Phenolic acids	1.03E+00	2.86E-05	4.54E-04	4.81E+00	2.27E+00	up
4-Aminobenzoic acid	Phenolic acids	Phenolic acids	1.04E+00	2.91E-05	4.55E-04	2.62E-01	-1.93E+00	down
Xanthatin	Terpenoids	Sesquiterpenoids	1.04E+00	3.25E-05	5.00E-04	3.32E-01	-1.59E+00	down
Vanillin; 4-Hydroxy-3-	Others	Aldehyde compounds	1.03E+00	3.32E-05	5.02E-04	4.92E-02	-4.34E+00	down

Methoxybenzaldehyde								
2,3-Dihydroxypropanal	Others	Aldehyde compounds	1.04E+00	3.44E-05	5.12E-04	9.69E+00	3.28E+00	up
1-hydroxyacridone	Alkaloids	Quinorisidine alkaloids	1.04E+00	3.50E-05	5.12E-04	4.31E-02	-4.53E+00	down
Deoxymutaaspergillic acid	Alkaloids	Alkaloids	1.04E+00	3.57E-05	5.12E-04	1.88E-04	-1.24E+01	down
Maplexin B (4-O-Galloyl-1,5-Anhydro-D-Glucitol)	Tannins	Tannin	1.04E+00	3.61E-05	5.12E-04	7.46E+00	2.90E+00	up
4-Hydroxyquinoline	Alkaloids	Quinoline alkaloids	1.04E+00	3.70E-05	5.12E-04	4.10E-02	-4.61E+00	down
Eremofukinone	Terpenoids	Sesquiterpenoids	1.04E+00	3.71E-05	5.12E-04	3.45E-01	-1.54E+00	down
Betaine	Alkaloids	Alkaloids	1.03E+00	3.85E-05	5.12E-04	2.17E+01	4.44E+00	up
Stachydrine	Alkaloids	Pyrrole alkaloids	1.04E+00	3.86E-05	5.12E-04	1.09E+01	3.45E+00	up

1,10-dimethoxy-6,6-dimethyl-2,9-di(propan-2-yloxy)-5,6,6a,7-tetrahydro-4H-dibenzo[de,g]quinolin-6-ium	Alkaloids	Alkaloids	1.01E+00	3.88E-05	5.12E-04	1.24E-01	-3.02E+00	down
3,4',5,6,7-Pentamethoxyflavone	Flavonoids	Flavonols	1.04E+00	3.99E-05	5.19E-04	1.69E-01	-2.56E+00	down
2'-Hydroxygenistein	Flavonoids	Isoflavones	1.04E+00	4.25E-05	5.37E-04	6.49E+00	2.70E+00	up
lycopsamine N-oxide	Alkaloids	Pyrrole alkaloids	1.01E+00	4.32E-05	5.37E-04	8.45E+00	3.08E+00	up
N-benzylformamide	Alkaloids	Alkaloids	1.04E+00	4.39E-05	5.37E-04	9.50E+01	6.57E+00	up
3,5-Dihydro-2H-Furo[3,2-C]Quinolin-4-One*	Alkaloids	Quinoline alkaloids	1.04E+00	4.40E-05	5.37E-04	1.33E+02	7.05E+00	up

1-Pyrroline-4-hydroxy-2-carboxylic acid	Alkaloids	Pyrrole alkaloids	1.04E+00	4.41E-05	5.37E-04	7.56E+00	2.92E+00	up
Trans-dehydrorosinone	Steroids	Steroid	1.04E+00	4.60E-05	5.53E-04	1.32E-01	-2.92E+00	down
Myrtucommulone B	Others	Others	1.04E+00	4.96E-05	5.74E-04	1.00E-01	-3.32E+00	down
6'-Hydroxy-4,2',3',4'-Tetramethoxychalcone	Flavonoids	Chalcones	1.03E+00	4.96E-05	5.74E-04	2.87E-01	-1.80E+00	down
N-Acetylcadaverine	Alkaloids	Alkaloids	1.04E+00	5.09E-05	5.82E-04	2.06E+01	4.36E+00	up
3,5,7,3'4'-Pentamethoxyflavone	Flavonoids	Flavonols	1.04E+00	5.27E-05	5.96E-04	4.93E+00	2.30E+00	up
[4]-Gingerol	Phenolic acids	Phenolic acids	1.04E+00	5.54E-05	6.12E-04	2.11E-02	-5.56E+00	down
2-(2-pent-4-enoxyethoxy)ethyl hydrogen sulfate	Others	Others	1.02E+00	5.89E-05	6.42E-04	6.46E+00	2.69E+00	up

Heptane-1,7-diol sulfate	Others	Others	1.03E+00	6.06E-05	6.54E-04	2.90E-01	-1.79E+00	down
3-(4-Hydroxyphenyl)-1-propanol	Others	Alcohol compounds	1.04E+00	6.34E-05	6.76E-04	3.15E+00	1.66E+00	up
Methyl 4-hydroxybenzoate	Phenolic acids	Phenolic acids	1.01E+00	7.05E-05	7.18E-04	2.17E-02	-5.53E+00	down
Indole-3-butyric acid	Alkaloids	Plumerane	1.04E+00	7.14E-05	7.18E-04	1.37E+01	3.78E+00	up
Eugenyl formate	Others	Others	1.04E+00	7.15E-05	7.18E-04	6.14E-04	-1.07E+01	down
Dihydroxybenzoyl xyloside	Phenolic acids	Phenolic acids	1.04E+00	7.21E-05	7.18E-04	6.03E+00	2.59E+00	up
4'-Hydroxychalcone	Flavonoids	Chalcones	1.04E+00	7.21E-05	7.18E-04	8.62E-02	-3.54E+00	down
2,3,19,23-Tetrahydroxyolean-12-en-28-oic acid	Terpenoids	Triterpene	1.04E+00	7.24E-05	7.18E-04	7.56E+00	2.92E+00	up
Echinopsine	Alkaloids	Quinoline alkaloids	1.04E+00	7.34E-05	7.18E-04	2.32E+02	7.86E+00	up

2-Hydroxy-3-phenylpropanoic acid	Phenolic acids	Phenolic acids	1.04E+00	7.42E-05	7.18E-04	2.95E+02	8.21E+00	up
Elemicin	Others	Others	1.02E+00	7.42E-05	7.18E-04	1.44E-01	-2.79E+00	down
2',4'-Dimethylacetophenone	Others	Ketone compounds	1.04E+00	7.75E-05	7.42E-04	6.80E-02	-3.88E+00	down
3-(1H-imidazol-5-yl)-2-(trimethylammonio)propanoate	Alkaloids	Alkaloids	1.04E+00	7.92E-05	7.51E-04	5.93E+00	2.57E+00	up
Griffonilide	Others	Lactones	1.02E+00	8.33E-05	7.81E-04	9.34E-02	-3.42E+00	down
4-Aminosalicylic acid	Phenolic acids	Phenolic acids	1.04E+00	8.61E-05	7.99E-04	4.88E+01	5.61E+00	up
N-(4-oxopentyl)-acetamide	Alkaloids	Alkaloids	1.04E+00	8.82E-05	8.11E-04	9.50E+00	3.25E+00	up
Galanthamine; Galantamine; Lycoremin;	Alkaloids	Alkaloids	1.04E+00	9.05E-05	8.13E-04	7.83E+00	2.97E+00	up

Lycoremine; Jilkon								
2"-hydroxynicotinamide	Alkaloids	Alkaloids	1.04E+00	9.10E-05	8.13E-04	6.26E+00	2.65E+00	up
N-Hydroxytryptamine*	Alkaloids	Plumerane	1.04E+00	9.51E-05	8.42E-04	2.71E+02	8.08E+00	up
Dioxindole-3-acetic acid	Alkaloids	Plumerane	1.04E+00	9.86E-05	8.65E-04	5.69E+00	2.51E+00	up
Phenylethanolamine	Alkaloids	Alkaloids	1.04E+00	1.08E-04	9.36E-04	5.21E+00	2.38E+00	up
2-Methoxy-1-benzofuran-5-carbaldehyde	Others	Aldehyde compounds	1.03E+00	1.10E-04	9.47E-04	2.44E+00	1.28E+00	up
4'-Hydroxypropiophenone	Others	Ketone compounds	1.04E+00	1.13E-04	9.61E-04	6.62E-02	-3.92E+00	down
5-formyl-2,6-dihydroxy-1,7-	Terpenoids	Ditepenoids	1.03E+00	1.14E-04	9.64E-04	3.10E-01	-1.69E+00	down

dimethyl-9,10-dihydrophenanthrene								
4-Methylphenol	Others	Others	1.02E+00	1.16E-04	9.66E-04	3.23E+00	1.69E+00	up
Cornoside	Phenolic acids	Phenolic acids	1.04E+00	1.16E-04	9.66E-04	5.96E+00	2.58E+00	up
O-Phosphorylethanolamine	Alkaloids	Alkaloids	1.04E+00	1.19E-04	9.75E-04	4.60E+01	5.52E+00	up
Armexifolin	Terpenoids	Sesquiterpenoids	1.04E+00	1.24E-04	1.00E-03	7.09E-02	-3.82E+00	down
icariside D1	Others	Ketone compounds	1.04E+00	1.25E-04	1.00E-03	6.20E+00	2.63E+00	up
6-Hydroxy-2'-methoxyflavone	Flavonoids	Flavones	1.04E+00	1.25E-04	1.00E-03	5.65E-02	-4.14E+00	down
Zarzissine	Alkaloids	Alkaloids	1.04E+00	1.27E-04	1.01E-03	2.55E+01	4.67E+00	up
Nobiletin (5,6,7,8,3',4'-Hexamethoxyflavone)	Flavonoids	Flavones	1.04E+00	1.33E-04	1.05E-03	3.08E-01	-1.70E+00	down
Sophoraflavanone G	Flavonoids	Flavanones	1.04E+00	1.34E-04	1.05E-03	6.77E-02	-3.88E+00	down

Acacetin*	Flavonoids	Flavones	1.00E+00	1.37E-04	1.06E-03	5.03E-02	-4.31E+00	down
Deacetylinulicin(Britannilactone)	Terpenoids	Sesquiterpenoids	1.04E+00	1.39E-04	1.07E-03	5.14E+00	2.36E+00	up
4-Methylazetidine-2-Carboxylic acid*	Alkaloids	Alkaloids	1.04E+00	1.41E-04	1.08E-03	2.23E+01	4.48E+00	up
Tricin-7-O-(2''-feruloyl)glucuronide	Flavonoids	Flavones	1.04E+00	1.45E-04	1.10E-03	7.64E-03	-7.03E+00	down
Trigonelline	Alkaloids	Pyridine alkaloids	1.04E+00	1.46E-04	1.10E-03	6.57E+00	2.72E+00	up
Mucic acid Dimethyl Ester	Phenolic acids	Phenolic acids	1.04E+00	1.47E-04	1.10E-03	1.42E+01	3.82E+00	up
2-Hydroxy-2,3-dihydronaphthalene-1,4-dione	Quinones	Quinones	1.02E+00	1.49E-04	1.10E-03	1.67E-01	-2.58E+00	down
3-Methylsalicylic Acid	Phenolic acids	Phenolic acids	1.04E+00	1.50E-04	1.10E-03	2.67E+00	1.41E+00	up

Tyrosol; 4-Hydroxyphenylethanol	Others	Alcohol compounds	1.04E+00	1.61E-04	1.17E-03	2.41E+00	1.27E+00	up
2-Picolinic acid	Alkaloids	Pyridine alkaloids	1.04E+00	1.62E-04	1.17E-03	1.23E+02	6.94E+00	up
2,5-Dibenzoylglucaric acid	Phenolic acids	Phenolic acids	1.04E+00	1.68E-04	1.20E-03	9.26E+00	3.21E+00	up
Aurantiamide acetate	Alkaloids	Alkaloids	1.03E+00	1.73E-04	1.22E-03	7.64E-02	-3.71E+00	down
2-Methoxy-5-nitrophenol	Others	Others	1.04E+00	1.75E-04	1.22E-03	5.06E-02	-4.30E+00	down
Propyl gallate	Phenolic acids	Phenolic acids	1.04E+00	1.76E-04	1.22E-03	6.86E+00	2.78E+00	up
3-O-Hydroxybenzoyl Methylgallate	Phenolic acids	Phenolic acids	1.04E+00	1.84E-04	1.25E-03	6.26E-03	-7.32E+00	down
5-Aminolevulinic Acid	Alkaloids	Alkaloids	1.04E+00	1.84E-04	1.25E-03	1.87E+01	4.23E+00	up
2-Hydroxybenzalde	Others	Aldehyde compounds	1.04E+00	1.96E-04	1.32E-03	6.05E+01	5.92E+00	up

hyde (Salicylaldehyde)								
Embelin	Quinones	Quinones	1.04E+00	1.98E-04	1.33E-03	1.68E-01	-2.58E+00	down
3,5,6,7-Tetramethoxyflavone*	Flavonoids	Flavonols	1.04E+00	2.02E-04	1.34E-03	8.28E+00	3.05E+00	up
DL-2-Aminoadipic acid	Alkaloids	Alkaloids	1.03E+00	2.05E-04	1.35E-03	1.51E+01	3.91E+00	up
N-Caffeoylputrescine	Alkaloids	Phenolamine	1.04E+00	2.05E-04	1.35E-03	5.67E+00	2.50E+00	up
Robinlin	Terpenoids	Monoterpenoids	1.04E+00	2.09E-04	1.36E-03	1.96E-02	-5.67E+00	down
Mandelic acid	Phenolic acids	Phenolic acids	1.04E+00	2.13E-04	1.37E-03	1.61E-01	-2.64E+00	down
4,5-Epoxyartemisinic Acid	Terpenoids	Sesquiterpenoids	1.03E+00	2.14E-04	1.37E-03	3.52E-01	-1.51E+00	down
4,6-Dihydroxyquinoline	Alkaloids	Quinoline alkaloids	1.04E+00	2.16E-04	1.37E-03	1.04E-02	-6.59E+00	down

Serotonin; 5-Hydroxytryptamine*	Alkaloids	Plumerane	1.04E+00	2.17E-04	1.37E-03	2.73E+02	8.09E+00	up
Gentiolactone	Terpenoids	Monoterpenoids	1.03E+00	2.20E-04	1.38E-03	1.21E-01	-3.04E+00	down
N-(2-Hydroxy-4-methoxyphenyl)acetamide	Alkaloids	Alkaloids	1.04E+00	2.28E-04	1.43E-03	4.73E+01	5.56E+00	up
3,4-Methylenedioxycinnamaldehyde	Others	Aldehyde compounds	1.04E+00	2.32E-04	1.43E-03	2.64E-02	-5.25E+00	down
Methyl (4E,8E,11E)-5,9,13-trimethyltetradeca-4,8,11-trienoate	Others	Others	1.03E+00	2.34E-04	1.44E-03	2.48E-01	-2.01E+00	down
6-Deoxyfagomine	Alkaloids	Piperidine alkaloids	1.04E+00	2.39E-04	1.45E-03	6.65E+00	2.73E+00	up
2'-Hydroxy-4'-Methylacetophenone	Phenolic acids	Phenolic acids	1.04E+00	2.41E-04	1.46E-03	9.00E-02	-3.47E+00	down

Magnocurarine	Alkaloids	Isoquinoline alkaloids	1.04E+00	2.43E-04	1.46E-03	6.05E+00	2.60E+00	up
Lauryldiethanolamine	Others	Others	1.04E+00	2.44E-04	1.46E-03	5.09E-02	-4.30E+00	down
3,5-Dihydroxy-4-methoxybenzoic acid; 4-O-Methylgallic Acid	Phenolic acids	Phenolic acids	1.04E+00	2.52E-04	1.49E-03	5.43E+00	2.44E+00	up
L-Tyramine	Alkaloids	Alkaloids	1.04E+00	2.53E-04	1.49E-03	2.79E+02	8.12E+00	up
o-Carboxy-5-hydroxytryptamine	Alkaloids	Alkaloids	1.04E+00	2.57E-04	1.49E-03	6.24E+00	2.64E+00	up
5,6,7,7a-tetrahydro-4,4,7a-trimethyl-2(4H)-benzofuranone	Others	Others	1.04E+00	2.58E-04	1.49E-03	1.42E-02	-6.14E+00	down
4-Hydroxybenzaldehyde	Others	Aldehyde compounds	1.04E+00	2.61E-04	1.50E-03	4.28E-02	-4.55E+00	down

3-hydroxy-5-methoxybenzaldehyde	Phenolic acids	Phenolic acids	1.04E+00	2.62E-04	1.50E-03	7.81E-02	-3.68E+00	down
Lauramine oxide	Others	Others	1.04E+00	2.64E-04	1.50E-03	8.12E-02	-3.62E+00	down
Cyclen	Alkaloids	Alkaloids	1.04E+00	2.67E-04	1.51E-03	9.25E-02	-3.43E+00	down
Juglone; 5-Hydroxy-1,4-naphthoquinone	Quinones	Quinones	1.01E+00	2.73E-04	1.53E-03	2.47E-01	-2.02E+00	down
Pyrrole-2-carboxylic acid	Alkaloids	Pyrrole alkaloids	1.04E+00	2.77E-04	1.54E-03	6.84E+00	2.77E+00	up
Hypaphorine	Alkaloids	Plumerane	1.04E+00	2.81E-04	1.55E-03	1.17E-01	-3.09E+00	down
4-O-Glucosyl-2-hydroxy-6-methoxyacetophenone	Phenolic acids	Phenolic acids	1.04E+00	2.86E-04	1.55E-03	1.18E+01	3.56E+00	up
Batatasin V (2'-Hydroxy-3,4,5-trimethoxybibenzyl)	Others	Stilbene	1.04E+00	2.87E-04	1.55E-03	7.10E-02	-3.82E+00	down

Harman-3-carboxylic acid	Alkaloids	Plumerane	1.04E+00	2.89E-04	1.55E-03	4.78E-02	-4.39E+00	down
[4]-Shogaol	Phenolic acids	Phenolic acids	1.04E+00	2.89E-04	1.55E-03	4.85E-03	-7.69E+00	down
Hydroxyindesteolide	Others	Lactones	1.04E+00	2.90E-04	1.55E-03	5.32E-02	-4.23E+00	down
Senkyunolide I	Others	Lactones	1.04E+00	2.94E-04	1.56E-03	3.92E+00	1.97E+00	up
5,2'-Dihydroxy-7-methoxyflavanone	Flavonoids	Flavones	1.04E+00	2.97E-04	1.56E-03	5.64E-02	-4.15E+00	down
3-(4-methoxy-9H-pyrido[3,4-b]indol-1-yl)propanoic acid	Alkaloids	Alkaloids	1.04E+00	2.99E-04	1.56E-03	6.96E+00	2.80E+00	up
Deacetylclavaminol H	Others	Others	1.04E+00	3.04E-04	1.58E-03	3.51E-03	-8.15E+00	down
Justicidin C*	Lignans and Coumarins	Lignans	1.04E+00	3.04E-04	1.58E-03	2.00E-01	-2.32E+00	down

3-[(1-Carboxyvinyl)oxy]benzoic acid	Phenolic acids	Phenolic acids	1.04E+00	3.17E-04	1.61E-03	7.14E+00	2.83E+00	up
triptonoterpene	Terpenoids	Ditepenoids	1.04E+00	3.18E-04	1.61E-03	2.40E-02	-5.38E+00	down
Harmol	Alkaloids	Alkaloids	1.04E+00	3.18E-04	1.61E-03	5.15E+00	2.36E+00	up
Quinoline-4-carboxylic acid	Alkaloids	Quinoline alkaloids	1.04E+00	3.20E-04	1.61E-03	5.71E+01	5.84E+00	up
2,3,19,23-Tetrahydroxyurs-12-en-28-oic acid	Terpenoids	Triterpene	1.04E+00	3.22E-04	1.61E-03	5.08E+00	2.34E+00	up
Plumbagin Methyl Ether; 5-Methoxy-2-Methyl-1,4-Naphthoquinone	Quinones	Quinones	1.04E+00	3.22E-04	1.61E-03	2.90E+00	1.53E+00	up
2,3-Dihydroxyurs-12-en-28-oic acid (Corosolic acid)*	Terpenoids	Triterpene	1.04E+00	3.24E-04	1.61E-03	8.30E-02	-3.59E+00	down

Pyran[3,4-b]indole-2-ketone	Alkaloids	Plumerane	1.03E+00	3.25E-04	1.61E-03	1.57E+02	7.29E+00	up
2,6-dimethoxybenzene-1,4-diol 1-O-β-D-glucopyranoside	Phenolic acids	Phenolic acids	1.04E+00	3.28E-04	1.62E-03	6.78E+00	2.76E+00	up
γ-L-glutaminy-4-hydroxybenzene	Alkaloids	Alkaloids	1.04E+00	3.43E-04	1.68E-03	8.28E+00	3.05E+00	up
6-O-Dihydrocaffeoylglucose	Phenolic acids	Phenolic acids	1.04E+00	3.44E-04	1.68E-03	5.90E+00	2.56E+00	up
Hercynine	Alkaloids	Alkaloids	1.04E+00	3.48E-04	1.69E-03	4.03E+01	5.33E+00	up
3-hydroxy-6-(2-hydroxyethyl)-2,7-bis(hydroxymethyl)-2,5-dimethyl-3H-inden-1-one	Others	Others	1.04E+00	3.59E-04	1.72E-03	1.94E-02	-5.69E+00	down

Anabasine-glucoside	Alkaloids	Pyridine alkaloids	1.03E+00	3.59E-04	1.72E-03	5.87E+02	9.20E+00	up
4'-O-Methylbavachalcone	Flavonoids	Chalcones	1.04E+00	3.63E-04	1.73E-03	7.08E-02	-3.82E+00	down
N-(3-hydroxy-4-methoxyphenethyl)-4-hydroxybutanamide	Alkaloids	Alkaloids	1.04E+00	3.65E-04	1.73E-03	5.10E+00	2.35E+00	up
1,2-dimethylquinolin-4(1H)-one	Alkaloids	Quinoline alkaloids	1.04E+00	3.75E-04	1.76E-03	1.26E-03	-9.63E+00	down
5-(hydroxymethyl)-2(5H)-furanone	Others	Others	1.02E+00	3.78E-04	1.76E-03	5.63E+00	2.49E+00	up
Decanoyl L-Carnitine	Alkaloids	Alkaloids	1.04E+00	3.83E-04	1.77E-03	7.09E+00	2.83E+00	up
2,4-Dinitrophenol	Others	Others	1.04E+00	3.84E-04	1.77E-03	7.84E-02	-3.67E+00	down

2,4-Dihydroxybenzaldehyde	Others	Aldehyde compounds	1.03E+00	3.85E-04	1.77E-03	1.41E-01	-2.83E+00	down
Vomifoliol (Blumenol A)	Terpenoids	Monoterpenoids	1.04E+00	3.89E-04	1.77E-03	5.16E+00	2.37E+00	up
(1R,4aS,4bR,7R,8R,8aR,9S,10aR)-7-ethenyl-8,8a,9-trihydroxy-1,4a,7-trimethyl-2,3,4,4b,5,6,8,9,10,10a-decahydrophenanthrene-1-carboxylic acid	Terpenoids	Terpene	1.04E+00	3.89E-04	1.77E-03	4.57E-02	-4.45E+00	down
Rubiadin-1-Methyl Ether	Quinones	Anthraquinone	1.04E+00	3.91E-04	1.77E-03	5.64E-02	-4.15E+00	down
Valpromide	Alkaloids	Alkaloids	1.04E+00	4.08E-04	1.83E-03	1.26E-02	-6.31E+00	down
Aspidinol	Others	Others	1.04E+00	4.08E-04	1.83E-03	2.67E+01	4.74E+00	up

6'-Hydroxy-2,2',3',4',5'-Pentamethoxychalcone	Flavonoids	Chalcones	1.03E+00	4.09E-04	1.83E-03	5.30E-02	-4.24E+00	down
2,6-Dimethoxy-4-hydroxyphenol-1-O-ß-D-glucopyranoside	Others	Others	1.04E+00	4.20E-04	1.87E-03	7.94E+00	2.99E+00	up
(1R,3S)-1-Methyl-1,2,3,4-tetrahydro-β-carboline-3-carboxylic acid*	Alkaloids	Plumerane	1.04E+00	4.26E-04	1.88E-03	5.76E+00	2.53E+00	up
2-hydroxymethyl benzoic acid	Phenolic acids	Phenolic acids	1.04E+00	4.30E-04	1.88E-03	3.44E+00	1.78E+00	up
acetophenone	Others	Others	1.04E+00	4.33E-04	1.88E-03	1.03E+01	3.36E+00	up
1-hydroxy-4-methoxy-3-methyl-2,3-dihydro-1H-	Alkaloids	Alkaloids	1.03E+00	4.34E-04	1.88E-03	7.49E-02	-3.74E+00	down

indolo[3,2,1-de][1,5]naphthyridine-5,6-dione								
4-methyl-1,5,2,3-dioxadiazinan-2-amine	Others	Others	1.04E+00	4.37E-04	1.88E-03	4.13E+01	5.37E+00	up
L-Pipecolic Acid	Alkaloids	Piperidine alkaloids	1.04E+00	4.37E-04	1.88E-03	4.05E+01	5.34E+00	up
5-hydroxy-3,4-dimethyl-5-pentylfuran-2(5H)-one*	Others	Lactones	1.04E+00	4.38E-04	1.88E-03	1.97E-02	-5.66E+00	down
alanine betaine	Alkaloids	Alkaloids	1.04E+00	4.38E-04	1.88E-03	2.68E+01	4.74E+00	up
3-(4-aminobut-1-en-1-yl)-4,7-dihydro-1-indole-5,6-dione	Alkaloids	Alkaloids	1.04E+00	4.43E-04	1.89E-03	6.58E+00	2.72E+00	up
pyridoxamine	Alkaloids	Pyridine alkaloids	1.04E+00	4.48E-04	1.90E-03	6.02E+00	2.59E+00	up
Spermidine	Alkaloids	Alkaloids	1.04E+00	4.51E-04	1.90E-03	1.18E+02	6.88E+00	up

Quercetin-5-O-β-D-glucoside*	Flavonoids	Flavonols	1.04E+00	4.52E-04	1.90E-03	6.41E+00	2.68E+00	up
Dehydrovomifoliol	Terpenoids	Monoterpenoids	1.04E+00	4.59E-04	1.92E-03	7.44E+00	2.90E+00	up
Scopine	Alkaloids	Tropan alkaloids	1.04E+00	4.68E-04	1.95E-03	3.66E+00	1.87E+00	up
3,5,6,7,8,3',4'-Heptamethoxyflavone	Flavonoids	Flavonols	1.04E+00	4.74E-04	1.97E-03	9.68E+00	3.27E+00	up
tetrahydroharmol	Alkaloids	Alkaloids	1.04E+00	4.77E-04	1.97E-03	5.45E+00	2.45E+00	up
Hexadecyl ethanolamine	Alkaloids	Alkaloids	1.04E+00	4.82E-04	1.98E-03	8.00E-02	-3.64E+00	down
Prunetin (5,4'-Dihydroxy-7-methoxyisoflavone)*	Flavonoids	Isoflavones	1.04E+00	4.87E-04	2.00E-03	6.96E-02	-3.84E+00	down
O-Acetyl-L-carnitine	Alkaloids	Alkaloids	1.04E+00	4.99E-04	2.02E-03	4.01E+01	5.33E+00	up
Deoxyarbutin	Phenolic acids	Phenolic acids	1.04E+00	5.10E-04	2.05E-03	2.76E-02	-5.18E+00	down
Cajaninstilbene Acid	Phenolic acids	Phenolic acids	1.04E+00	5.11E-04	2.05E-03	8.10E-02	-3.63E+00	down

3,5,11(13)-Trieneudesma-13-oic acid	Terpenoids	Sesquiterpenoids	1.03E+00	5.15E-04	2.05E-03	2.03E-01	-2.30E+00	down
Ethylparaben	Phenolic acids	Phenolic acids	1.04E+00	5.17E-04	2.06E-03	8.12E-02	-3.62E+00	down
3',4',5',5,7-Pentamethoxyflavone*	Flavonoids	Flavones	1.04E+00	5.27E-04	2.08E-03	1.74E-01	-2.53E+00	down
[(1R,2S)-1-(1,3-benzodioxol-5-yl)-2-methyl-3-oxobutyl]4-hydroxy-3-methoxybenzoate	Lignans and Coumarins	Lignans	1.04E+00	5.34E-04	2.09E-03	1.79E-01	-2.48E+00	down
Tryptophol	Alkaloids	Plumerane	1.03E+00	5.41E-04	2.10E-03	1.73E-02	-5.85E+00	down
6,8-Dihydroxy-3-methylisocoumarin	Lignans and Coumarins	Coumarins	1.03E+00	5.45E-04	2.11E-03	3.99E-02	-4.65E+00	down
cis-Citral	Terpenoids	Monoterpenoids	1.04E+00	5.53E-04	2.13E-03	7.78E+00	2.96E+00	up

3,4-Dihydroxybenzeneacetic acid*	Phenolic acids	Phenolic acids	1.04E+00	5.56E-04	2.14E-03	2.64E+01	4.72E+00	up
Nagilactone C glucoside	Terpenoids	Ditepenoids	1.04E+00	5.79E-04	2.21E-03	1.68E-02	-5.89E+00	down
Isaindigodione	Alkaloids	Plumerane	1.04E+00	5.81E-04	2.21E-03	2.96E-03	-8.40E+00	down
3-Methyl-1-pentanol	Others	Alcohol compounds	1.04E+00	5.85E-04	2.21E-03	8.04E+00	3.01E+00	up
Pyrocatechol	Others	Others	1.04E+00	5.90E-04	2.22E-03	5.85E+00	2.55E+00	up
Levopimaric acid	Terpenoids	Ditepenoids	1.02E+00	5.94E-04	2.22E-03	2.64E-01	-1.92E+00	down
Trachelogenin	Lignans and Coumarins	Lignans	1.04E+00	6.00E-04	2.22E-03	2.72E-02	-5.20E+00	down
Tricin-4'-O-[β-guaiacyl-(9''-O-acetyl)glycerol]ether	Flavonoids	Flavones	1.04E+00	6.02E-04	2.22E-03	3.29E-02	-4.92E+00	down
2,3,4-Trihydroxybenzoic acid	Phenolic acids	Phenolic acids	1.04E+00	6.03E-04	2.22E-03	7.07E+00	2.82E+00	up

Quercetin-3-O-neohesperidoside*	Flavonoids	Flavonols	1.04E+00	6.05E-04	2.22E-03	6.89E+00	2.79E+00	up
Acetryptine	Alkaloids	Plumerane	1.04E+00	6.07E-04	2.22E-03	8.77E-04	-1.02E+01	down
2-Caffeoyl-L-tartaric acid (Caftaric acid)	Phenolic acids	Phenolic acids	1.04E+00	6.09E-04	2.22E-03	5.43E+00	2.44E+00	up
6,10,14-Trimethylpentadeca-5,9-Diene-2,13-Dione	Others	Others	1.03E+00	6.09E-04	2.22E-03	2.94E-01	-1.77E+00	down
Arsanin	Terpenoids	Sesquiterpenoids	1.04E+00	6.12E-04	2.22E-03	5.41E-02	-4.21E+00	down
Benzoyltartaric acid	Phenolic acids	Phenolic acids	1.04E+00	6.17E-04	2.23E-03	6.64E+00	2.73E+00	up
9,11-dimethoxy-2h-[1,3]dioxolo[4,5-b]xanthen-10-one*	Flavonoids	Other Flavonoids	1.04E+00	6.26E-04	2.24E-03	2.55E-02	-5.29E+00	down

7-Hydroxycoumarin-O-rhamnoside	Lignans and Coumarins	Coumarins	1.04E+00	6.28E-04	2.24E-03	1.11E+01	3.48E+00	up
6,7,8-Tetrahydroxy-5-methoxyflavone	Flavonoids	Flavones	1.04E+00	6.31E-04	2.24E-03	4.01E-02	-4.64E+00	down
1-[3-(4-methyl-6-oxocyclohex-1-en-1-yl)propyl]pyrrolidin-2-one	Others	Ketone compounds	1.04E+00	6.31E-04	2.24E-03	3.44E+01	5.10E+00	up
Apigenin-7-O-(6"-feruloyl)glucuronide	Flavonoids	Flavones	1.04E+00	6.50E-04	2.30E-03	1.05E-01	-3.25E+00	down
Cinnamic acid	Phenolic acids	Phenolic acids	1.04E+00	6.53E-04	2.30E-03	2.58E-01	-1.95E+00	down
Angustoline	Alkaloids	Plumerane	1.04E+00	6.60E-04	2.32E-03	7.55E+00	2.92E+00	up
Dibutylphthalate*	Phenolic acids	Phenolic acids	1.02E+00	6.62E-04	2.32E-03	3.18E-01	-1.65E+00	down
2-Phenyloxirane	Others	Others	1.04E+00	6.66E-04	2.32E-03	6.28E+02	9.30E+00	up

3,7,3'-Trihydroxyflavone	Flavonoids	Flavones	1.04E+00	6.77E-04	2.35E-03	5.67E+00	2.50E+00	up
Dehydrololiolide	Terpenoids	Terpene	1.04E+00	6.97E-04	2.41E-03	5.51E-04	-1.08E+01	down
Poncirin (Isosakuranetin-7-O-neohesperidoside)*	Flavonoids	Flavanones	1.02E+00	7.05E-04	2.43E-03	2.64E+00	1.40E+00	up
9-[(2S,3S,4S,5R)-3,4-dihydroxy-5-(hydroxymethyl)oxolan-2-yl]-1H-purin-6-one	Alkaloids	Alkaloids	1.04E+00	7.07E-04	2.43E-03	2.56E+02	8.00E+00	up
N',N'',N'''-p-Coumaroyl-cinnamoyl-caffeoyl spermidine	Alkaloids	Phenolamine	1.04E+00	7.17E-04	2.45E-03	5.14E+00	2.36E+00	up

3,19,23-Trihydroxyurs-12-en-28-oic acid (Rutundic acid)	Terpenoids	Triterpene	1.04E+00	7.25E-04	2.47E-03	1.05E-01	-3.25E+00	down
3,4-dimethoxy-2-[(3,4,5-trihydroxy-6-{[(3,4,5-trihydroxy-6-methyloxan-2-yl)oxy]methyl}oxan-2-yl)oxy]xanthen-9-one	Flavonoids	Other Flavonoids	1.04E+00	7.34E-04	2.49E-03	6.28E+00	2.65E+00	up
Methyl gallate	Phenolic acids	Phenolic acids	1.01E+00	7.38E-04	2.50E-03	2.20E+00	1.14E+00	up
4-(hydroxymethyl)-1,8-dimethyl-9,10-	Terpenoids	Ditepenoids	1.04E+00	7.59E-04	2.56E-03	3.49E-01	-1.52E+00	down

dihydrophenanthrene-2,7-diol								
Mussaenosidic acid	Terpenoids	Monoterpenoids	1.04E+00	7.62E-04	2.56E-03	6.44E+00	2.69E+00	up
Kurarinone	Flavonoids	Flavanones	1.04E+00	7.81E-04	2.61E-03	4.37E-02	-4.52E+00	down
4'-Hydroxy-5,7-dimethoxyflavanone	Flavonoids	Flavanones	1.04E+00	7.86E-04	2.61E-03	5.52E-02	-4.18E+00	down
Radicamine B	Alkaloids	Pyrrole alkaloids	1.04E+00	7.86E-04	2.61E-03	7.20E+00	2.85E+00	up
3',4',5,6,7-Pentamethoxyflavanone*	Flavonoids	Flavanones	1.04E+00	7.90E-04	2.61E-03	9.44E-02	-3.41E+00	down
1-Decanol*	Others	Alcohol compounds	1.04E+00	7.98E-04	2.62E-03	1.51E-01	-2.73E+00	down
3-Aminosalicylic acid	Phenolic acids	Phenolic acids	1.04E+00	8.10E-04	2.65E-03	3.12E+01	4.96E+00	up
Dihydroactinidiolide	Terpenoids	Monoterpenoids	1.04E+00	8.18E-04	2.66E-03	2.77E-02	-5.18E+00	down
Hydroxydihydrobovolide*	Others	Lactones	1.04E+00	8.20E-04	2.66E-03	1.75E-02	-5.84E+00	down

2-Pentyl-3-phenyl-2-propenal	Others	Aldehyde compounds	1.01E+00	8.24E-04	2.66E-03	2.69E-01	-1.90E+00	down
Pteleifolosin A	Others	Others	1.04E+00	8.26E-04	2.66E-03	3.33E-02	-4.91E+00	down
N-2-Phenylethyl-cinnamamide	Alkaloids	Phenolamine	1.04E+00	8.36E-04	2.68E-03	4.90E-02	-4.35E+00	down
Dendronobilin I	Terpenoids	Sesquiterpenoids	1.04E+00	8.40E-04	2.69E-03	2.12E-02	-5.56E+00	down
Libocedrine B	Terpenoids	Sesquiterpenoids	1.04E+00	8.48E-04	2.71E-03	3.52E-03	-8.15E+00	down
Linalyl Acetate	Others	Others	1.02E+00	8.52E-04	2.71E-03	1.08E+01	3.43E+00	up
4-hydroxyphenyl acrylaldehyde	Others	Aldehyde compounds	1.04E+00	8.62E-04	2.73E-03	8.24E-03	-6.92E+00	down
acerogenic acid	Terpenoids	Triterpene	1.04E+00	8.71E-04	2.75E-03	9.53E-02	-3.39E+00	down
Hesperetin-7-O-rutinoside (Hesperidin)	Flavonoids	Flavanones	1.03E+00	8.86E-04	2.78E-03	6.77E+01	6.08E+00	up
Syringic acid 4-O-rhamnoside	Phenolic acids	Phenolic acids	1.04E+00	9.04E-04	2.83E-03	5.59E+00	2.48E+00	up
Glycitein	Flavonoids	Isoflavones	1.02E+00	9.19E-04	2.86E-03	2.99E+00	1.58E+00	up

1,4-dideoxy-1,4-imino-D-arabinitol	Alkaloids	Pyrrole alkaloids	1.02E+00	9.20E-04	2.86E-03	6.71E+00	2.75E+00	up
hemerocallin	Phenolic acids	Phenolic acids	1.04E+00	9.26E-04	2.86E-03	6.32E-02	-3.98E+00	down
5-guanidino-2-(2-hydroxyethyl)pentanoic acid	Others	Others	1.04E+00	9.29E-04	2.86E-03	1.75E+01	4.13E+00	up
Octyl gallate	Phenolic acids	Phenolic acids	1.04E+00	9.29E-04	2.86E-03	1.22E-01	-3.04E+00	down
Sophoraflavanone B	Flavonoids	Flavanones	1.04E+00	9.40E-04	2.88E-03	1.56E-01	-2.68E+00	down
Methyl 2,4-dihydroxyphenylacetate*	Phenolic acids	Phenolic acids	1.04E+00	9.52E-04	2.89E-03	5.60E+00	2.48E+00	up
Isobutyryl carnitine	Alkaloids	Alkaloids	1.04E+00	9.66E-04	2.92E-03	1.60E+01	4.00E+00	up
3-amino-2-naphthoic acid*	Alkaloids	Alkaloids	1.04E+00	9.71E-04	2.92E-03	5.20E+00	2.38E+00	up
1-Hydroxycanthin-6-one*	Alkaloids	Plumerane	1.04E+00	9.76E-04	2.92E-03	2.00E-02	-5.65E+00	down

6'-Hydroxy-3,4,2',3',4',5'-Hexamethoxychalcone	Flavonoids	Chalcones	1.04E+00	9.77E-04	2.92E-03	6.41E-02	-3.96E+00	down
N-Methylpyrrolidone	Others	Others	1.04E+00	9.78E-04	2.92E-03	8.80E-03	-6.83E+00	down
Phthalic acid	Phenolic acids	Phenolic acids	1.04E+00	9.85E-04	2.93E-03	6.97E+01	6.12E+00	up
3-(((S)-1-carboxy-2-phenylethyl)amino)-2,5-dihydroxycyclopentane-1-carboxylic acid	Others	Others	1.04E+00	9.96E-04	2.96E-03	1.92E+02	7.58E+00	up
Chloranthalic acid	Terpenoids	Sesquiterpenoids	1.04E+00	1.01E-03	3.00E-03	1.83E-02	-5.77E+00	down
2,5-Dihydroxybenzoi	Phenolic acids	Phenolic acids	1.04E+00	1.03E-03	3.05E-03	5.93E+00	2.57E+00	up

c acid; Gentisic Acid*								
Cirsilineol (4',5-Dihydroxy-3',6,7-trimethoxyflavone)	Flavonoids	Flavanones	1.04E+00	1.07E-03	3.13E-03	4.16E+00	2.06E+00	up
Tetradecyldiethanolamine	Alkaloids	Alkaloids	1.04E+00	1.08E-03	3.16E-03	5.94E-02	-4.07E+00	down
5,6,7,8-Tetramethoxyflavone	Flavonoids	Flavones	1.04E+00	1.08E-03	3.16E-03	8.17E+00	3.03E+00	up
Azoxystrobin acid	Alkaloids	Alkaloids	1.04E+00	1.08E-03	3.16E-03	7.26E-02	-3.78E+00	down
2-Piperidone	Alkaloids	Piperidine alkaloids	1.04E+00	1.09E-03	3.17E-03	3.62E-03	-8.11E+00	down
phenylalanine betaine	Alkaloids	Alkaloids	1.01E+00	1.09E-03	3.17E-03	5.35E+00	2.42E+00	up
γ-L-glutaminyl-3,4-benzoquinone	Alkaloids	Alkaloids	1.04E+00	1.10E-03	3.17E-03	5.54E+00	2.47E+00	up
Benzamide	Alkaloids	Alkaloids	1.04E+00	1.10E-03	3.17E-03	6.21E+00	2.63E+00	up

Pinocembrin (Dihydrochrysin)	Flavonoids	Flavanones	1.04E+00	1.10E-03	3.17E-03	9.01E-02	-3.47E+00	down
[8]-Gingerdione	Phenolic acids	Phenolic acids	1.04E+00	1.12E-03	3.20E-03	8.09E-02	-3.63E+00	down
Aracarpene 2*	Flavonoids	Isoflavones	1.04E+00	1.13E-03	3.23E-03	2.59E-02	-5.27E+00	down
Myricetin	Flavonoids	Flavonols	1.04E+00	1.16E-03	3.30E-03	6.83E+00	2.77E+00	up
Vanillylamine	Alkaloids	Alkaloids	1.04E+00	1.17E-03	3.32E-03	1.08E+01	3.43E+00	up
3-O-Galloyl-D-glucose*	Phenolic acids	Phenolic acids	1.04E+00	1.21E-03	3.41E-03	7.86E+00	2.97E+00	up
7β-Hydroxydarutigenol	Terpenoids	Ditepenoids	1.04E+00	1.21E-03	3.42E-03	4.90E-02	-4.35E+00	down
2-Decanol*	Others	Alcohol compounds	1.04E+00	1.22E-03	3.42E-03	1.59E-01	-2.65E+00	down
2-Phenylethanol	Others	Alcohol compounds	1.04E+00	1.23E-03	3.44E-03	9.12E+00	3.19E+00	up
5-Hydroxy-1-tetralone	Others	Ketone compounds	1.04E+00	1.24E-03	3.47E-03	3.26E-02	-4.94E+00	down
4-Methoxycinnamic acid	Phenolic acids	Phenolic acids	1.04E+00	1.26E-03	3.50E-03	9.92E-02	-3.33E+00	down

4-[(3-O-Acetyl-6-deoxy-α-L-mannosyl)oxy]benzeneacetonitrile	Alkaloids	Phenolamine	1.04E+00	1.26E-03	3.51E-03	7.64E+00	2.93E+00	up
5β-Hydroxycostic acid	Terpenoids	Sesquiterpenoids	1.04E+00	1.27E-03	3.51E-03	1.61E-01	-2.63E+00	down
Urocanic acid	Alkaloids	Alkaloids	1.03E+00	1.27E-03	3.52E-03	2.75E+00	1.46E+00	up
1-phenyl-7-(4-hydroxyphenyl)-4-ene-3-heptanone	Others	Ketone compounds	1.04E+00	1.29E-03	3.52E-03	9.74E-02	-3.36E+00	down
2,3,19-Trihydroxyolean-12-en-28-oic acid (Arjunic acid)	Terpenoids	Triterpene	1.02E+00	1.29E-03	3.52E-03	2.53E-01	-1.98E+00	down
6-Methylnicotinamide	Alkaloids	Pyridine alkaloids	1.02E+00	1.29E-03	3.52E-03	7.62E+01	6.25E+00	up

3,7,11-trimethyl-2-oxa-6,10,13-triazatricyclo[7.3.1.05,13]tridecane	Alkaloids	Alkaloids	1.04E+00	1.29E-03	3.52E-03	2.05E-01	-2.28E+00	down
3-Indoleacrylic acid*	Alkaloids	Plumerane	1.04E+00	1.31E-03	3.57E-03	5.31E+00	2.41E+00	up
Lathyrol	Terpenoids	Ditepenoids	1.04E+00	1.34E-03	3.57E-03	7.18E-02	-3.80E+00	down
Gossypetin 3,7,8,3',4'-pentamethyl ether*	Flavonoids	Flavonols	1.03E+00	1.34E-03	3.57E-03	2.27E-01	-2.14E+00	down
ent-3β,16α,17,19-Tetrahydroxykaurane	Terpenoids	Ditepenoids	1.03E+00	1.34E-03	3.57E-03	2.83E-01	-1.82E+00	down
Ellagic acid	Tannins	Tannin	1.04E+00	1.34E-03	3.57E-03	6.09E+00	2.61E+00	up
Cotinine; (S)-1-Methyl-5-(3-pyridinyl)-2-pyrrolidinone	Alkaloids	Pyridine alkaloids	1.04E+00	1.34E-03	3.57E-03	1.36E+02	7.09E+00	up

4-Hydroxybenzyl Alcohol	Others	Others	1.04E+00	1.35E-03	3.58E-03	4.64E-02	-4.43E+00	down
N-Methyl-2-(2-hydroxypropyl)-6-(2-hydroxybutyl)-Δ3-piperideine	Alkaloids	Piperidine alkaloids	1.04E+00	1.36E-03	3.59E-03	4.61E-03	-7.76E+00	down
Syringic acid	Phenolic acids	Phenolic acids	1.04E+00	1.37E-03	3.61E-03	4.02E-01	-1.32E+00	down
Dihydroisopelletierine	Alkaloids	Piperidine alkaloids	1.04E+00	1.37E-03	3.61E-03	3.28E+00	1.72E+00	up
Hydroquinone	Others	Others	1.04E+00	1.39E-03	3.63E-03	5.55E+00	2.47E+00	up
4-Ethylphenol	Phenolic acids	Phenolic acids	1.04E+00	1.39E-03	3.63E-03	6.56E+00	2.71E+00	up
dihydrocoriandrin	Lignans and Coumarins	Coumarins	1.04E+00	1.39E-03	3.63E-03	1.43E-01	-2.81E+00	down
5,6,7,4'-Tetramethoxyflavone*	Flavonoids	Flavones	1.04E+00	1.39E-03	3.63E-03	7.85E+00	2.97E+00	up
2,3-Dihydroxyurs-	Terpenoids	Triterpene	1.04E+00	1.40E-03	3.64E-03	8.59E-02	-3.54E+00	down

12,18-dien-28-oic acid*								
Abscisic acid	Terpenoids	Sesquiterpenoids	1.01E+00	1.41E-03	3.65E-03	3.23E-01	-1.63E+00	down
(2s,3r)-8-hydroxy-3-(hydroxymethyl)-2,3,9-trimethyl-2h-furo[3,2-c]chromen-4-one	Others	Others	1.04E+00	1.41E-03	3.65E-03	3.27E-02	-4.94E+00	down
Caffeyl alcohol 4-O-β-D-glucopyranoside	Others	Alcohol compounds	1.04E+00	1.42E-03	3.65E-03	5.19E+00	2.38E+00	up
8-Keto-9(10)-α-patchoulene-4α-ol	Terpenoids	Sesquiterpenoids	1.03E+00	1.43E-03	3.67E-03	9.11E-02	-3.46E+00	down
3-hydroxyphenylacetic acid*	Phenolic acids	Phenolic acids	1.04E+00	1.43E-03	3.67E-03	5.22E+00	2.38E+00	up
5,7-Dihydroxy-3,6,8,3',4'-	Flavonoids	Flavones	1.03E+00	1.46E-03	3.73E-03	3.31E-01	-1.60E+00	down

Pentamethoxyflavone*								
Asparenydiol	Lignans and Coumarins	Lignans	1.04E+00	1.48E-03	3.76E-03	6.00E+00	2.58E+00	up
2,6-Dimethoxybenzaldehyde	Others	Aldehyde compounds	1.04E+00	1.48E-03	3.76E-03	2.50E+01	4.64E+00	up
5-Demethoxynobiletin; 3',4',6,7,8-Pentamethoxyflavone*	Flavonoids	Flavones	1.04E+00	1.50E-03	3.77E-03	5.04E+00	2.33E+00	up
Indigo	Alkaloids	Plumerane	1.02E+00	1.50E-03	3.77E-03	1.60E-01	-2.65E+00	down
3,4-Dihydroxybenzoic acid (Protocatechuic acid)*	Phenolic acids	Phenolic acids	1.04E+00	1.50E-03	3.77E-03	5.61E+00	2.49E+00	up

3,5,6,7,8,4'-Hexamethoxyflavone	Flavonoids	Flavonols	1.04E+00	1.51E-03	3.77E-03	3.20E-01	-1.64E+00	down
Catechin gallate*	Flavonoids	Flavanols	1.04E+00	1.51E-03	3.77E-03	6.77E+00	2.76E+00	up
Koaburaside	Others	Others	1.04E+00	1.52E-03	3.78E-03	6.75E+00	2.76E+00	up
2-Phenylethylamine	Alkaloids	Benzylphenylethylamine alkaloids	1.04E+00	1.52E-03	3.78E-03	1.22E+01	3.61E+00	up
Baldrinal	Terpenoids	Monoterpenoids	1.04E+00	1.53E-03	3.78E-03	6.44E-03	-7.28E+00	down
7-O-Methyltectorigenin; 4',5-Dihydroxy-6,7-Dimethoxyisoflavone*	Flavonoids	Isoflavones	1.04E+00	1.53E-03	3.78E-03	6.03E+00	2.59E+00	up
Citronellol	Others	Alcohol compounds	1.03E+00	1.55E-03	3.82E-03	5.79E-02	-4.11E+00	down
5,7,8,3',4'-Pentamethoxyflavanone*	Flavonoids	Flavanones	1.04E+00	1.56E-03	3.82E-03	9.84E-02	-3.34E+00	down
Benzoyleneurea	Alkaloids	Alkaloids	1.02E+00	1.57E-03	3.84E-03	3.16E-02	-4.98E+00	down

4-Hydroxybenzylamine	Alkaloids	Phenolamine	1.04E+00	1.60E-03	3.89E-03	6.04E+00	2.59E+00	up
Resveratrol	Others	Stilbene	1.04E+00	1.60E-03	3.89E-03	5.33E+00	2.41E+00	up
1,3-dihydroxy-2,3,3a,4-tetrahydropyrrolo[2,1-b]quinazolin-9(1H)-one	Alkaloids	Alkaloids	1.04E+00	1.61E-03	3.89E-03	3.09E+01	4.95E+00	up
Homovanillic alcohol; 4-Hydroxy-3-methoxyphenethanol	Others	Alcohol compounds	1.02E+00	1.61E-03	3.89E-03	3.14E-01	-1.67E+00	down
2-Ethyl-2,6,6-trimethylpiperidin-4-one	Alkaloids	Piperidine alkaloids	1.04E+00	1.61E-03	3.89E-03	4.15E+00	2.05E+00	up

3-quinolinecarboxylic acid	Alkaloids	Quinoline alkaloids	1.04E+00	1.64E-03	3.93E-03	3.58E-02	-4.80E+00	down
3,4-Dihydroverbenalin	Terpenoids	Monoterpenoids	1.04E+00	1.65E-03	3.93E-03	7.03E-03	-7.15E+00	down
Naringenin-7-O-Rutinoside(Narirutin)*	Flavonoids	Flavanones	1.04E+00	1.66E-03	3.94E-03	5.33E+00	2.41E+00	up
3',4'-Dihydroxy-5,6,7,8,5'-Pentamethoxyflavone*	Flavonoids	Flavones	1.03E+00	1.67E-03	3.96E-03	2.76E-01	-1.86E+00	down
8-Hydroxy-2-methoxy-1,4-naphthoquinone; 3-Methoxyjuglone	Quinones	Quinones	1.04E+00	1.68E-03	3.99E-03	5.70E+00	2.51E+00	up
Indole-3-carboxylic acid*	Alkaloids	Plumerane	1.04E+00	1.70E-03	4.00E-03	1.47E-01	-2.76E+00	down

Epigoitrin	Others	Others	1.04E+00	1.71E-03	4.00E-03	1.62E+01	4.02E+00	up
vasicinone	Alkaloids	Alkaloids	1.04E+00	1.71E-03	4.00E-03	5.84E+00	2.55E+00	up
Esculetin (6,7-Dihydroxycoumarin)	Lignans and Coumarins	Coumarins	1.04E+00	1.71E-03	4.00E-03	5.40E+00	2.43E+00	up
Epirosmanol	Terpenoids	Ditepenoids	1.04E+00	1.71E-03	4.00E-03	6.60E-02	-3.92E+00	down
Piperonylic acid	Phenolic acids	Phenolic acids	1.04E+00	1.72E-03	4.01E-03	1.90E-02	-5.72E+00	down
Anthranilic Acid	Phenolic acids	Phenolic acids	1.04E+00	1.73E-03	4.01E-03	2.21E-01	-2.18E+00	down
Quininic acid; 6-Methoxyquinoline-4-Carboxylic Acid	Alkaloids	Quinoline alkaloids	1.04E+00	1.79E-03	4.12E-03	7.68E+00	2.94E+00	up
Diosmetin (5,7,3'-Trihydroxy-4'-methoxyflavone)*	Flavonoids	Flavones	1.04E+00	1.80E-03	4.14E-03	2.01E-02	-5.64E+00	down
A-hydroxyquinoline	Alkaloids	Alkaloids	1.03E+00	1.81E-03	4.16E-03	4.11E+01	5.36E+00	up
Gossypol	Terpenoids	Sesquiterpenoids	1.04E+00	1.84E-03	4.19E-03	8.31E-02	-3.59E+00	down

2-methyl-2-vinyl-3-isopropenyl-5-isopropylidene cyclohexanol*	Terpenoids	Monoterpenoids	1.02E+00	1.84E-03	4.19E-03	2.03E+00	1.02E+00	up
7-Methoxy-3-[1-(3-pyridyl)methylidene]-4-chromanone	Flavonoids	Other Flavonoids	1.04E+00	1.85E-03	4.19E-03	5.37E+00	2.42E+00	up
3-(4-hydroxycyclohex-1-en-1-yl)-4a,5-dimethyl-3,4,4a,5,6,8a-hexahydronaphthalen-1(2H)-one	Others	Others	1.04E+00	1.85E-03	4.19E-03	1.58E-01	-2.66E+00	down
Dihydroxy-dimethoxyflavone*	Flavonoids	Flavonols	1.03E+00	1.87E-03	4.23E-03	5.68E+00	2.51E+00	up

Dihydroxyacetone phosphate	Others	Ketone compounds	1.04E+00	1.90E-03	4.28E-03	5.20E+00	2.38E+00	up
Coumestrol	Lignans and Coumarins	Coumarins	1.04E+00	1.90E-03	4.28E-03	3.77E-02	-4.73E+00	down
Lumichrome	Alkaloids	Alkaloids	1.04E+00	1.93E-03	4.32E-03	6.20E-03	-7.33E+00	down
rosacorenol	Terpenoids	Sesquiterpenoids	1.04E+00	1.94E-03	4.33E-03	1.20E-01	-3.06E+00	down
1-Methyl-6-Oxo-1,6-Dihydropyridine-3-Carboxamide	Alkaloids	Pyridine alkaloids	1.04E+00	1.95E-03	4.35E-03	5.39E+00	2.43E+00	up
N-Hexanoyl Tryptamine	Alkaloids	Plumerane	1.04E+00	1.97E-03	4.39E-03	9.00E-02	-3.47E+00	down
2-Undecanone	Others	Ketone compounds	1.04E+00	1.98E-03	4.40E-03	1.29E-01	-2.95E+00	down
D-Threo-guaiacylglycerol-7-O-β-D-glucoside	Others	Alcohol compounds	1.04E+00	1.99E-03	4.40E-03	6.66E+00	2.73E+00	up
Quercetin-3-O-robinobioside	Flavonoids	Flavonols	1.03E+00	2.00E-03	4.40E-03	7.10E+00	2.83E+00	up

N-Formyl nornicotine	Alkaloids	Pyridine alkaloids	1.04E+00	2.00E-03	4.40E-03	1.64E+02	7.36E+00	up
2-O-Feruloylhydroxycitric Acid	Phenolic acids	Phenolic acids	1.04E+00	2.02E-03	4.44E-03	6.70E+00	2.74E+00	up
3-Galloylshikimic acid	Phenolic acids	Phenolic acids	1.04E+00	2.03E-03	4.45E-03	5.33E+00	2.41E+00	up
3,3',4-O-Trimethylellagic acid	Tannins	Tannin	1.04E+00	2.05E-03	4.46E-03	4.42E-02	-4.50E+00	down
Mucic acid-1,4-lactone-2-O-gallate	Phenolic acids	Phenolic acids	1.01E+00	2.05E-03	4.46E-03	3.42E+00	1.77E+00	up
2,3-Dihydroxybenzoic Acid*	Phenolic acids	Phenolic acids	1.04E+00	2.05E-03	4.46E-03	5.58E+00	2.48E+00	up
Pantetheine	Alkaloids	Alkaloids	1.04E+00	2.08E-03	4.50E-03	5.44E-02	-4.20E+00	down

3,5,6,7,3',4'-Hexamethoxyflavone	Flavonoids	Flavonols	1.03E+00	2.09E-03	4.50E-03	3.63E+00	1.86E+00	up
4-[2-formyl-5-(hydroxymethyl) pyrrol-1-yl]butanoic acid	Alkaloids	Alkaloids	1.04E+00	2.10E-03	4.51E-03	5.93E+00	2.57E+00	up
6,7-Dihydroxy-4-methylcoumarin	Lignans and Coumarins	Coumarins	1.04E+00	2.10E-03	4.51E-03	2.71E-03	-8.53E+00	down
2-Hexylphosphoric Acid	Others	Others	1.04E+00	2.11E-03	4.51E-03	1.34E-04	-1.29E+01	down
3ξ-(1ξ-hydroxyethyl)-7-hydroxy-1-isobenzofuranone	Others	Others	1.04E+00	2.11E-03	4.51E-03	1.16E+01	3.53E+00	up
9α-Hydroxy-8β-methacryloyloxy-14-oxo-	Terpenoids	Sesquiterpenoids	1.04E+00	2.15E-03	4.57E-03	1.20E-02	-6.38E+00	down

acanthospermolide								
Justicidin A*	Lignans and Coumarins	Lignans	1.04E+00	2.15E-03	4.57E-03	2.13E-01	-2.23E+00	down
Salicylamide	Alkaloids	Alkaloids	1.04E+00	2.17E-03	4.58E-03	3.41E-01	-1.55E+00	down
polygodial	Terpenoids	Sesquiterpenoids	1.02E+00	2.17E-03	4.58E-03	1.09E-02	-6.52E+00	down
2-Geranyl-5-Hydroxy-3-N-Pentyl-1,4-Benzoquinone	Terpenoids	Sesquiterpenoids	1.04E+00	2.19E-03	4.61E-03	1.10E-01	-3.19E+00	down
10-Hydroxycanthin-6-One*	Alkaloids	Plumerane	1.04E+00	2.20E-03	4.61E-03	1.75E-02	-5.83E+00	down
Luteolin (5,7,3',4'-Tetrahydroxyflavone)	Flavonoids	Flavones	1.02E+00	2.20E-03	4.61E-03	8.37E+01	6.39E+00	up
1-(Hydroxymethyl)	Alkaloids	Pyrrole alkaloids	1.04E+00	2.21E-03	4.61E-03	2.42E+01	4.60E+00	up

hexahydro-1h-pyrrolizin-2-ol								
Pyrroline	Alkaloids	Pyrrole alkaloids	1.04E+00	2.24E-03	4.66E-03	7.70E+00	2.94E+00	up
1-NAPHTHALENESULFONIC ACID	Others	Others	1.04E+00	2.24E-03	4.66E-03	6.60E+00	2.72E+00	up
2-Aminophenol	Alkaloids	Alkaloids	1.04E+00	2.28E-03	4.72E-03	7.10E+00	2.83E+00	up
1,2,3-Trihydroxy-19-oxo-18,19-seco-urs-11,13(18)-dien-28-oic acid	Terpenoids	Triterpene	1.04E+00	2.31E-03	4.78E-03	1.42E+01	3.83E+00	up
11-Hydroxycanthin-6-One; Amalorin*	Alkaloids	Plumerane	1.04E+00	2.32E-03	4.78E-03	1.41E-02	-6.15E+00	down
Harpagoside	Terpenoids	Monoterpenoids	1.04E+00	2.34E-03	4.83E-03	9.83E-02	-3.35E+00	down

7-methoxy-9H-β-carboline-1-carboxylic acid	Alkaloids	Alkaloids	1.04E+00	2.39E-03	4.91E-03	1.71E-02	-5.87E+00	down
2-Cyanomethylphenol	Alkaloids	Alkaloids	1.04E+00	2.41E-03	4.92E-03	7.05E+00	2.82E+00	up
5,6,6a,7-tetrahydro-1,2,9,10-tetraethoxy-6-n-pentyl-4H-dibenzo[de,g]quinolin-4-ol	Alkaloids	Alkaloids	1.04E+00	2.43E-03	4.96E-03	5.52E+00	2.46E+00	up
9β-hydroxyeudesma-4,11(13)-dien-12-al	Terpenoids	Sesquiterpenoids	1.04E+00	2.44E-03	4.97E-03	9.62E-02	-3.38E+00	down
3,7-Dihydroxyolean-	Terpenoids	Triterpene	1.01E+00	2.45E-03	4.97E-03	8.02E-02	-3.64E+00	down

12-en-28-oic acid (Rubusic acid)								
2,3-Dihydroxy-12-ursen-28-oic acid*	Terpenoids	Triterpene	1.04E+00	2.47E-03	5.00E-03	7.99E-02	-3.65E+00	down
2-Amino-1,3-eicosanediol	Others	Alcohol compounds	1.04E+00	2.48E-03	5.00E-03	7.35E-02	-3.77E+00	down
Oxyphyllone C	Terpenoids	Sesquiterpenoids	1.04E+00	2.53E-03	5.08E-03	7.26E+00	2.86E+00	up
N-p-Coumaroylhydroxyagmatine	Alkaloids	Phenolamine	1.04E+00	2.53E-03	5.08E-03	6.17E+00	2.62E+00	up
Indole-3-lactic acid	Alkaloids	Plumerane	1.02E+00	2.57E-03	5.15E-03	2.42E+01	4.60E+00	up
Catechin	Flavonoids	Flavanols	1.04E+00	2.59E-03	5.17E-03	4.88E-02	-4.36E+00	down
2-oxindole-3-acetic acid	Alkaloids	Plumerane	1.04E+00	2.62E-03	5.21E-03	5.43E+00	2.44E+00	up
L-Azetidine-2-carboxylic acid*	Alkaloids	Alkaloids	1.01E+00	2.64E-03	5.23E-03	1.49E+01	3.90E+00	up
3'-Glucosyl-6,7-dihydroxy-N-	Alkaloids	Isoquinoline alkaloids	1.04E+00	2.64E-03	5.23E-03	5.63E+00	2.49E+00	up

methyl- benzyltetrahydroi soquinoline								
Tryptamine	Alkaloids	Plumerane	1.04E+00	2.69E-03	5.32E-03	9.15E+00	3.19E+00	up
Nordihydrocapsai cin*	Alkaloids	Phenolamine	1.01E+00	2.70E-03	5.32E-03	4.05E-01	-1.31E+00	down
Gardenin C	Flavonoids	Flavones	1.02E+00	2.72E-03	5.34E-03	2.53E-01	-1.98E+00	down
Pilosanone A	Terpenoids	Monoterpenoids	1.04E+00	2.72E-03	5.34E-03	1.15E-01	-3.12E+00	down
5,7-dihydroxy-2-(3-hydroxyphenyl)-3-methoxy-4H-chromen-4-one*	Flavonoids	Flavonols	1.04E+00	2.73E-03	5.35E-03	1.66E-02	-5.91E+00	down
3,3'-O-Dimethylellagic Acid	Tannins	Tannin	1.04E+00	2.74E-03	5.35E-03	8.50E-02	-3.56E+00	down
USaramine	Alkaloids	Alkaloids	1.04E+00	2.74E-03	5.35E-03	5.29E+00	2.40E+00	up
8-(methoxymethox	Others	Others	1.04E+00	2.81E-03	5.47E-03	5.77E+00	2.53E+00	up

y)decyl hydrogen sulfate								
Vasicine	Alkaloids	Pyrrole alkaloids	1.04E+00	2.84E-03	5.49E-03	5.84E+00	2.55E+00	up
3-Chloroaniline	Alkaloids	Alkaloids	1.04E+00	2.84E-03	5.49E-03	3.11E-01	-1.68E+00	down
Quercetin-3-O-glucoside-7-O-rhamnoside*	Flavonoids	Flavonols	1.04E+00	2.85E-03	5.49E-03	8.37E+00	3.07E+00	up
6-O-Galloyl-β-D-glucose*	Phenolic acids	Phenolic acids	1.04E+00	2.85E-03	5.49E-03	6.98E+00	2.80E+00	up
naphthisoxazol A*	Alkaloids	Alkaloids	1.04E+00	2.86E-03	5.50E-03	5.39E+00	2.43E+00	up
1,7-Diphenyl-2-hydroxy-heptene	Others	Others	1.04E+00	2.90E-03	5.55E-03	6.43E-02	-3.96E+00	down
3,23-Dihydroxyolean-12-en-28-oic acid (Hederagenin)*	Terpenoids	Triterpene	1.04E+00	2.91E-03	5.56E-03	9.75E-02	-3.36E+00	down
3,4-methylenedioxy cinnamyl alcohol	Others	Alcohol compounds	1.03E+00	2.91E-03	5.56E-03	2.00E-02	-5.64E+00	down

5-Hydroxy-7,3',4'-trimethoxyflavone*	Flavonoids	Flavones	1.04E+00	2.93E-03	5.59E-03	1.22E+01	3.61E+00	up
14-Hydroxyvibsanin F	Terpenoids	Ditepenoids	1.04E+00	2.96E-03	5.64E-03	3.93E-02	-4.67E+00	down
4-Hydroxycoumarin	Lignans and Coumarins	Coumarins	1.04E+00	2.98E-03	5.66E-03	1.65E-01	-2.60E+00	down
Serratriol(Serrat-14-en-3β,21α,24-triol)	Terpenoids	Triterpene	1.04E+00	2.99E-03	5.66E-03	2.04E-02	-5.62E+00	down
Ethyl 3-Indoleacetate	Alkaloids	Plumerane	1.04E+00	3.01E-03	5.68E-03	7.01E-02	-3.83E+00	down
5-Acetylsalicylic acid	Phenolic acids	Phenolic acids	1.04E+00	3.10E-03	5.85E-03	1.66E-02	-5.91E+00	down
3,24-Dihydroxy-17,21-semiacetal-	Terpenoids	Triterpene	1.04E+00	3.18E-03	5.96E-03	1.03E-01	-3.28E+00	down

12(13)oleanolic fruit*								
Sinapinaldehyde	Others	Aldehyde compounds	1.03E+00	3.19E-03	5.97E-03	1.27E-02	-6.30E+00	down
2-Hydroxy-4-methyl-3-tridecanoyloxypentanoic acid methyl ester	Others	Others	1.04E+00	3.19E-03	5.97E-03	9.64E-02	-3.37E+00	down
Dihydrocharcone-4'-O-glucoside*	Flavonoids	Chalcones	1.04E+00	3.23E-03	6.02E-03	5.20E-03	-7.59E+00	down
3-hydroxybenzaldehyde	Others	Aldehyde compounds	1.04E+00	3.29E-03	6.08E-03	7.00E-02	-3.84E+00	down
12-hydroxy-2,6,10-trimethyltrideca-2,5-dien-4-one	Others	Others	1.04E+00	3.32E-03	6.13E-03	6.23E-02	-4.01E+00	down
2-[(9R)-1,2,3,9-tetrahydropyrrolo	Alkaloids	Alkaloids	1.04E+00	3.48E-03	6.40E-03	8.06E+01	6.33E+00	up

[2,1-b]quinazolin-9-yl]acetic acid								
1-Indanone	Others	Ketone compounds	1.04E+00	3.49E-03	6.40E-03	8.97E-02	-3.48E+00	down
Vnilloyltartaric acid	Phenolic acids	Phenolic acids	1.04E+00	3.50E-03	6.40E-03	5.61E+00	2.49E+00	up
2-Hydroxy-3-(4-Hydroxyphenyl)Propanoic Acid*	Phenolic acids	Phenolic acids	1.04E+00	3.54E-03	6.46E-03	5.83E+00	2.54E+00	up
Santamarin	Terpenoids	Sesquiterpenoids	1.04E+00	3.56E-03	6.48E-03	5.30E-02	-4.24E+00	down
Xanthohumol	Flavonoids	Chalcones	1.04E+00	3.57E-03	6.48E-03	5.76E-02	-4.12E+00	down
Ambroxide	Terpenoids	Ditepenoids	1.04E+00	3.57E-03	6.48E-03	1.36E-01	-2.88E+00	down
Septemlobin D	Terpenoids	Sesquiterpenoids	1.04E+00	3.59E-03	6.48E-03	1.31E-01	-2.93E+00	down
2-(2-Benzothiazolylthio)ethanol	Alkaloids	Alkaloids	1.02E+00	3.65E-03	6.55E-03	1.90E-01	-2.40E+00	down
Demethyl coniferin	Others	Alcohol compounds	1.04E+00	3.66E-03	6.57E-03	5.50E+00	2.46E+00	up

Dihydrocostunolide	Terpenoids	Sesquiterpenoids	1.04E+00	3.67E-03	6.57E-03	9.22E-02	-3.44E+00	down
3-Indoleacetonitrile	Alkaloids	Plumerane	1.04E+00	3.72E-03	6.64E-03	6.50E+00	2.70E+00	up
Methoxyindoleacetic acid	Alkaloids	Plumerane	1.04E+00	3.74E-03	6.66E-03	5.40E+00	2.43E+00	up
5-Demethylnobiletin; 5-Hydroxy-6,7,8,3',4'-Pentamethoxyflavone*	Flavonoids	Flavones	1.03E+00	3.82E-03	6.78E-03	2.32E-01	-2.11E+00	down
3'-Hydroxy-4'-O-methylglabridin	Flavonoids	Other Flavonoids	1.04E+00	3.85E-03	6.82E-03	9.34E-02	-3.42E+00	down
Ent-16α,17-Dihydroxykauran-2-one	Terpenoids	Ditepenoids	1.04E+00	3.85E-03	6.82E-03	3.74E-02	-4.74E+00	down
Sinapoylglucuronic acid	Phenolic acids	Phenolic acids	1.04E+00	3.86E-03	6.82E-03	7.70E+00	2.94E+00	up
Methyl cumalate	Others	Lactones	1.04E+00	3.87E-03	6.82E-03	5.59E+00	2.48E+00	up

3',4',7-Trihydroxyflavone	Flavonoids	Flavones	1.03E+00	3.89E-03	6.84E-03	4.35E-01	-1.20E+00	down
p-Methoxycinnamic acid	Phenolic acids	Phenolic acids	1.02E+00	3.90E-03	6.84E-03	6.22E-02	-4.01E+00	down
Ferulic acid*	Phenolic acids	Phenolic acids	1.04E+00	3.96E-03	6.93E-03	5.52E+00	2.46E+00	up
p-Coumaric acid	Phenolic acids	Phenolic acids	1.04E+00	3.97E-03	6.94E-03	8.50E-03	-6.88E+00	down
Desmethylxanthohumol	Flavonoids	Chalcones	1.04E+00	4.01E-03	6.97E-03	1.08E-01	-3.22E+00	down
3-Hydroxy-3,7,11-trimethyldodeca-1,6E,10-trien-9-yl isobutyrate	Terpenoids	Sesquiterpenoids	1.04E+00	4.01E-03	6.97E-03	2.36E-02	-5.40E+00	down
2-Hydroxy-scillascillin	Flavonoids	Other Flavonoids	1.04E+00	4.02E-03	6.97E-03	5.87E-02	-4.09E+00	down
2-(4-methylpent-3-enyl)-6-	Terpenoids	Ditepenoids	1.04E+00	4.05E-03	7.01E-03	1.06E-01	-3.24E+00	down

oxohept-2-enoic acid								
Pyrogallol	Others	Others	1.04E+00	4.10E-03	7.09E-03	5.91E+00	2.56E+00	up
Spermine	Alkaloids	Alkaloids	1.04E+00	4.13E-03	7.12E-03	5.48E+00	2.45E+00	up
1,4,8-Trihydroxynaphthalene-1-O-glucoside	Quinones	Naphthol	1.04E+00	4.16E-03	7.17E-03	6.10E+00	2.61E+00	up
3-Hydroxy-3',4',5,5',7-Pentamethoxyflavone*	Flavonoids	Flavones	1.00E+00	4.18E-03	7.17E-03	3.46E-01	-1.53E+00	down
3-Hydroxypyridine	Alkaloids	Pyridine alkaloids	1.04E+00	4.22E-03	7.22E-03	4.03E+00	2.01E+00	up
7-Hydroxy-4-chromone	Others	Chromone	1.04E+00	4.32E-03	7.38E-03	2.48E-03	-8.65E+00	down
4-Hydroxyphenyllactic Acid*	Phenolic acids	Phenolic acids	1.04E+00	4.34E-03	7.41E-03	5.79E+00	2.53E+00	up

5,4'-Dihydroxy-3,7-dimethoxyflavone(Kumatakenin)	Flavonoids	Flavonols	1.04E+00	4.41E-03	7.50E-03	7.75E-02	-3.69E+00	down
8-hydroxyquinoline	Alkaloids	Quinoline alkaloids	1.04E+00	4.44E-03	7.53E-03	7.28E+00	2.86E+00	up
3,5,4'-Trihydroxy-7-methoxyflavone (Rhamnocitrin)*	Flavonoids	Flavonols	1.04E+00	4.44E-03	7.53E-03	4.19E-02	-4.58E+00	down
Esculetin-7-O-glucoside	Lignans and Coumarins	Coumarins	1.04E+00	4.45E-03	7.54E-03	1.16E-01	-3.11E+00	down
4-Hydroxyphenylacetic acid*	Phenolic acids	Phenolic acids	1.03E+00	4.50E-03	7.60E-03	5.30E+00	2.41E+00	up
Diacetoxy-[6]-gingerdiol	Others	Alcohol compounds	1.01E+00	4.57E-03	7.69E-03	2.35E-01	-2.09E+00	down
Phloretin-4'-O-glucoside (Trilobatin)*	Flavonoids	Chalcones	1.04E+00	4.60E-03	7.73E-03	4.77E-03	-7.71E+00	down

5-O-Galloyl-D-hamamelose*	Phenolic acids	Phenolic acids	1.04E+00	4.64E-03	7.78E-03	7.26E+00	2.86E+00	up
Calystegine A3	Alkaloids	Tropan alkaloids	1.04E+00	4.69E-03	7.84E-03	5.51E+00	2.46E+00	up
Harmalol; Harmidol; Harmolol;	Alkaloids	Alkaloids	1.04E+00	4.71E-03	7.86E-03	6.75E+00	2.75E+00	up
1-O-Caffeoyl-β-D-glucose*	Phenolic acids	Phenolic acids	1.04E+00	4.72E-03	7.86E-03	5.78E+00	2.53E+00	up
Indole-3-acetic acid (IAA)	Alkaloids	Plumerane	1.04E+00	4.77E-03	7.93E-03	1.43E-01	-2.80E+00	down
3,23-Dihydroxy-30-noroleana-12,20(29)-dien-28-oic acid (30-Norhederagenin)	Terpenoids	Triterpene	1.04E+00	4.80E-03	7.97E-03	6.99E-02	-3.84E+00	down
1,6,8-trihydroxy-2,3,4,7-tetramethoxyxanthen-9-one	Flavonoids	Other Flavonoids	1.04E+00	4.89E-03	8.10E-03	6.58E-02	-3.92E+00	down
Cnidilide	Others	Lactones	1.04E+00	4.95E-03	8.18E-03	1.13E-01	-3.15E+00	down

Quercetin-3-O-rutinoside (Rutin)*	Flavonoids	Flavonols	1.04E+00	4.95E-03	8.18E-03	7.47E+00	2.90E+00	up
Phenoxyacetic acid*	Phenolic acids	Phenolic acids	1.03E+00	4.99E-03	8.23E-03	3.07E-01	-1.71E+00	down
Rumexobtusifolius	Others	Others	1.04E+00	5.00E-03	8.23E-03	6.10E+00	2.61E+00	up
Methyl 8,9-epoxypimaran-18-oate	Terpenoids	Terpene	1.04E+00	5.10E-03	8.38E-03	1.06E-01	-3.24E+00	down
Cyrtominetin	Flavonoids	Flavones	1.04E+00	5.16E-03	8.46E-03	5.87E+00	2.55E+00	up
Mandelamide	Alkaloids	Alkaloids	1.04E+00	5.25E-03	8.59E-03	6.66E-03	-7.23E+00	down
3,5-Dihydroxy-7,4'-dimethoxyflavone	Flavonoids	Flavonols	1.04E+00	5.31E-03	8.68E-03	7.26E-02	-3.78E+00	down
3'-O-Beta-D-xylopyranosyl plumbagic acid	Phenolic acids	Phenolic acids	1.04E+00	5.34E-03	8.70E-03	7.72E+00	2.95E+00	up

Anthraquinone-2-carboxylic acid	Quinones	Anthraquinone	1.04E+00	5.34E-03	8.70E-03	3.89E-01	-1.36E+00	down
Methyl (2E,4E,9E)-5,9,13-trimethyltetradeca-2,4,9,12-tetraenoate	Others	Others	1.04E+00	5.40E-03	8.78E-03	1.68E-01	-2.57E+00	down
3,6'-Dihydroxy-4,2',3',4',5'-Pentamethoxychalcone	Flavonoids	Chalcones	1.04E+00	5.42E-03	8.79E-03	4.91E-02	-4.35E+00	down
7βH-guaia-1(10)-en-12,8β-olide	Terpenoids	Sesquiterpenoids	1.04E+00	5.50E-03	8.90E-03	8.19E-02	-3.61E+00	down
5-Hydroxy-6,7,3',4',5'-Pentamethoxyflavone	Flavonoids	Flavones	1.04E+00	5.51E-03	8.90E-03	9.29E+00	3.22E+00	up
Indole-3-carboxaldehyde	Alkaloids	Plumerane	1.04E+00	5.55E-03	8.94E-03	1.87E-02	-5.74E+00	down

1,3-Dihydroxypropan-2-yl 12-methyl-6-(6-methylheptyl)tridec-12-enoate	Others	Others	1.04E+00	5.89E-03	9.46E-03	1.40E-01	-2.83E+00	down
3-(3,4,5-Trimethoxyphenyl)propan-1-ol	Others	Alcohol compounds	1.04E+00	5.97E-03	9.54E-03	3.39E-02	-4.88E+00	down
Flavokawain A	Flavonoids	Chalcones	1.04E+00	5.97E-03	9.54E-03	1.29E-01	-2.95E+00	down
3-Oxolup-20(29)-en-28-oic acid (Betulonic acid)*	Terpenoids	Triterpene	1.04E+00	6.03E-03	9.62E-03	5.65E-02	-4.15E+00	down
1-[2-(Furan-2-yl)-2-oxoethyl]piperidin-2-one	Alkaloids	Alkaloids	1.04E+00	6.09E-03	9.70E-03	5.66E+00	2.50E+00	up
10α-Hydroxycadin-4-en-15-al	Terpenoids	Sesquiterpenoids	1.04E+00	6.14E-03	9.76E-03	1.25E-01	-3.00E+00	down

3,28-Dihydroxylup-20(29)-ene (Betulin)	Terpenoids	Triterpene	1.04E+00	6.19E-03	9.83E-03	1.14E-01	-3.14E+00	down
Stemanthrene C*	Others	Others	1.00E+00	6.25E-03	9.89E-03	2.12E-01	-2.24E+00	down
Daidzein	Flavonoids	Isoflavones	1.04E+00	6.29E-03	9.93E-03	7.63E+00	2.93E+00	up
salaspermic acid	Terpenoids	Triterpene	1.03E+00	6.39E-03	1.01E-02	7.02E-02	-3.83E+00	down
8-Methoxyapigenin*	Flavonoids	Flavones	1.04E+00	6.41E-03	1.01E-02	2.55E-02	-5.29E+00	down
Ilicic acid	Terpenoids	Sesquiterpenoids	1.04E+00	6.43E-03	1.01E-02	8.28E-02	-3.59E+00	down
3-Galloylquinic acid	Phenolic acids	Phenolic acids	1.04E+00	6.48E-03	1.01E-02	6.82E+00	2.77E+00	up
4'-Methoxyacetophenone	Others	Ketone compounds	1.02E+00	6.50E-03	1.02E-02	2.59E-01	-1.95E+00	down
Quinacyl syringic acid	Phenolic acids	Phenolic acids	1.04E+00	6.53E-03	1.02E-02	6.75E+00	2.75E+00	up

Phthalic acid Mono-2-ethylhexyl Ester	Phenolic acids	Phenolic acids	1.04E+00	6.58E-03	1.02E-02	9.90E+00	3.31E+00	up
3-Hydroxy-24-methylene-9,19-cyclolanostan-26-oic acid (Ambolic acid)	Terpenoids	Triterpene	1.02E+00	6.59E-03	1.02E-02	4.29E-01	-1.22E+00	down
Aspernigrin A	Alkaloids	Pyridine alkaloids	1.01E+00	6.72E-03	1.04E-02	4.36E+00	2.12E+00	up
2-Methoxy-6-undecyl-1,4-benzoquinone	Quinones	Quinones	1.03E+00	6.74E-03	1.04E-02	3.42E-02	-4.87E+00	down
6,8,12-trihydroxy-2,2-dimethyl-7-(3-methylbut-2-en-1-yl)-1,10-dioxatetraphen-5-one	Others	Ketone compounds	1.03E+00	6.75E-03	1.04E-02	2.16E-01	-2.21E+00	down

2-Benzoxazolinone	Alkaloids	Alkaloids	1.04E+00	6.77E-03	1.04E-02	2.53E-02	-5.30E+00	down
8α-hydroxyeudesma-3,11(13)-dien-14-al	Terpenoids	Sesquiterpenoids	1.04E+00	6.82E-03	1.05E-02	9.79E-02	-3.35E+00	down
Benzoic acid	Phenolic acids	Phenolic acids	1.04E+00	6.94E-03	1.06E-02	2.30E-01	-2.12E+00	down
Propyl 4-hydroxybenzoate	Phenolic acids	Phenolic acids	1.04E+00	6.99E-03	1.07E-02	3.96E-01	-1.34E+00	down
Methyl[8]-gingerol	Phenolic acids	Phenolic acids	1.03E+00	7.06E-03	1.08E-02	3.48E-01	-1.52E+00	down
Epipinoresinol*	Lignans and Coumarins	Lignans	1.04E+00	7.17E-03	1.09E-02	6.46E+00	2.69E+00	up
β-Carboline-1-propanoic acid	Alkaloids	Plumerane	1.04E+00	7.18E-03	1.09E-02	1.12E+01	3.48E+00	up
6-Hydroxy-5,7,4'-trimethoxyflavone	Flavonoids	Flavones	1.03E+00	7.19E-03	1.09E-02	5.76E+00	2.53E+00	up

2-O-Galloylmucic acid	Phenolic acids	Phenolic acids	1.04E+00	7.21E-03	1.09E-02	7.29E+00	2.87E+00	up
2,3-Dihydroxylup-20(29)-en-28-oic acid (Alphitolic acid)*	Terpenoids	Triterpene	1.03E+00	7.25E-03	1.10E-02	7.54E-02	-3.73E+00	down
DL-3-Phenyllactic acid	Phenolic acids	Phenolic acids	1.04E+00	7.27E-03	1.10E-02	2.07E+01	4.37E+00	up
3',6'-Dihydroxy-3,4,2',4'-Tetramethoxychalcone	Flavonoids	Chalcones	1.04E+00	7.31E-03	1.10E-02	8.07E-02	-3.63E+00	down
Histidinol	Alkaloids	Alkaloids	1.02E+00	7.33E-03	1.10E-02	3.30E+00	1.72E+00	up
3-(Cyclohexen-1-yl)-2-hydroxy-4a,5-dimethyl-2,3,4,5,6,7,8,8a-	Others	Others	1.04E+00	7.37E-03	1.11E-02	2.96E-02	-5.08E+00	down

octahydronaphthalen-1-one								
Protocatechualdehyde	Others	Aldehyde compounds	1.04E+00	7.40E-03	1.11E-02	3.18E-02	-4.98E+00	down
Erythro-Guaiacylglycerol	Phenolic acids	Phenolic acids	1.04E+00	7.42E-03	1.11E-02	6.40E+00	2.68E+00	up
Hispanolone	Terpenoids	Ditepenoids	1.04E+00	7.67E-03	1.15E-02	1.10E-01	-3.19E+00	down
1,7-diphenyl-4,6-diene-3-heptanone	Others	Ketone compounds	1.00E+00	7.72E-03	1.15E-02	1.97E-01	-2.34E+00	down
Evodol	Terpenoids	Triterpene	1.04E+00	7.74E-03	1.15E-02	1.58E+01	3.98E+00	up
Triethylamine	Alkaloids	Alkaloids	1.04E+00	7.77E-03	1.15E-02	5.69E+00	2.51E+00	up
7-Hydroxycoumarin;Umbelliferone	Lignans and Coumarins	Coumarins	1.03E+00	7.79E-03	1.16E-02	3.09E-01	-1.69E+00	down
JiangxiBaiyingsu I	Terpenoids	Sesquiterpenoids	1.04E+00	7.95E-03	1.18E-02	1.43E-01	-2.81E+00	down
3-Indolepropionic acid	Alkaloids	Plumerane	1.04E+00	7.98E-03	1.18E-02	5.70E+00	2.51E+00	up

6-Hydroxy-3,20-dioxo-30-norlupane-28-oic acid	Terpenoids	Triterpene	1.04E+00	7.99E-03	1.18E-02	1.71E-01	-2.54E+00	down
Pinoresinol*	Lignans and Coumarins	Lignans	1.04E+00	8.11E-03	1.19E-02	6.32E+00	2.66E+00	up
Aflatoxin M4	Others	Lactones	1.04E+00	8.11E-03	1.19E-02	7.28E+00	2.86E+00	up
Ganschisandrin	Lignans and Coumarins	Lignans	1.04E+00	8.25E-03	1.21E-02	9.59E-02	-3.38E+00	down
Benzyl β-primeveroside	Others	Others	1.00E+00	8.31E-03	1.22E-02	3.65E+00	1.87E+00	up
4'-Hydroxy-5,6,7-trimethoxyflavone	Flavonoids	Flavones	1.04E+00	8.34E-03	1.22E-02	6.54E+00	2.71E+00	up
phenylacetic acid	Phenolic acids	Phenolic acids	1.03E+00	8.39E-03	1.23E-02	3.27E-01	-1.61E+00	down
Macrophypene B	Terpenoids	Ditepenoids	1.04E+00	8.45E-03	1.23E-02	3.09E-02	-5.01E+00	down
Vanillic acid	Phenolic acids	Phenolic acids	1.03E+00	8.53E-03	1.24E-02	3.77E+00	1.91E+00	up
Piperidine	Alkaloids	Piperidine alkaloids	1.04E+00	8.85E-03	1.28E-02	5.27E+00	2.40E+00	up

1-Hydroxyanthraquinone	Quinones	Anthraquinone	1.04E+00	8.91E-03	1.29E-02	5.54E-02	-4.17E+00	down
O-MethylNaringenin-8-C-arabinoside	Flavonoids	Flavanones	1.02E+00	9.01E-03	1.30E-02	2.45E-01	-2.03E+00	down
Cyclohexane-1,3-dione	Others	Ketone compounds	1.04E+00	9.04E-03	1.30E-02	7.21E+00	2.85E+00	up
Trans-hinokiresinol	Lignans and Coumarins	Lignans	1.04E+00	9.39E-03	1.35E-02	8.23E+00	3.04E+00	up
Hydroxytyrosol	Others	Alcohol compounds	1.04E+00	9.51E-03	1.36E-02	4.65E+00	2.22E+00	up
Butyl isobutyl phthalate*	Phenolic acids	Phenolic acids	1.00E+00	9.56E-03	1.36E-02	3.15E-01	-1.67E+00	down
Protocatechuic Acid Methyl Ester	Phenolic acids	Phenolic acids	1.04E+00	9.56E-03	1.36E-02	6.23E+00	2.64E+00	up

15,16-Bisnor-13-oxo-8(17),11-labdadien-19-ol	Terpenoids	Sesquiterpenoids	1.04E+00	9.67E-03	1.38E-02	2.10E-02	-5.57E+00	down
N-Acetyl-5-hydroxytryptamine	Alkaloids	Plumerane	1.04E+00	9.96E-03	1.42E-02	5.99E+00	2.58E+00	up
Hesperetin-5-O-glucoside	Flavonoids	Flavanones	1.04E+00	1.03E-02	1.46E-02	5.78E+00	2.53E+00	up
16,23:16,30-Diepoxydammar-24-ene-3,20-diol (Jujubogenin)*	Terpenoids	Triterpene	1.04E+00	1.03E-02	1.46E-02	9.16E-02	-3.45E+00	down
Homovanillic acid; 4-Hydroxy-3-methoxyphenylacetic acid	Phenolic acids	Phenolic acids	1.04E+00	1.05E-02	1.49E-02	5.94E+00	2.57E+00	up
Hispidulin (5,7,4'-Trihydroxy-6-	Flavonoids	Flavones	1.04E+00	1.05E-02	1.49E-02	2.54E-02	-5.30E+00	down

methoxyflavone) *								
dihydro-4-hydroxy-5-hydroxymethyl-2(3H)-furanone	Others	Others	1.04E+00	1.06E-02	1.49E-02	6.88E+00	2.78E+00	up
Rengyoxide	Others	Others	1.01E+00	1.08E-02	1.52E-02	4.09E+00	2.03E+00	up
5-Hydroxy-7,8,2',6'-Tetramethoxyflavone (Altisin)	Flavonoids	Flavones	1.04E+00	1.08E-02	1.52E-02	6.99E+00	2.81E+00	up
Methyleugenol	Others	Others	1.04E+00	1.09E-02	1.52E-02	8.59E-02	-3.54E+00	down
3-Oxo-19-hydroxyurs-12-en-28-oic acid (Pomonic acid)	Terpenoids	Triterpene	1.04E+00	1.10E-02	1.54E-02	1.38E-01	-2.86E+00	down
5-hydroxyaloin	Quinones	Anthraquinone	1.04E+00	1.10E-02	1.54E-02	5.70E+00	2.51E+00	up
Isoferulic Acid*	Phenolic acids	Phenolic acids	1.04E+00	1.14E-02	1.58E-02	6.14E+00	2.62E+00	up
Hexyl Hydrogen Sulfate	Others	Others	1.02E+00	1.14E-02	1.59E-02	2.16E-03	-8.85E+00	down

3-pyridine-methanol-O-β-D-glucopyranosyl	Alkaloids	Pyridine alkaloids	1.03E+00	1.16E-02	1.61E-02	1.90E+01	4.24E+00	up
3-Hydroxymandelate	Phenolic acids	Phenolic acids	1.04E+00	1.19E-02	1.64E-02	1.23E-01	-3.02E+00	down
Kaempferol-3-O-(6"-p-Coumaroyl)glucoside (Tiliroside)*	Flavonoids	Flavonols	1.04E+00	1.22E-02	1.68E-02	6.03E+00	2.59E+00	up
Coumurrayin	Lignans and Coumarins	Coumarins	1.04E+00	1.24E-02	1.71E-02	1.06E-01	-3.24E+00	down
3-Hydroxyanthranilic acid	Alkaloids	Alkaloids	1.04E+00	1.24E-02	1.71E-02	7.07E+00	2.82E+00	up
Morusin	Flavonoids	Other Flavonoids	1.04E+00	1.28E-02	1.75E-02	7.83E-02	-3.67E+00	down
11α-hydroxy-3-oxo-4(5),6(7)-diene-eudesman-12-ol	Terpenoids	Sesquiterpenoids	1.04E+00	1.28E-02	1.75E-02	9.18E-02	-3.45E+00	down

dalbergin	Lignans and Coumarins	Coumarins	1.04E+00	1.28E-02	1.75E-02	2.68E-02	-5.22E+00	down
16-oxo-21β-hydroxyserrat-14-en-3α-yl acetate	Terpenoids	Triterpene	1.04E+00	1.28E-02	1.76E-02	1.62E-01	-2.63E+00	down
Maesol	Others	Others	1.04E+00	1.30E-02	1.77E-02	5.84E-02	-4.10E+00	down
2-hydroxy-2-isobutylsuccinic acid	Others	Others	1.04E+00	1.31E-02	1.78E-02	7.98E+00	3.00E+00	up
Epigallocatechin-3-O-(3-O-methyl)gallate	Flavonoids	Flavanols	1.04E+00	1.31E-02	1.78E-02	7.23E+00	2.85E+00	up
clerodermic acid	Terpenoids	Monoterpenoids	1.04E+00	1.31E-02	1.78E-02	2.98E-02	-5.07E+00	down
Dihydrokaempferide	Flavonoids	Flavanonols	1.04E+00	1.31E-02	1.78E-02	5.67E+00	2.50E+00	up
(1S,3S)-1-Methyl-1,2,3,4-tetrahydro-β-carboline-3-carboxylic acid*	Alkaloids	Plumerane	1.04E+00	1.32E-02	1.78E-02	6.15E+00	2.62E+00	up

O-Anisic acid (2-Methoxybenzoic acid)*	Phenolic acids	Phenolic acids	1.03E+00	1.32E-02	1.78E-02	3.09E-01	-1.69E+00	down
23-Hydroxy-3-oxoolean-12-en-28-oic acid (Hederagonic acid)	Terpenoids	Triterpene	1.04E+00	1.32E-02	1.78E-02	7.13E-02	-3.81E+00	down
7-Hydroxy-4-methylcoumarin	Lignans and Coumarins	Coumarins	1.04E+00	1.33E-02	1.79E-02	6.71E+00	2.75E+00	up
4-Methylbenzoic acid	Phenolic acids	Phenolic acids	1.03E+00	1.34E-02	1.80E-02	1.56E-01	-2.68E+00	down
Quercetin-4'-O-glucoside (Spiraeoside)*	Flavonoids	Flavonols	1.04E+00	1.35E-02	1.82E-02	6.73E+00	2.75E+00	up
Quercetin-3-O-apiosyl(1→2)galactoside	Flavonoids	Flavonols	1.04E+00	1.37E-02	1.84E-02	1.12E+01	3.49E+00	up
8-Hydroxy-4',5,7-	Flavonoids	Flavones	1.04E+00	1.39E-02	1.86E-02	1.08E+01	3.43E+00	up

trimethoxyflavone*								
Secoisolariciresinol	Lignans and Coumarins	Lignans	1.04E+00	1.39E-02	1.86E-02	6.50E+00	2.70E+00	up
neopetasane*	Terpenoids	Sesquiterpenoids	1.03E+00	1.40E-02	1.87E-02	5.58E-02	-4.16E+00	down
2,3-Dihydroxyoleana-11,13(18)-dien-28-oic acid (Camaldulenic acid)	Terpenoids	Triterpene	1.04E+00	1.41E-02	1.88E-02	7.52E-02	-3.73E+00	down
Quercetin-5-O-glucuronide*	Flavonoids	Flavonols	1.04E+00	1.42E-02	1.90E-02	1.07E+01	3.42E+00	up
(Rel 5S,6R,8R,9R,10S,13S,15S,16R)-6-Acetoxy-9,13;15,16 diepoxy-15,16-	Terpenoids	Ditepenoids	1.03E+00	1.43E-02	1.91E-02	7.97E-02	-3.65E+00	down

dimethoxylabdane								
Quercetin-3-O-rhamnoside(Quercitrin)	Flavonoids	Flavonols	1.04E+00	1.44E-02	1.91E-02	5.63E+00	2.49E+00	up
β-Anhydroicaritin	Flavonoids	Other Flavonoids	1.04E+00	1.45E-02	1.92E-02	1.29E-01	-2.95E+00	down
Epicatechin gallate*	Flavonoids	Flavanols	1.04E+00	1.56E-02	2.06E-02	7.73E+00	2.95E+00	up
3-(7,10-Pentadecadienyl)-1,2-benzenediol*	Phenolic acids	Phenolic acids	1.04E+00	1.57E-02	2.07E-02	1.38E-01	-2.85E+00	down
3-Oxo-9,19-cyclolanost-24-en-26-oic acid (Mangiferonic acid)*	Terpenoids	Triterpene	1.04E+00	1.57E-02	2.07E-02	1.84E-02	-5.77E+00	down
N-(4'-O-glycosyl)-feruloyl agmatine	Alkaloids	Phenolamine	1.04E+00	1.59E-02	2.09E-02	8.16E+00	3.03E+00	up

5-O-Methyllatifolin*	Others	Others	1.01E+00	1.61E-02	2.11E-02	1.89E-01	-2.41E+00	down
Ethyl (10Z,13Z)-hexadeca-10,13-dienoate	Others	Others	1.01E+00	1.62E-02	2.13E-02	1.68E-01	-2.57E+00	down
3-(2-amino-1-hydroxyethyl)-1H-indol-5-ol	Alkaloids	Alkaloids	1.04E+00	1.63E-02	2.13E-02	1.83E+01	4.20E+00	up
(5Z,10E,14S)-14-hydroxy-2,6,10-trimethylpentadeca-5,10-dien-4-one	Others	Others	1.02E+00	1.63E-02	2.13E-02	2.10E-01	-2.25E+00	down
2,6-Dimethoxyhydroquinone-1-O-glucoside	Others	Others	1.04E+00	1.64E-02	2.14E-02	1.02E+01	3.35E+00	up
Diethanolamine	Alkaloids	Alkaloids	1.03E+00	1.65E-02	2.15E-02	4.37E-01	-1.19E+00	down
8-ethylnorlobelol	Alkaloids	Piperidine alkaloids	1.04E+00	1.67E-02	2.16E-02	1.04E-01	-3.27E+00	down

Salvigenin 5-O-glucoside	Flavonoids	Flavones	1.04E+00	1.67E-02	2.16E-02	7.10E+00	2.83E+00	up
Vnilloylmalic acid	Phenolic acids	Phenolic acids	1.03E+00	1.67E-02	2.17E-02	6.41E+00	2.68E+00	up
1-O-Galloyl-rhamnose	Phenolic acids	Phenolic acids	1.04E+00	1.71E-02	2.21E-02	9.80E+00	3.29E+00	up
3-Hydroxy-9,19-cyclolanost-24-en-26-oic acid (Mangiferolic acid)	Terpenoids	Triterpene	1.04E+00	1.73E-02	2.24E-02	9.36E-02	-3.42E+00	down
Indole-5-carboxylic acid*	Alkaloids	Plumerane	1.03E+00	1.75E-02	2.26E-02	1.21E-01	-3.05E+00	down
5-Hydroxy-3,3',4',5',7-Pentamethoxyflavone*	Flavonoids	Flavones	1.03E+00	1.76E-02	2.27E-02	2.17E-01	-2.20E+00	down
Des-O-Methyllasiodiplodin	Others	Lactones	1.00E+00	1.78E-02	2.29E-02	3.19E-01	-1.65E+00	down

Quercetin-3-O-(4''-O-glucosyl)rhamnoside*	Flavonoids	Flavonols	1.03E+00	1.81E-02	2.32E-02	6.50E+00	2.70E+00	up
1-[1-O-5(3,4-methylenedioxyphenyl)-2E,4E-pentadienyl]-pyrrolidine	Alkaloids	Pyrrole alkaloids	1.04E+00	1.82E-02	2.34E-02	4.85E-02	-4.37E+00	down
Quercetin-4'-O-glucuronide*	Flavonoids	Flavonols	1.04E+00	1.84E-02	2.36E-02	6.51E+00	2.70E+00	up
8-Methoxykaempferol-7-O-rhamnoside	Flavonoids	Flavonols	1.04E+00	1.87E-02	2.39E-02	7.77E+00	2.96E+00	up
Phloroglucinol; 1,3,5-Benzenetriol	Others	Others	1.03E+00	1.95E-02	2.48E-02	6.58E+00	2.72E+00	up
1,2,9,10-tetraethoxy-6-	Alkaloids	Alkaloids	1.04E+00	1.95E-02	2.48E-02	1.37E+01	3.78E+00	up

methyl-5,6,6a,7-tetrahydro-4H-dibenzo[de,g]quinoline								
3-Hydroxyurs-12-en-28-oic acid (3-Epiursolic acid)*	Terpenoids	Triterpene	1.04E+00	1.98E-02	2.51E-02	7.09E-02	-3.82E+00	down
Vanillyl alcohol	Phenolic acids	Phenolic acids	1.03E+00	2.00E-02	2.53E-02	6.61E+00	2.72E+00	up
Diphenylamine	Alkaloids	Alkaloids	1.04E+00	2.01E-02	2.53E-02	1.15E-01	-3.12E+00	down
Neochlorogenic acid (5-O-Caffeoylquinic acid)	Phenolic acids	Phenolic acids	1.03E+00	2.02E-02	2.55E-02	6.18E+00	2.63E+00	up
Creatinine	Alkaloids	Alkaloids	1.02E+00	2.06E-02	2.59E-02	2.04E+00	1.03E+00	up
Naringenin-6-C-Glucoside	Flavonoids	Flavanones	1.03E+00	2.06E-02	2.59E-02	6.81E+00	2.77E+00	up
12,13-Dihydroursolic acid	Terpenoids	Triterpene	1.03E+00	2.09E-02	2.62E-02	1.73E-01	-2.53E+00	down

3-Hydroxyurs-12-en-28-oic acid (Ursolic acid)*	Terpenoids	Triterpene	1.04E+00	2.10E-02	2.62E-02	6.86E-02	-3.86E+00	down
γ-L-glutaminy-3,4-dihydroxybenzene(GDHB)	Alkaloids	Alkaloids	1.03E+00	2.10E-02	2.63E-02	6.43E+00	2.68E+00	up
Helicide	Phenolic acids	Phenolic acids	1.03E+00	2.23E-02	2.77E-02	6.20E+00	2.63E+00	up
Didymin (Isosakuranetin-7-O-rutinoside)*	Flavonoids	Flavanones	1.03E+00	2.24E-02	2.78E-02	6.68E+00	2.74E+00	up
N-(4-O-(Glucosyl)-E-feruloyl)-tyramine	Alkaloids	Phenolamine	1.03E+00	2.25E-02	2.80E-02	6.84E+00	2.77E+00	up
4-Hydroxyacetophenone	Others	Ketone compounds	1.04E+00	2.29E-02	2.84E-02	5.58E-02	-4.16E+00	down
Minheryin D	Terpenoids	Ditepenoids	1.03E+00	2.33E-02	2.89E-02	3.43E-02	-4.86E+00	down
Nootkatone*	Terpenoids	Sesquiterpenoids	1.03E+00	2.35E-02	2.90E-02	1.46E-01	-2.78E+00	down

Sugiol methyl ether	Terpenoids	Ditepenoids	1.03E+00	2.38E-02	2.93E-02	1.63E-01	-2.62E+00	down
3,5-Dimethyl-2,3-dihydrobenzofuran	Others	Others	1.02E+00	2.38E-02	2.93E-02	2.70E-01	-1.89E+00	down
β-Mangostin	Flavonoids	Flavones	1.03E+00	2.50E-02	3.06E-02	1.60E-01	-2.65E+00	down
6-O-Acetyl-3-O-(4-O-malonyl)-xylosylcycloastragenol	Terpenoids	Triterpene Saponin	1.02E+00	2.56E-02	3.14E-02	1.29E-02	-6.28E+00	down
N,N-Dimethylformamide	Alkaloids	Alkaloids	1.02E+00	2.59E-02	3.17E-02	2.90E-01	-1.79E+00	down
3-Hydroxycycloarta-24-ene-26-oic acid (Isomangiferolic acid)*	Terpenoids	Triterpene	1.04E+00	2.59E-02	3.17E-02	7.19E-02	-3.80E+00	down

9,11,13,15-Octadecatetraenal	Others	Aldehyde compounds	1.03E+00	2.60E-02	3.18E-02	1.45E-01	-2.78E+00	down
5-O-Methylgalloylquinic Acid	Phenolic acids	Phenolic acids	1.03E+00	2.62E-02	3.19E-02	6.35E+00	2.67E+00	up
6-O-Caffeoyl-D-glucose*	Phenolic acids	Phenolic acids	1.03E+00	2.70E-02	3.29E-02	6.41E+00	2.68E+00	up
Phenyl acetate	Others	Others	1.03E+00	2.78E-02	3.38E-02	6.10E-02	-4.03E+00	down
4,6-dimethoxy-2-methoxyphenyl-1-O-beta-D-glucopyranoside	Others	Others	1.03E+00	2.82E-02	3.42E-02	6.01E+00	2.59E+00	up
Quercetagetin; 3,3',4',5,6,7-Hexahydroxyflavone	Flavonoids	Flavonols	1.03E+00	2.82E-02	3.42E-02	8.54E+00	3.09E+00	up
Nigramide M	Alkaloids	Piperidine alkaloids	1.02E+00	2.83E-02	3.43E-02	2.41E-01	-2.05E+00	down
Sibiricose A3	Phenolic acids	Phenolic acids	1.03E+00	2.87E-02	3.47E-02	8.73E+00	3.13E+00	up

3-(11,13-Pentadecadienyl)-(Z,E)1,2-Benzenediol*	Phenolic acids	Phenolic acids	1.03E+00	2.87E-02	3.47E-02	1.27E-01	-2.97E+00	down
3-Oxours-12-en-28-oic acid (Ursonic acid)*	Terpenoids	Triterpene	1.03E+00	2.89E-02	3.49E-02	7.79E-02	-3.68E+00	down
D-Friedoolean-14-ene-3,28-diol (Myricadoil)	Terpenoids	Triterpene	1.03E+00	2.96E-02	3.56E-02	1.50E-01	-2.73E+00	down
serratenediol	Terpenoids	Triterpene	1.03E+00	2.97E-02	3.58E-02	1.47E-01	-2.77E+00	down
Dihydroartemisinin	Terpenoids	Sesquiterpenoids	1.04E+00	3.04E-02	3.65E-02	5.33E-02	-4.23E+00	down
Epigallocatechin-3-O-gallate	Flavonoids	Flavanols	1.03E+00	3.08E-02	3.70E-02	6.83E+00	2.77E+00	up
N-Oleoylethanolamine	Alkaloids	Alkaloids	1.04E+00	3.11E-02	3.73E-02	1.27E-02	-6.30E+00	down
6α,10α-Dihydroxy-1-	Terpenoids	Sesquiterpenoids	1.01E+00	3.14E-02	3.76E-02	3.51E-01	-1.51E+00	down

oxoeremophila-7(11),8(9)-dien-12,8-olide								
5,6,2',3',4',6'-Hexamethoxyflavone	Flavonoids	Flavones	1.03E+00	3.23E-02	3.85E-02	7.75E+00	2.95E+00	up
Olean-13(18)-en-3-one (δ-Amyrenone)	Terpenoids	Triterpene	1.03E+00	3.26E-02	3.88E-02	1.49E-01	-2.74E+00	down
α-Ionone	Terpenoids	Monoterpenoids	1.03E+00	3.34E-02	3.95E-02	1.40E-01	-2.84E+00	down
Fusosteride A	Steroids	Steroid	1.03E+00	3.34E-02	3.96E-02	1.25E-01	-3.00E+00	down
N-Benzoyltyramine	Alkaloids	Phenolamine	1.03E+00	3.35E-02	3.96E-02	6.69E+00	2.74E+00	up
5-Hydroxyflavone	Flavonoids	Flavones	1.03E+00	3.36E-02	3.96E-02	1.09E-01	-3.20E+00	down
3,4-Dihydroxybenzoic Acid Ethyl Ester	Phenolic acids	Phenolic acids	1.04E+00	3.42E-02	4.03E-02	5.66E-02	-4.14E+00	down

(Protocatechuic acid ethyl ester)								
lancilactone A	Terpenoids	Terpene	1.04E+00	3.49E-02	4.11E-02	2.40E-03	-8.70E+00	down
3,5,7-Trihydroxy-6,8-dimethyl-3-(4'-hydroxybenzyl)-chroman-4-one (Polygonatone C)	Flavonoids	Other Flavonoids	1.03E+00	3.61E-02	4.23E-02	4.04E-02	-4.63E+00	down
isovalerylshikonin	Quinones	Quinones	1.03E+00	3.73E-02	4.36E-02	1.20E-01	-3.06E+00	down
Gentisic acid 2-O-(6''-O-p-coumaroyl)Glucoside	Phenolic acids	Phenolic acids	1.03E+00	4.00E-02	4.64E-02	6.71E+00	2.75E+00	up
Olean-12-en-3-one (β-Amyrone)	Terpenoids	Triterpene	1.03E+00	4.04E-02	4.68E-02	1.11E-01	-3.17E+00	down
7-(1,2-Dihydroxyethyl)-2-hydroxy-1-	Terpenoids	Ditepenoids	1.03E+00	4.05E-02	4.68E-02	4.31E-02	-4.54E+00	down

(hydroxymethyl)-1,4b,7-trimethyl-2,5,6,8,8a,9,10,10a-octahydrophenanthren-3-one								
Sophoranochromene	Flavonoids	Flavanones	1.03E+00	4.17E-02	4.82E-02	3.94E-02	-4.67E+00	down
3-Methylbutyl 6-O-(alpha-L-arabinopyranosyl)-beta-D-glucopyranoside	Others	Alcohol compounds	1.02E+00	4.22E-02	4.87E-02	7.91E+00	2.98E+00	up
2,3-Dihydroxy-5α-pregn-17(Z)-en-16-one	Steroids	Steroid	1.03E+00	4.27E-02	4.92E-02	1.07E-01	-3.22E+00	down
α-Amyrenone	Terpenoids	Triterpene	1.02E+00	4.39E-02	5.04E-02	1.32E-01	-2.93E+00	down
Regaloside J	Phenolic acids	Phenolic acids	1.02E+00	4.44E-02	5.08E-02	7.21E+00	2.85E+00	up
Ardisianone E	Quinones	Quinones	1.04E+00	4.53E-02	5.18E-02	4.49E+01	5.49E+00	up
Ergotamine	Alkaloids	Plumerane	1.04E+00	4.56E-02	5.20E-02	2.29E+02	7.84E+00	up

Tectochrysin	Flavonoids	Flavones	1.03E+00	4.66E-02	5.31E-02	1.09E-01	-3.20E+00	down
13-Methyl-27-norolean-14-en-3-ol (Taraxerol)	Terpenoids	Triterpene	1.02E+00	4.91E-02	5.59E-02	1.23E-01	-3.02E+00	down
